# Simultaneous DNA and RNA Mapping of Somatic Mitochondrial Mutations across Diverse Human Cancers

**DOI:** 10.1371/journal.pgen.1005333

**Published:** 2015-06-30

**Authors:** James B. Stewart, Babak Alaei-Mahabadi, Radhakrishnan Sabarinathan, Tore Samuelsson, Jan Gorodkin, Claes M. Gustafsson, Erik Larsson

**Affiliations:** 1 Max Planck Institute for Biology of Ageing, Cologne, Germany; 2 Institute of Biomedicine, Sahlgrenska Academy, University of Gothenburg, Gothenburg, Sweden; 3 Center for non-coding RNA in Technology and Health, IKVH, University of Copenhagen, Frederiksberg, Denmark; St Jude Children's Research Hospital, UNITED STATES

## Abstract

Somatic mutations in the nuclear genome are required for tumor formation, but the functional consequences of somatic mitochondrial DNA (mtDNA) mutations are less understood. Here we identify somatic mtDNA mutations across 527 tumors and 14 cancer types, using an approach that takes advantage of evidence from both genomic and transcriptomic sequencing. We find that there is selective pressure against deleterious coding mutations, supporting that functional mitochondria are required in tumor cells, and also observe a strong mutational strand bias, compatible with endogenous replication-coupled errors as the major source of mutations. Interestingly, while allelic ratios in general were consistent in RNA compared to DNA, some mutations in tRNAs displayed strong allelic imbalances caused by accumulation of unprocessed tRNA precursors. The effect was explained by altered secondary structure, demonstrating that correct tRNA folding is a major determinant for processing of polycistronic mitochondrial transcripts. Additionally, the data suggest that tRNA clusters are preferably processed in the 3′ to 5′ direction. Our study gives insights into mtDNA function in cancer and answers questions regarding mitochondrial tRNA biogenesis that are difficult to address in controlled experimental systems.

## Author Summary

According to the so-called “tRNA punctuation model”, tRNA processing is key to generating all mature mitochondrial mRNAs. However, the process is difficult to study *in vivo*, since standard tools for genetic manipulation are not applicable to mitochondria. Here, we circumvent this problem by using a large compendium of naturally occurring genetic perturbations, derived from human tumor sequencing data. We identify somatic mitochondrial mutations across hundreds of human tumors using an approach that simultaneously takes advantage of both genomic and transcriptomic sequencing. This enables us to compare the allele frequency in DNA and RNA for each mutation. Our data reveals that some mutations in mitochondrial tRNAs are associated with strong accumulation of immature tRNA precursors, indicative of impaired tRNA mutaration. We find that intact tRNA secondary structure is a major requirement for correct maturation, and that mutations affecting tRNA folding can impair maturation of not only the affected tRNA, but also neighboring gene transcripts. Mutations in mitochondrial tRNAs underlie a range of disease conditions, and our findings may help to explain why mutations in the same tRNA can present different phenotypes. Our results additionally support that there is selective pressure against mutations affecting oxidative phosphorylation, showing that functional mitochondria are required in many tumor cells.

## Introduction

Under normal conditions, mammalian cells rely on oxidative phosphorylation in mitochondria to generate most of their supply of adenosine triphosphate (ATP). Cancer cells, in contrast, typically show increased production of ATP through non-oxidative breakdown of glucose in the cytoplasm (the Warburg effect) [1], and reprogramming of energy metabolism is now considered a hallmark of cancer [2]. However, in spite of the Warburg effect, the majority of ATP is in many tumors still produced via the respiratory chain, and there are also examples of cancer cells with increased dependence on oxidative phosphorylation [3].

All tumors arise as a consequence of acquired somatic genetic alterations followed by microevolutionary selection [4]. The study of mutations in tumors has in recent years been greatly facilitated by massively parallel sequencing, which has provided numerous insights into the changes in the nuclear genome that underlie cancer [5,6]. However, despite the fact that altered mitochondrial function is a characteristic property of tumor cells, only a few studies have characterized somatic mutations in the mitochondrial genome in cancer using high-throughput methodologies [7,8]. These studies have demonstrated that somatic mtDNA mutations are common in tumor cells [7,8], and that there is a negative selection against mutations that results in protein truncation, i.e. impair proper oxidative phosphorylation [8].

The 16.6 kb circular human mitochondrial genome harbors 37 genes, of which 13 are protein-coding, two codes for rRNA, whereas the rest codes for mitochondrial tRNAs (mt-tRNAs). Transcription is initiated from two major promoter regions, one for the “light” and one for the “heavy” strand of the DNA molecule (L and H, respectively), generating long polycistronic transcripts that contain the genetic information of the respective strand. The curious interspacing of the tRNA genes between the mRNAs and rRNAs lead to the hypothesized “tRNA punctuation model”, whereby the tRNAs are specifically recognized and cleaved out of these polycistronic transcripts, with the remaining RNAs being matured to the mRNAs or rRNAs [9,10]. Since then, a more complete understanding of the mRNA maturation processing processes and its complexities in mammalian mitochondria has developed [11,12].

In the polycistronic transcripts, the mt-tRNAs encoding regions are identified and processed by a 5´ acting mitochondrial RNaseP, a heteromer of the *MRPP1- 3*, *HSD10* gene products [13–15], which is followed by a 3´ processing event carried out by the mitochondrial RNaseZ (*ELAC2*) [16,17] and, possibly, *PTCD1* [18]. It was hypothesized early on that the structure and not the sequence of the tRNA may represent the main signal for recognition by the processing enzymes [9], a notion supported by the fact that the same RNase P appears to cleave all mt-tRNA precursors [13]. Interestingly, pathogenic mt-tRNA variants appear to often be located in tRNA stem regions, suggestive of an impact on secondary structure [19], and there are some examples in the literature of mutations that impair processing while at the same time affecting mt-tRNA structure [20,21]. Unfortunately, it is has been difficult to study this phenomenon in a more systematic way due to difficulty of performing reverse genetics in mitochondria, and the relationship between pre-tRNA structure and processing *in vivo* therefore remains incompletely understood [22].

Here we make use of whole-genome sequencing (WGS) data from The Cancer Genome Atlas (TCGA) consortium to map somatic mitochondrial mutations in 527 tumors from 14 types of human cancer. Since most of the mitochondrial genome is transcribed and polyadenylated, we could further refine our mutational map by requiring mutations to be detectable also in matched transcriptome sequencing (RNA-seq) data from the same tumors. This approach enabled a comparison of the allelic ratios in DNA to RNA for all mutations, allowing detection of allelic imbalances that arise when genetic alleles are processed at different rates at the level of RNA. We found that this was an effective way of pinpointing mutations that lead to tRNA maturation defects, making it possible to use our compendium of somatic mitochondrial DNA (mtDNA) mutations to gain insight into mt-tRNA processing.

## Results

### DNA/RNA mapping somatic mitochondrial mutations in 527 tumors

We screened 527 tumors, spanning 14 types of human cancer, for somatic mtDNA mutations using high-coverage WGS data from TCGA (**Table 1**; included samples/libraries are listed in **S1 Dataset**). Mutations were called by comparing tumors to non-tumor samples from the same individuals. Due to the multi-copy nature of mtDNA, most mutations showed very high sequencing coverage (on average >5000 reads), effectively minimizing the risk of contamination from nuclear DNA pseudogenes of mitochondrial origin (**S1 Fig**). In addition, mutations were mapped in polyA+ RNA-seq from the same tumors, confirming 96% of the WGS-based mutations and resulting in a final set of 616 high-confidence mutations (564 single-nucleotide variants and 52 small indels) supported by both data types (detailed in **S2 Dataset**). Of the analyzed tumors, 335 (64%) had at least one mutation, and the average number of mutations per tumor (1.17) varied between 0.32 and 1.87 in the individual cancers (**Table 1**). 363 (59%) of mutations were in coding regions (69% missense, 19% synonymous, 7% frameshift, and 4% nonsense).

**Table 1:** Overview of included tumors and detected somatic mtDNA mutations.

Code	Cancer type	Tumors analyzed	Total mutations	Mutations/tumor	Fraction indels
BLCA	Bladder	23	43	1.87	7.0%
BRCA	Breast	100	123	1.23	7.3%
CRC	Colorectal	42	52	1.24	7.7%
GBM	Glioblastoma	37	8	0.22	0.0%
HNSC	Head & neck	29	29	1.00	13.8%
KICH	Kidney (chrom.)	15	17	1.13	17.6%
KIRC	Kidney (clear)	29	29	1.00	20.7%
LGG	Low-grade glioma	18	8	0.44	12.5%
LUAD	Lung (adeno)	46	65	1.41	3.1%
LUSC	Lung (squamus)	45	54	1.20	5.6%
PRAD	Prostate	19	6	0.32	16.7%
SKCM	Melanoma	42	62	1.48	8.1%
THCA	Thyroid	35	36	1.03	11.1%
UCEC	Uterus	47	84	1.79	8.3%
		527	616	1.17	8.4%

527 tumor/normal pairs from 14 cancer types were analyzed for somatic mutations (substitutions and small indels) in mtDNA based on high-coverage genomic sequencing data, considering mutations with allele frequency >15% in the tumor and <0.5% in the normal that additionally were confirmed in matched RNA-seq data from the same tumors.

**Fig 1:**
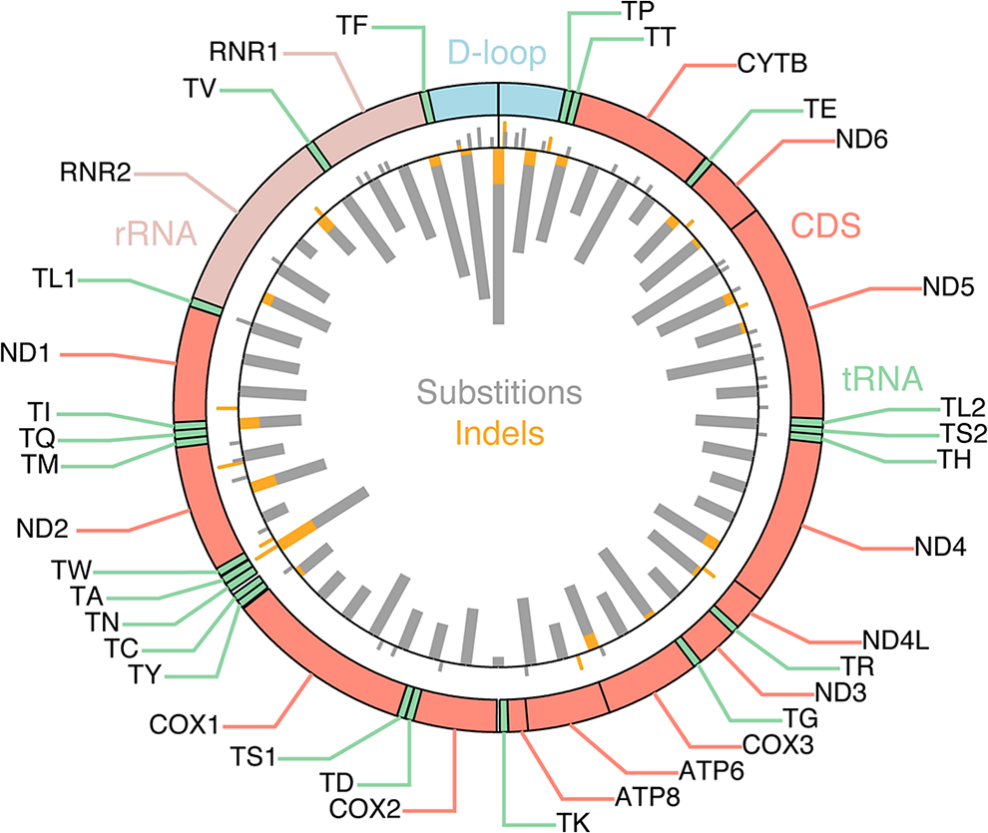
Mutational density across the mitochondrial genome. Inward-facing thick bars indicate the number of mutations per 331 nt segment (50 segments), with substitutions and indels shown in gray and orange, respectively. 616 somatic mutations, identified across 527 tumors, are shown. Outward-facing bars thin bars indicate individual recurrently mutated positions (> = 2 tumors).

Earlier high-throughput screens of somatic mitochondrial mutations have presented conflicting results regarding positive selection in protein-coding genes [7,8]. We found that the frequency of nonsynonymous mutations did not deviate significantly from the expectation (*P* = 0.084, **χ** ^2^ test with Yates correction), thus providing no clear evidence of positive selection (S1 Appendix). While several coding positions were recurrently mutated (**Fig 1**, outward-facing bars), these were preferably frameshift-causing expansions of mononucleotide repeats, including insertions at polyC stretches at 10,947–52 and 11,867–72 in the *ND4* gene (occurring in 5 tumors), and indels in a polyA stretch at position 12418–25 in the *ND5* gene (in 4 tumors). These hotspots are likely due to a high susceptibility to polymerase slippage errors rather than positive selection [23], although some of them have been described and attributed possible tumorigenic effects [24–26].

### Purifying selection acting on mtDNA in tumors

Deleterious coding mitochondrial variants are uncommon in normal tissues [27], confirmed by analysis of the normal samples included in this study (no nonsense and only a single frameshift mutation was detected). The frequencies of damaging somatic mutations reported above (7% frameshift and 4% nonsense) are thus suggestive of relaxed functional constraints in tumor tissues. A large meta-analysis of somatic mtDNA mutations in oncocytic tumors indeed found that the overall pattern of amino acid changes was compatible with a lack of purifying selection [28]. However, a recent study that took variant allele frequencies into account reported that deleterious somatic variants were suppressed in human cancers, suggestive of purifying selection against these reaching high levels of heteroplasmy [8].

**Fig 2:**
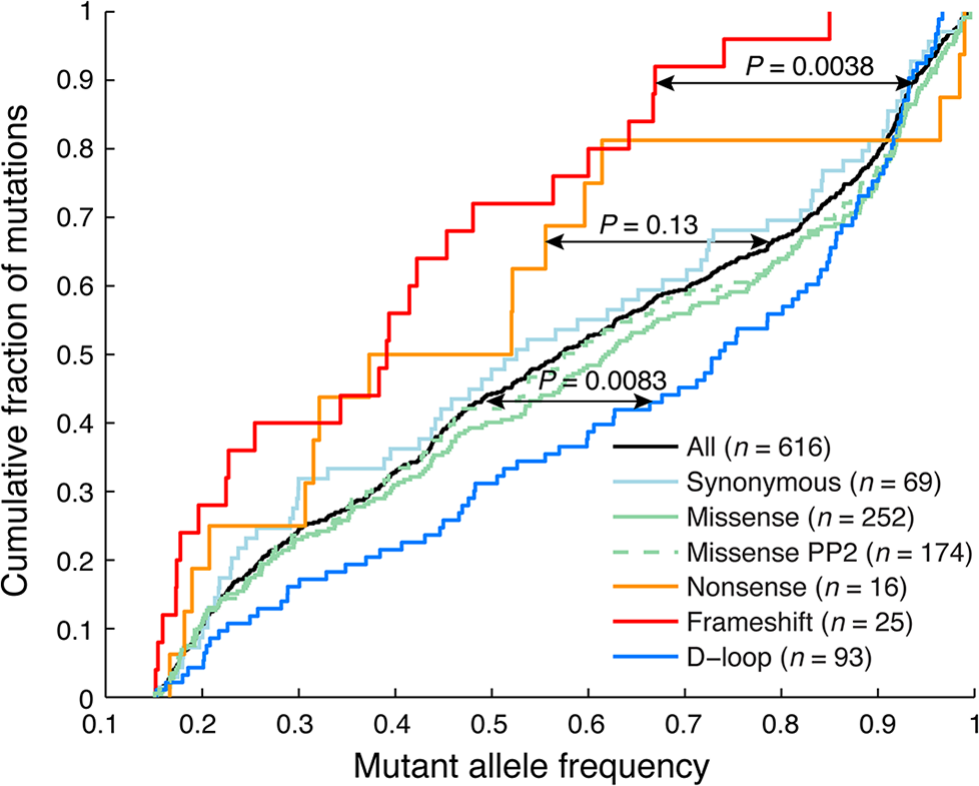
Frameshift indels show reduced heteroplasmy levels indicative of negative selection. Cumulative distributions of mutant allele frequencies (heteroplasmy levels) for different mutational categories. Frameshift indels showed significantly reduced levels of heteroplasmy, and never reach above 85%. A similar trend, although non-significant, was seen for missense (stop-inducing) mutations. In contrast, D-loop mutations, which in general should be more tolerable, showed significantly elevated levels. *P*-values were calculated using the two-sample Kolmogorov-Smirnov test, comparing the tumor set of interest to remaining samples. Missense PP2 refers to a subset of missense mutations predicted to be “probably damaging” by PolyPhen-2 [29].

In our DNA/RNA-based compendium of somatic mitochondrial mutations, we found that heteroplasmy levels varied greatly, at an average of 58.1% and with 8.0% of the 616 mutations reaching near-homoplasmy (mutant allele frequency >95%; **Fig 2**). However, frameshifting indels in coding regions, likely to have a strong negative functional impact, showed clearly reduced heteroplasmy levels (39.6%; *P* = 0.0038, two-sample Kolgomorov-Smirnov test), notably without ever reaching above 85%. A similar trend was seen for nonsense (premature stop) mutations (48.8%, *P* = 0.13), while synonymous and missense mutations were comparable to the full set, also when considering a subset with predicted likely functional impact as defined using the PolyPhen-2 method [29]. Somatic mutations were detected throughout the mitochondrial genome, but the density was clearly higher in the “hypervariable” D-loop region, where mutations are better tolerated [30] (**Fig 1**). The nucleotide composition in this region does not deviate considerably from other parts of the mitochondrial genome [30], and the result can thus be interpreted as if negative selection is less pronounced in the non-coding D-loop. In support of this, we found that D-loop mutations showed elevated heteroplasmy levels (average 65.8%) compared to non-D-loop mutations (P = 0.0083; **Fig 2**).

Taken together, our data confirms and extends recent results [8], supporting that purifying selection is at play in coding regions to maintain mitochondrial function in tumors, although to a lesser extent than in normal tissues. Importantly, while both studies make use of TCGA data, the sample overlap is small (30/527 tumors; 5.7%) and the results thus largely independent.

### Mutational signatures and replicative strand bias

**Fig 3:**
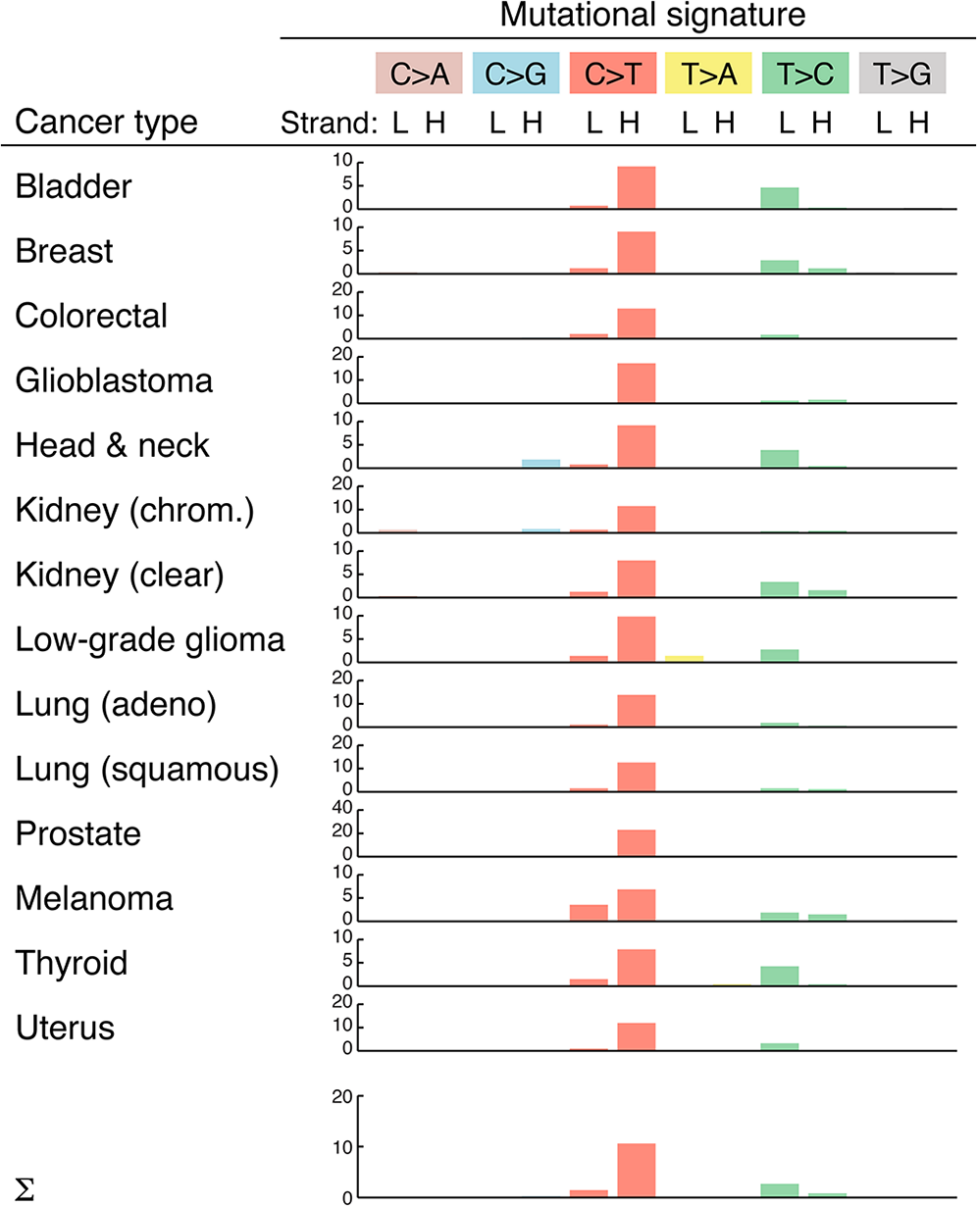
Overview of mtDNA mutational signatures. Substitution patterns (mutational signatures) are shown for each cancer type, which each substitution class labeled by the pyrimidine of the Watson-Crick pair [31] but with sense and antisense patterns shown separately to reveal strand biases. Bars indicate enrichment relative to the expected frequency (observed/expected ratio) for all possible substitutions, taking into account the nucleotide composition of mtDNA and assuming equal probability for all three substitutions. L, light strand; H, heavy strand.

Insights into the mutational processes that underlie somatic mutations can be gained by analyzing the sequence properties of the resulting substitutions [31]. We found that mutations preferably occurred at CG base pairs (65%; 1.5 expected frequency) in the form of C>T (or G>A) transitions (**S1 Dataset**), similar to the typical nuclear mutational profile in tumors [31]. A more detailed analysis revealed a strong strand bias, with C>T transitions on the H-strand occurring at 10.6 times the expected frequency (**Fig 3**). This confirms and closely matches the results of a recent analysis [8]. Strand bias was observed throughout the genome, except in the D-loop region, where C>T transitions were still overrepresented but not in a strand specific manner (**S2 Fig**). The observed strand bias is likely related to differences in the mode of replication of the two strands [32].

The mutational profile was highly consistent across all 14 cancers, although strand biases were reduced in melanoma (**Fig 3**). This could suggest a contribution from an exogenous mutagenic processes in this cancer; however, arguing against this interpretation, we failed to observe an elevation in the dipyrimidine signature typical of UV exposure [33] in the flanking bases. We conclude that endogenous replication errors or other replication-coupled effects are the dominant source of somatic mutations in tumor mtDNA.

### Insights into mt-tRNA processing from DNA/RNA allelic imbalances

The dual genomic/transcriptomic design of our study enabled us to compare allelic ratios (heteroplasmy levels) in DNA and RNA, to reveal imbalances that arise when genetic alleles are differentially transcribed or processed. In the nuclear genome, allelic imbalances arise frequently due to, for example, genomic imprinting or RNA surveillance (nonsense-mediated decay, NMD) [34], but no large-scale studies have been done in mitochondria.

**Fig 4:**
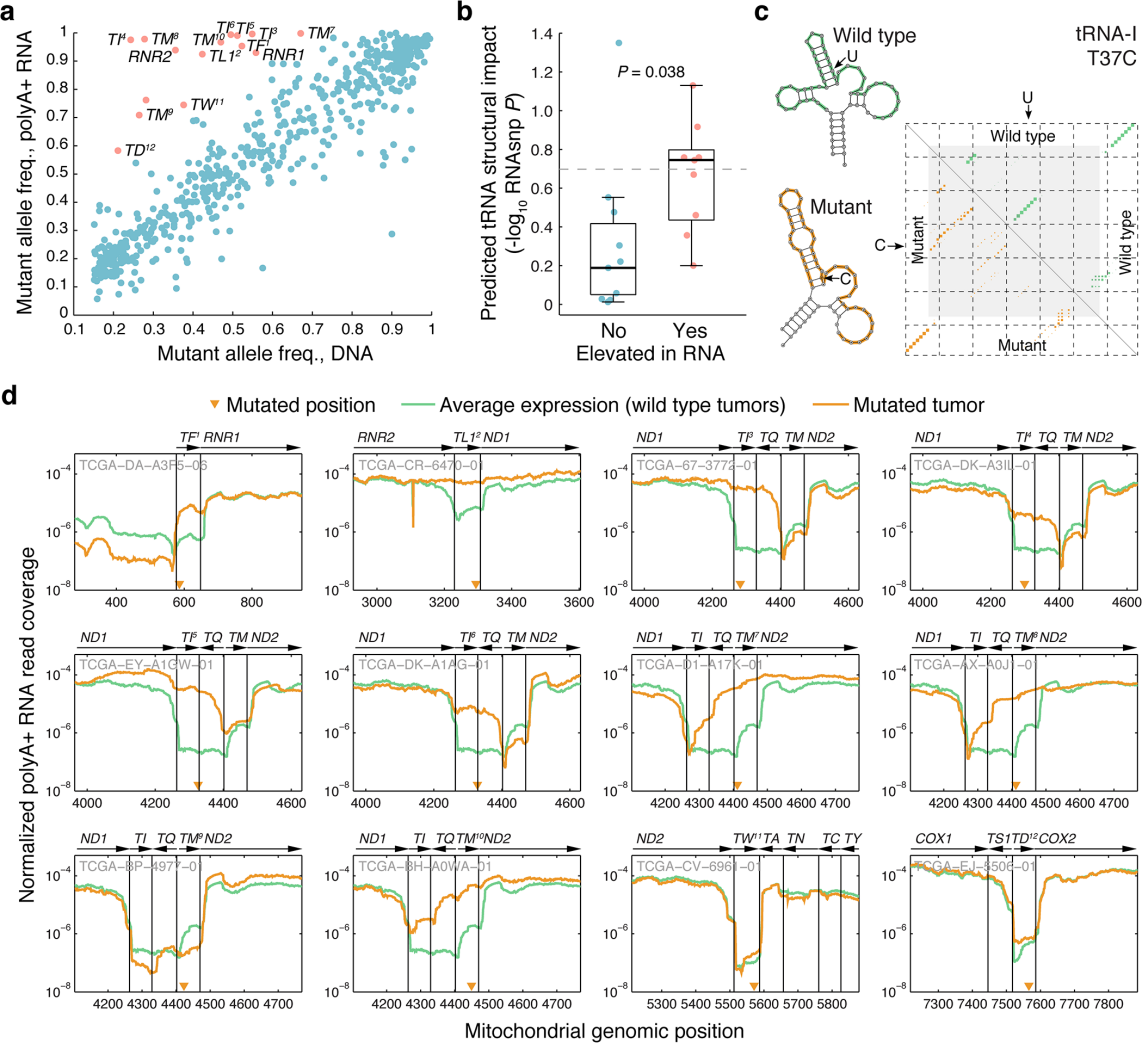
Comparison of allelic ratios in DNA and RNA reveals allelic imbalances consistent with impaired tRNA processing. (**a**) Scatter plot of allele frequencies (heteroplasmy levels) in DNA vs. RNA for all 616 mutations (*r* = 0.91). 15 mutations with marked accumulation in polyA+ RNA relative to DNA (frequency difference > 0.3) are indicated in red. 12 of these 15 mutations were in tRNAs regions (numbered 1–12 in superscript), indicative of impaired processing of the polyA+ precursor RNA to a mature polyA- tRNA. (**b**) tRNA mutations accumulated in polyA+ RNA (red in panel a) showed elevated predicted RNA structural impact, determined using the RNAsnp tool [35,36], compared to other tRNA mutations (*P* = 0.038, Wilcoxon rank sum test). The comparison was based on 9 and 9 inhibiting/non-inhibiting mutations (cases where the wild-type sequence failed to fold into a tRNA-like structure were excluded). The dotted line indicates an RNAsnp *P*-value (structural impact score) of 0.2 (**c**) Example RNAsnp result for a U to C mutation in position 37 of the mitochondrial isoleucine tRNA. The dot-plot shows the ensemble base-pair probabilities of the wild type (upper triangle) and mutant (lower triangle) sequences, with the altered local region indicated in gray. Wild type and mutant minimum free energy structures are shown (altered local region in color). (**d**) Normalized RNA read coverage, showing relative (per-tumor normalized) polyA+ expression levels across the mitochondrial genome in mutated tRNA regions for the 12 tRNA mutations indicated in panel a (each identifiable by a superscript number). Mutated cases (yellow) are compared to controls (green, median of all non-mutated cases). Gene strand orientation is indicated by arrows (right-facing: L-strand). Mutated positions are indicated by triangles. Samples IDs are shown in gray. tRNA genes are referred to as “TX”, where X = the single letter amino acid code.

Allelic ratios in general were highly consistent between DNA and polyA+ RNA (*r* = 0.91; **Fig 4A**). However, a subset of the mutant alleles showed clearly elevated frequencies in the polyA+ RNA pool (**Fig 4A**). Interestingly, of 15 mutations with marked elevation in RNA (frequency difference > 0.3; red dots in **Fig 4A**), 12 (80%) were found to localize to mt-tRNA regions, to be compared with 33/601 (5.5%) in the remaining set (*P* = 2.0e-12, Fisher’s exact test). Mature mt-tRNAs are not polyadenylated, in contrast to other mitochondrial RNA products and precursors, and thus should not appear in these sequencing libraries [9]. The elevated frequency in mRNA data for some mutant tRNA alleles is therefore most likely explained by a failure to process and clear the mutant molecules from the polyA+ precursor pool, causing accumulation relative to their wild type counterparts. We selected this set of mt-tRNA mutations with elevated frequency in RNA for further study, as they might provide insight into properties and requirements for mt-tRNA maturation.

It has previously been observed that some disease-associated mt-tRNA mutations that affect the secondary and tertiary structure can lead to the accumulation of processing intermediates [20,21]. We therefore compared the set of mt-tRNA mutations with elevated frequency in polyA+ RNA (**Fig 4A**, red dots) to remaining mt-tRNA mutations, with respect to the predicted structural impact (**Methods**). This revealed a significant difference in the predicted ability to disrupt the tRNA secondary structure, where most mutations with a strong predicted impact were in the category with elevated RNA allele frequency (**Fig 4B**, *P* = 0.038, Wilcoxon rank sum test; **S1 Table**). Similar results were obtained by instead applying a cutoff to the structural score followed by enrichment testing (**S2 Table**). **Fig 4C** shows an example of a T37C mutation in the tRNA^Ile^, where the substitution is predicted to alter the predicted cloverleaf fold to a non-canonical structure (see **S3 Fig** for more details). As an alternative approach, we used established mt-tRNA structures [37] to determine whether mutations localized to loop or stem regions, arguing that the latter would be more likely to affect structure. All of the 12 mutations with marked accumulation in RNA localized to stems, compared to 23/33 for remaining mt-tRNA mutations (*P* = 0.042, Fisher’s exact test). These results support that structure is a major determinant for proper mt-tRNA recognition and processing.

Next, we carefully examined expression levels (normalized polyA+ RNA-seq read coverage) in the areas surrounding the 12 tRNA mutations with marked allelic imbalance, comparing mutated to non-mutated tumors. In most cases, levels were elevated throughout the respective tRNAs, and often also in neighboring genes (discussed below), further supporting accumulation of polycistronic polyA+ precursors containing unprocessed tRNAs (**Fig 4D**). Increases compared to non-mutated control tumors were often dramatic (up to 100-fold), and the mildest variations were seen for the three mutations with the least DNA/RNA allelic imbalance (*tRNA* ^*Asp*^, *tRNA* ^*Trp*^, and *tRNA* ^*Met*^). Expression patterns for other tRNA mutations were in general more similar to controls (**S4 Fig**), and when processing was still clearly affected this was typically explained by a difference in DNA/RNA frequency close to the threshold used here (0.3) or, alternatively, a high mutant allele frequency in DNA, making it hard to detect further accumulation in RNA. Similar patterns of precursor accumulation have been observed when knocking down components of the mt-tRNA maturation pathway [18].

**Fig 5:**
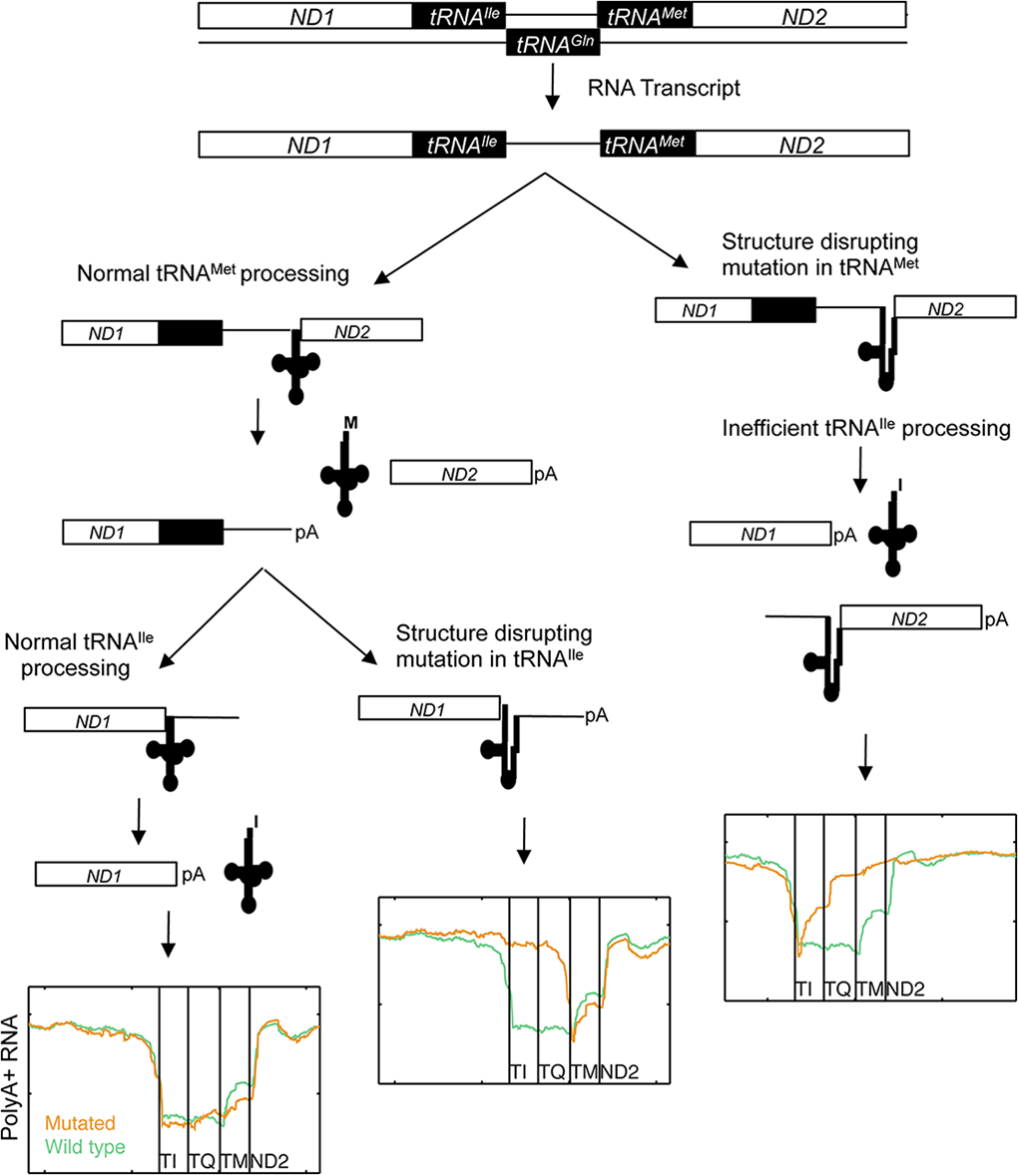
Proposed model of mt-tRNA processing in light of observed RNA species in human cancers. The normal processing cascade is depicted (left-hand side). The data in **Fig 4** suggests that proper folding of the pre-tRNAs is important for RNAse P/Z processing. Structure-disrupting mutations in *tRNA* ^*Ile*^ allow for normal processing of the *tRNA* ^*Met*^, but leave polyA+ processing-intermediate products with mutation-bearing *tRNA* ^*Ile*^ and the antisense-*tRNA* ^*Gln*^ sequences on the 3’ end of *ND1* (middle). This implies that the antisense *tRNA* ^*Gln*^ is not a substrate for tRNA processing endonucleases. Structure-disrupting mutations in the *tRNA* ^*Met*^ gene lead to the accumulation of intermediates in which the antisense *tRNA* ^*Gln*^ and mutation-bearing tRNA^*Met*^ sequences remain attached to the 5’ end of the *ND2* gene (right-hand side). Processing of the wild type *tRNA* ^*Ile*^ still occurs but at reduced efficacy, consistent with a model whereby processing of a multi-tRNA cluster occurs preferably in the 3’ to 5’ direction.

Finally we investigated how impaired processing of specific mt-tRNAs affected the maturation of neighboring genes, since cleavage at mt-tRNA boundaries is key to converting the mitochondrial large polycistronic RNAs into smaller gene products (**Fig 4D**). Eight mutations occurred in the *ND1-tRNA* ^*Ile*^ *-*antisense *tRNA* ^*Gln*^ *-tRNA* ^*Met*^ *-ND2* cluster of tRNAs. Four observed mutations in tRNA^Ile^ all led to accumulation of intermediates where both *tRNA* ^*Ile*^ and antisense *tRNA* ^*Gln*^ remained at the 3´ end of *ND1* while processing of the 3´-encoded *tRNA* ^*Met*^ was unaffected. This supports that many mt-tRNA mutations may have a functional impact on neighboring coding genes, since polyadenylation at the correct site can be crucial for mRNA function [38]. Interestingly, mutations in the acceptor stem of *tRNA* ^*Met*^ in two cases instead lead to the accumulation of intermediates with anti-sense *tRNA* ^*Gln*^ and *tRNA* ^*Met*^ remaining 5´ of *ND2*. Processing of the 5´-encoded *tRNA* ^*Ile*^ was also moderately impaired in these cases. This implies that *tRNA* ^*Ile*^ can be processed from the mutant RNA species, but less efficiently. Our observations are consistent with a model whereby 3´ to 5´ removal of the tRNAs within a multi-tRNA cluster is more efficient than 5′ to 3′ or uncoordinated processing order [39] (**Fig 5**).

## Discussion

Sequencing data from TCGA here enabled us to identify and characterize somatic mutations in mtDNA across a diverse landscape of human cancers. In our study, we have considered dual evidence from both DNA and RNA in a comprehensive patient cohort, for the first time enabling a systematic analysis of mitochondrial DNA/RNA allelic imbalances across a large number of samples. We additionally provide confirmatory results regarding purifying selection in coding regions as well as the basic mutational signature of somatic mtDNA changes in tumors [8].

In agreement with [8], we believe that the dramatic mutational strand bias likely relates to the fact that the mode of replication differs between the two mtDNA strands [32]. According to the strand displacement model for mtDNA replication, H-strand replication requires the TWINKLE DNA helicase to unwind the double-stranded mtDNA genome, whereas L-strand synthesis is performed using the displaced, single-stranded parental H-strand as template. The nature of the template in the two reactions (dsDNA vs. ssDNA) will most likely affect both rate and processivity of mtDNA replication, which in turn can cause unequal fidelity of L-strand and H-strand synthesis. Alternatively, the strand bias could be due to hydrolytic deamination of C to U, which is more likely to occur on the single-stranded parental H-strand. Arguing against this model is the absence of a gradient coinciding with the duration of single strand exposure during mtDNA replication, i.e. higher levels of C>T mutations closer to the control region and a gradual drop towards the origin of L-strand replication [40].

The dual DNA/RNA design of this study allowed us to investigate how somatic mtDNA changes were reflected at the transcriptomic level. Allelic ratios overall were concordant between DNA and RNA, despite the presence of many frame-shifting or stop-codon inducing mutations in our compendium. Importantly, this argues against the presence of potent mutation-dependent mRNA surveillance mechanisms akin to nonsense-mediated decay in mitochondria. Conversely, there was no evidence for mutation-induced increases in the levels of individual mRNA transcripts. However, a small number of mutations, mostly in tRNA genes, showed a curious excess of the mutated allele in polyA+ RNA-seq compared to DNA, indicative of impaired processing from a polyA+ precursor into a polyA- mature tRNA. This phenomenon helped to confirm hypotheses regarding processing of mt-tRNAs, including a role for the tRNA fold in recognition by processing endonucleases, as these mutations were associated with impaired tRNA structure. Mutations in mt-tRNAs underlie a range of conditions, including neural, gastrointestinal and muscular disorders [19]. While the underlying mechanisms are variable and poorly understood, is has been noted that pathological variants often localize to stem regions of the cloverleaf-like mt-tRNA structure, suggestive of a structural impact [19]. Our data suggests that mutations affecting tRNA folding may impair maturation of not only the affected tRNA, but also neighboring gene transcripts. This could be an important part in the pathology of many disease-associated mt-tRNAs variants and may explain why mutations in the same tRNA may present radically different disease phenotypes.

Of interest is the behavior of the antisense *tRNA* ^*Gln*^ sequence, clustered between the *tRNA* ^*Ile*^ and *tRNA* ^*Met*^ genes. Mutations in either of the flanking tRNAs lead to accumulation of polyA+ intermediates for this element, suggesting that this antisense tRNA is not able to adopt a confirmation identified by the processing enzymes, and instead behaves simply as an RNA spacer. More work is needed to determine whether this can be generalized to other analogous mtDNA regions. In the same cluster, we found that processing of *tRNA* ^*Met*^ appeared unaffected by mutations in *tRNA* ^*Ile*^. Interestingly, the converse was not true, as processing of *tRNA* ^*Ile*^ was partially affected by mutations in *tRNA* ^*Met*^, most evidently at the 3´ side of *tRNA* ^*Ile*^ (**Fig 4D**). It has previously been reported in the fruitfly *Drosophila melanogaster*, that mitochondrial tRNA processing appears to occur in a 3´ to 5´ manner [39,41]. Our data supports model whereby processing of preferably occurs in the 3´ to 5´ direction, but where 5´ encoded tRNAs may still be identified and liberated at lower efficacy.

The nuclear genome has in recent years been extensively surveyed with respect to somatic mutations in tumors. A massive body of available sequencing data pertaining to the mitochondrial genome and transcriptome, generated as a byproduct of these surveys, remains curiously understudied. Controlled manipulation of mtDNA is difficult, as standard genetic tools are not applicable to mitochondria. In the present work, we have demonstrated how a large compendium of genetic perturbations, derived from human tumor sequencing data, can provide basic insight into mitochondrial function.

## Methods

### Mutation calling in DNA and RNA

WGS data in bam format (Hg19 assembly) from 527 tumor/normal pairs sequencing by the TCGA were obtained from the cgHub repository (**S1 Dataset**). This set included non-embargoed tumors with a minimum file size of 75 GB, where matching RNA-seq data was available. Colon and rectal carcinoma samples (COAD/READ) were merged into one cancer type (CRC). Somatic mutations were called with VarScan2^7^ essentially as described previously [42]: we used samtools (command *mpileup* with default settings and additional options-*q1* and–*B*) and VarScan in somatic mode with the additional option–*strand-filter 1*). We required a minimum variant frequency of 15%, *P*-value below 0.001 was required, and a variant frequency below 0.5% in the normal. WGS mutation calling was performed at the UPPMAX HPC Center (Uppsala, Sweden).

The default alignment protocol employed by the TCGA for RNA-seq is not optimal for mutation calling, and indels in particular will not be properly called. RNA-seq data was therefore realigned to the chrM_rRCS reference using bwa [43] (options–q 5 and-l 32). This was followed by GATK’s IndelRealigner [44] (default parameters) to correct misaligned indels. Lastly, somatic mutations were detected as described above but using the parameters–*E* and–*q25* parameters for mpileup and—*min-var-freq 0*.*02* for VarScan.

### Expression coverage plots

Expression level plots across the mitochondrial genome (polyA+ RNA-seq read coverage data) were determined using the bedtools[45] *genomecov* command, based on realigned data as described above. The coverage data was normalized by dividing by the total number of reads aligned to chrM in each sample.

### Assessing structural impact of tRNA mutations

For mutations in mitochondrial tRNAs, we used RNAsnp (v1.1) [35,36] to determine whether there was a likely impact on the structure. Briefly, this tool will fold the wild-type and the mutated RNA, and determine a score (and *P*-value) that reflects the extent to which the secondary structures differ. For the *P*-value calculation, RNAsnp uses one of several pre-computed background distribution of scores available for different sequence lengths. For our analysis, we chose the lowest sequence length (200nts) available using the parameter '-w 100' along with 'Mode 1' (Euclidean distance measure). However, to avoid any biases in the *P*-value calculation due to the differences in the sequence length match, we also generated new background scores by using random sequences of length similar to the tRNA sequences by following the procedure described in [35], except that the random sequences were generated by di-nucleotide preserved shuffling of tRNA sequences. The *P*-values computed using this new background scores correlate well with the default *P*-values from pre-computed background scores (**S5 Fig**). tRNAs where the native structure did not fold properly (based on comparison of predicted base pairs of wild-type ensemble structure with the reference secondary structure from Mamit-tRNA [46] and tRNAdb databases [37]) were excluded from analysis, which resulted in 18 cases of tRNA mutations that could be further analyzed (see **S1 Table** and **S3 Fig**). RNAsnp *P*-values (using the new background distribution) for mutations that showed allelic imbalances, indicative of processing defects (allelic ratio difference >0.3; red in **Fig 4A**), were finally compared to those of mutations that showed expected allelic ratios.

## Supporting Information

S1 Fig Histogram of read coverage in WGS for all identified somatic mutations. The average read coverage for the 616 somatic mutations was 5342 in the tumors and 3751 in the normals (minimum 279 and 118, respectively).
(PDF)

S2 Fig Position-specific mutational signatures throughout the mitochondrial genome. Blab. The circular mitochondrial genome was divided into 50 segments of 331 nt. The mutational signature for each segment was determined similarly to main **Fig 1**, but with overrepresented mutational events indicated using a color code instead of bar graphs.
(PDF)

S3 Fig Detailed graphical output of RNAsnp predictions. **(a**) Base-pair probabilities: The upper and lower triangle of the matrix represents the ensemble base pair probabilities of wild-type and mutant sequences, respectively. In case of wild-type, the base pairs that match with the reference tRNA secondary structure [37,46] are highlighted in light green color. The local region detected with maximum base pair changes is highlighted in gray background. (**b**) and (**c**) Minimum free energy structures of the wild-type and mutant sequences, respectively. The region highlighted in different colour corresponds to the local region as detected in the above base pairing probability matrix (**a**).
(PDF)

S4 Fig Additional RNA read coverage plots. The plots show relative (per-tumor normalized) polyA+ expression levels across the mitochondrial genome in mutated regions for additional mutations not included in Fig 4A and 4D. All additional mutations in with allele frequency difference > 0.3 or < -0.3 are shown, as well as all tRNA mutations in the -0.3…0.3 range. All Mutated cases (red) are compared to controls (blue, median of all non-mutated cases). Mutated positions are indicated by triangles. VAF_DNA_, variant allele frequency in DNA; VAF_RNA_, variant allele frequency in RNA.
(PDF)

S5 Fig Comparison of RNAsnp *P*-values computed using default and tRNA-adapted background scores. (PDF)

S1 Appendix Comparison of observed synonymous:nonsynonymous ratios to an expected null model. (PDF)

S1 Dataset Information regarding sequencing libraries included in the study. (XLSX)

S2 Dataset Detailed catalogue of somatic mutations identified across the 527 tumors considered in the study, including allele frequencies in DNA and RNA. The table includes 644 mutations identified using WGS, of which 616 where confirmed in RNA-seq.
(XLSX)

S1 Table Somatic mutations in mt-tRNAs. DNA and RNA allele frequencies are indicated, as well as predicted structural impact determined using RNAsnp. The column “Native structure predicted” represents whether the wild-type secondary structure predicted by RNAsnp match the reference tRNA secondary structure (clover-leaf structure) [37,46] (see **S3 Fig** for more details).
(PDF)

S2 Table Enrichment analysis of structure-disruptive mutations in the processing defect group compared to no processing defect group, under different RNAsnp *P*-value cut-offs. (PDF)
