# Supplementary figures and images for: Simultaneous DNA and RNA Mapping of Somatic Mitochondrial Mutations across Diverse Human Cancers
Source: PLoS Genet. 2015 Jun 30;11(6):e1005333. doi: 10.1371/journal.pgen.1005333 (PMC4488357; doi:10.1371/journal.pgen.1005333)

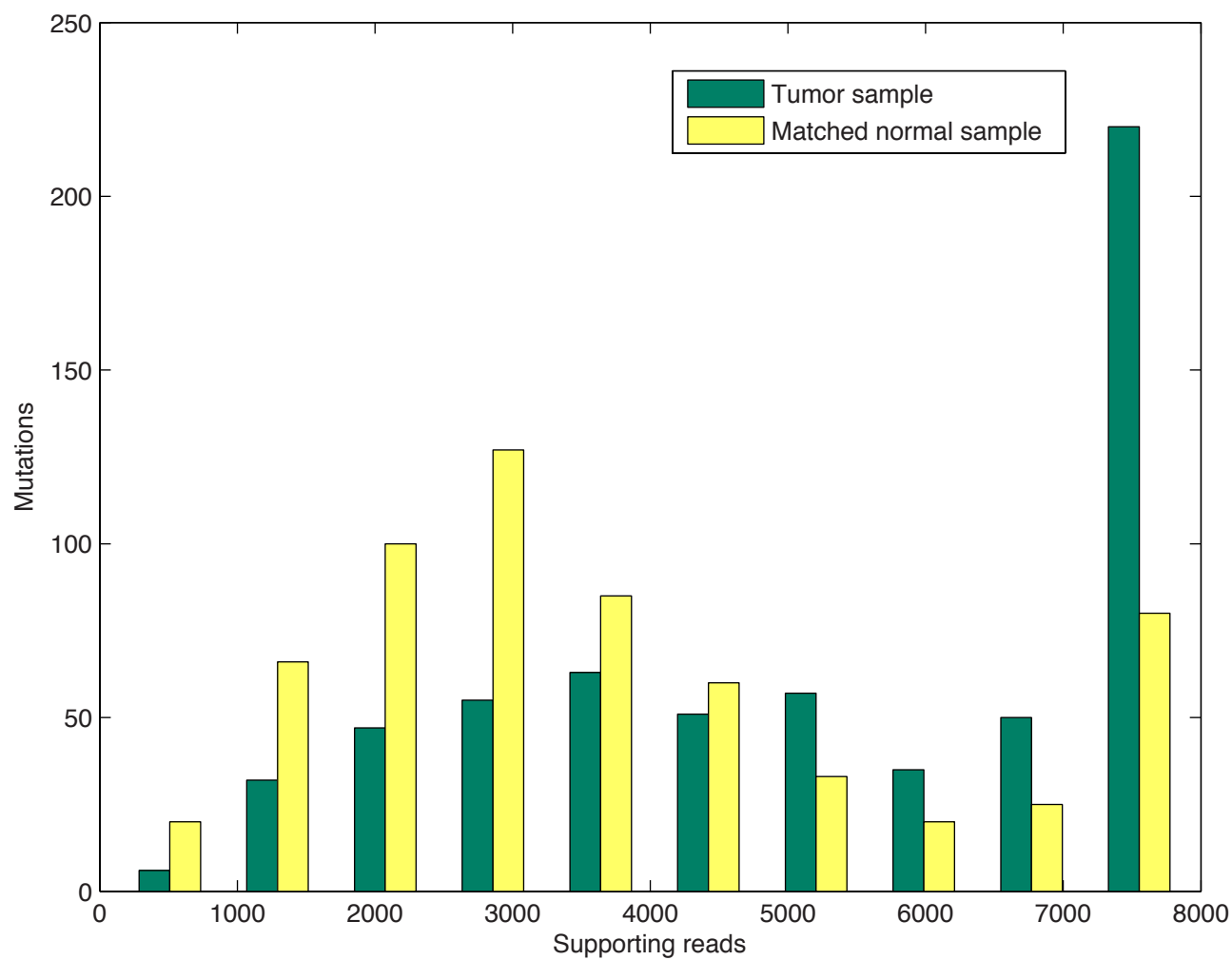

Supplement: S1 Fig — The average read coverage for the 616 somatic mutations was 5342 in the tumors and 3751 in the normals (minimum 279 and 118, respectively). (PDF) [file pgen.1005333.s001.pdf]

# Mutational signature

C>A C>G C>T T>A T>C T>G

L H L H L H L H L H L H

| | | | | | | | | | | |

| | | | | | | | | | | |

| | | | | | | | | | | |

| | | | | | | | | | | |

| | | | | | | | | | | |

| | | | | | | | | | | |

| | | | | | | | | | | |

| | | | | | | | | | | |

| | | | | | | | | | | |

| | | | | | | | | | | |

| | | | | | | | | | | |

| | | | | | | | | | | |

| | | | | | | | | | | |

| | | | | | | | | | | |

| | | | | | | | | | | |

| | | | | | | | | | | |

| | | | | | | | | | | |

| | | | | | | | | | | |

| | | | | | | | | | | |

| | | | | | | | | | | |

## Enrichment

0 2 4 6 8 10

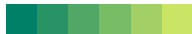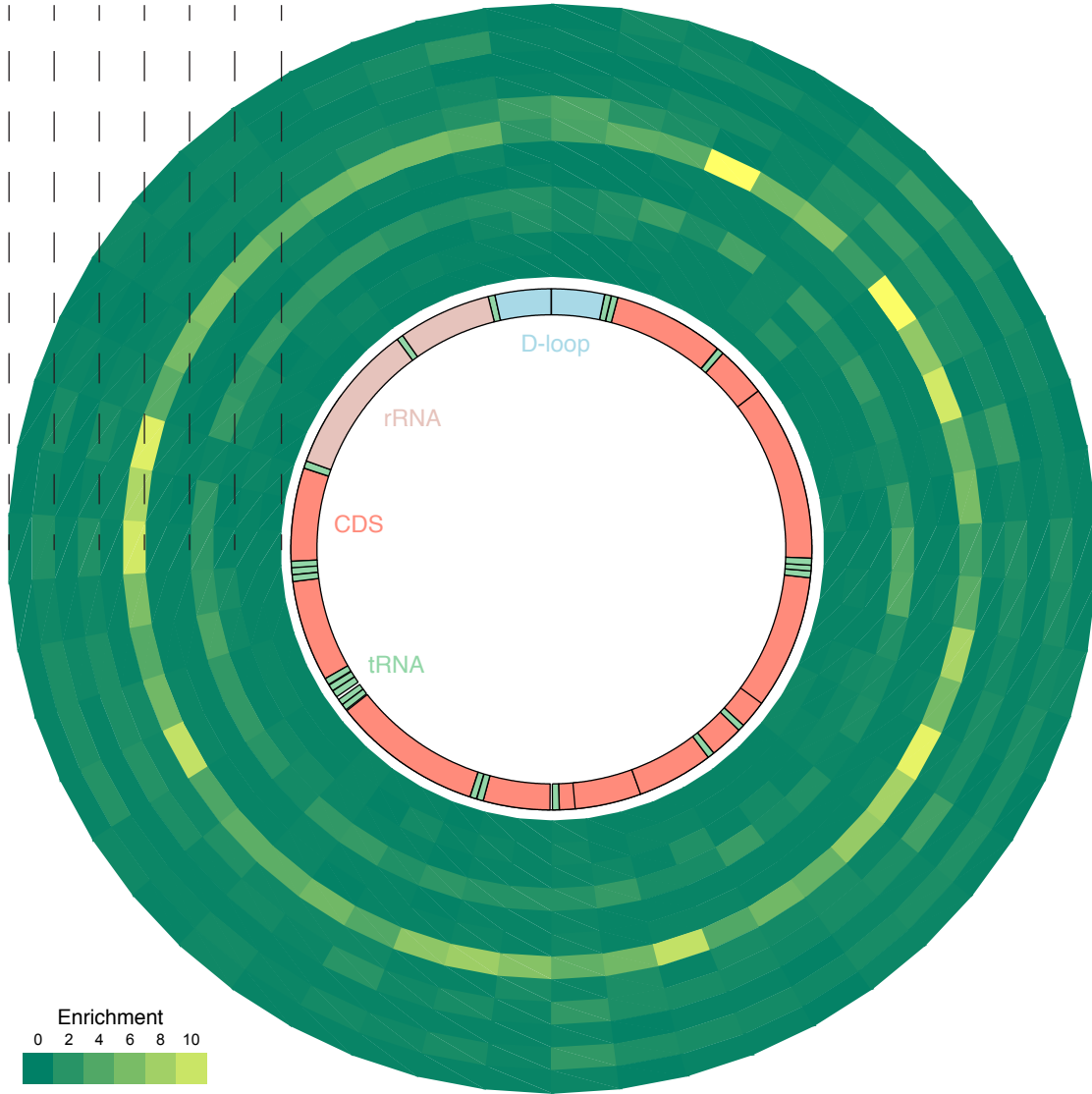

Supplement: S2 Fig — Blab. The circular mitochondrial genome was divided into 50 segments of 331 nt. The mutational signature for each segment was determined similarly to main Fig 1, but with overrepresented mutational events indicated using a color code instead of bar graphs. (PDF) [file pgen.1005333.s002.pdf]

RNAseq prediction for all 45 mutations

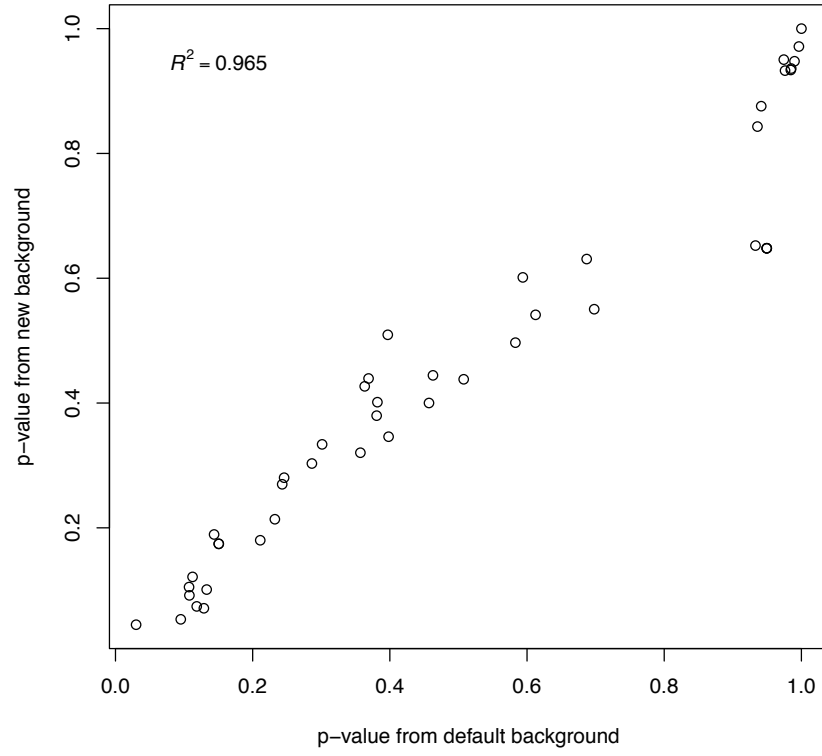

Supplement: S5 Fig — (PDF) [file pgen.1005333.s005.pdf]
