# Supplementary material for: Simultaneous DNA and RNA Mapping of Somatic Mitochondrial Mutations across Diverse Human Cancers
Source: PLoS Genet. 2015 Jun 30;11(6):e1005333. doi: 10.1371/journal.pgen.1005333 (PMC4488357; doi:10.1371/journal.pgen.1005333)
Supplement: S3 Fig — (a) Base-pair probabilities: The upper and lower triangle of the matrix represents the ensemble base pair probabilities of wild-type and mutant sequences, respectively. In case of wild-type, the base pairs that match with the reference tRNA secondary structure [37,46] are highlighted in light green color. The local region detected with maximum base pair changes is highlighted in gray background. (b) and (c) Minimum free energy structures of the wild-type and mutant sequences, respectively. The region highlighted in different colour corresponds to the local region as detected in the above base pairing probability matrix (a). (PDF) [file pgen.1005333.s003.pdf]

Ref.	SampleID	chrPos	tRNA	Strand	mut	RNAsnp P-value	nsp
1	TCGA-A2-A04T-01	5881	TY	-	C11U	0.0447	yes

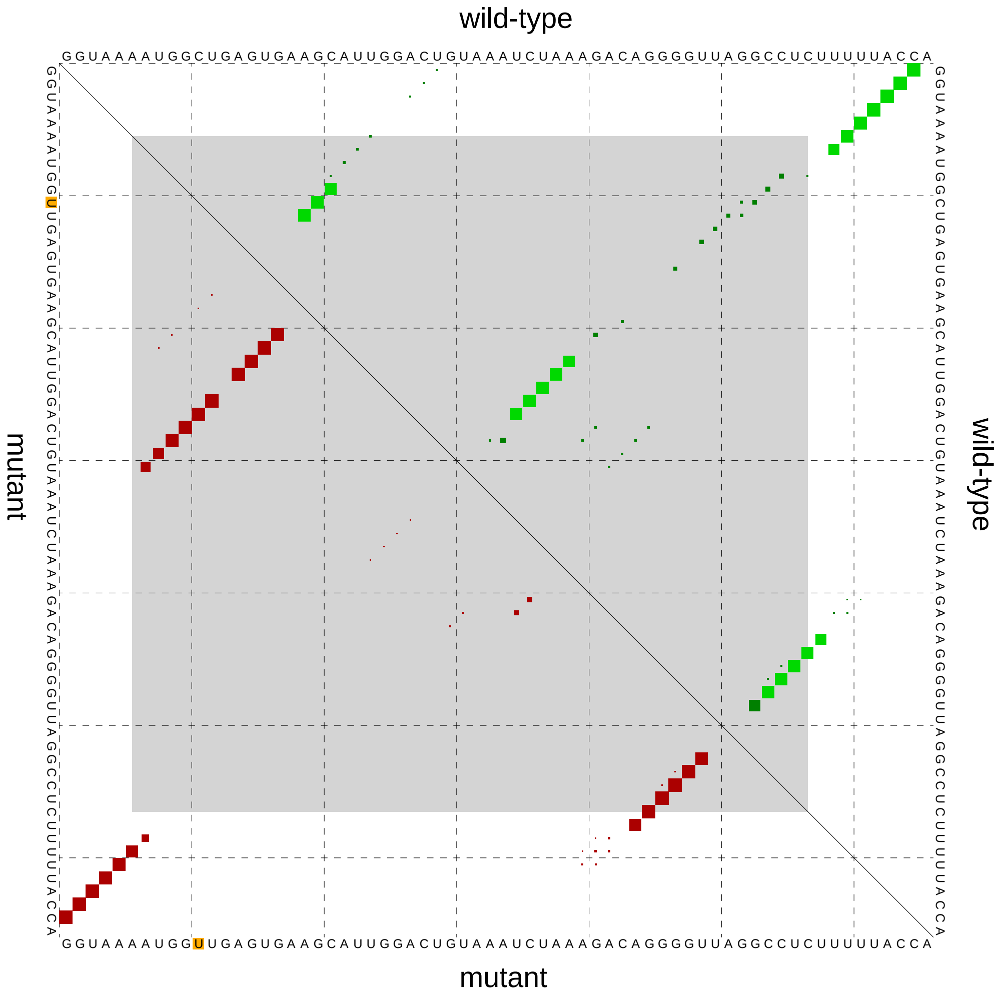

(a) Base-pair probabilities

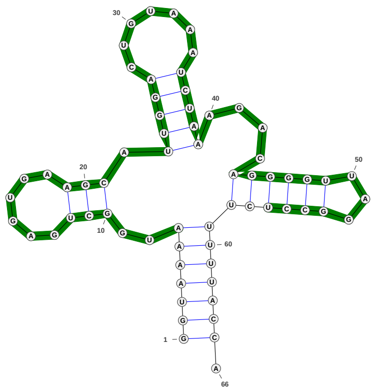

(b) wild-type

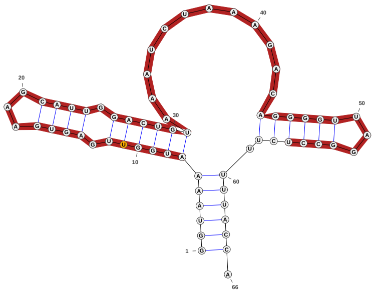

(c) mutant

Ref.	SampleID	chrPos	tRNA	Strand	mut	RNAsnp P-value	nsp
2	TCGA-DK-A1AG-01	4328	TI	+	U66C	0.0740	yes

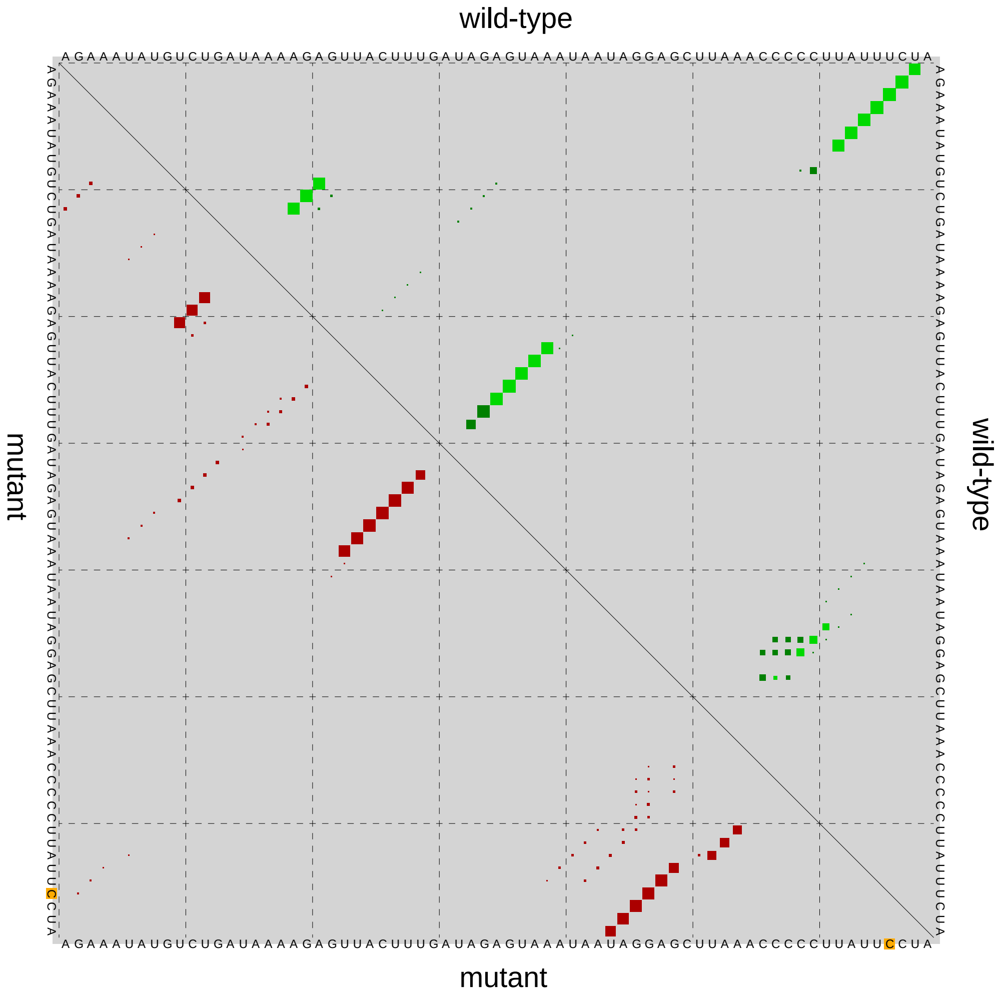

(a) Base-pair probabilities

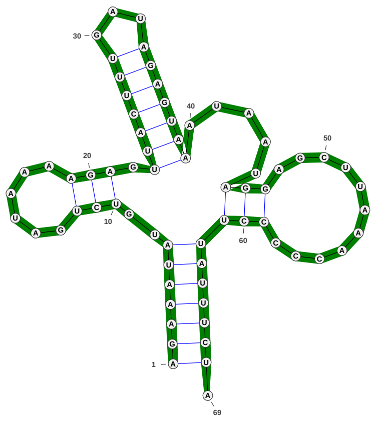

(b) wild-type

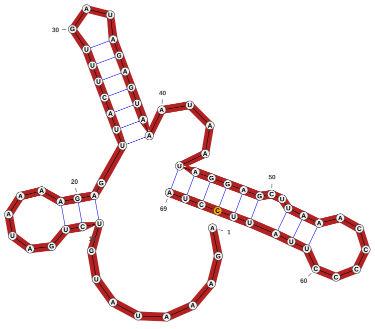

(c) mutant

Ref.	SampleID	chrPos	tRNA	Strand	mut	RNAsnp P-value	nsp
3	TCGA-DK-A3IL-01	4299	TI	+	U37C	0.1213	yes

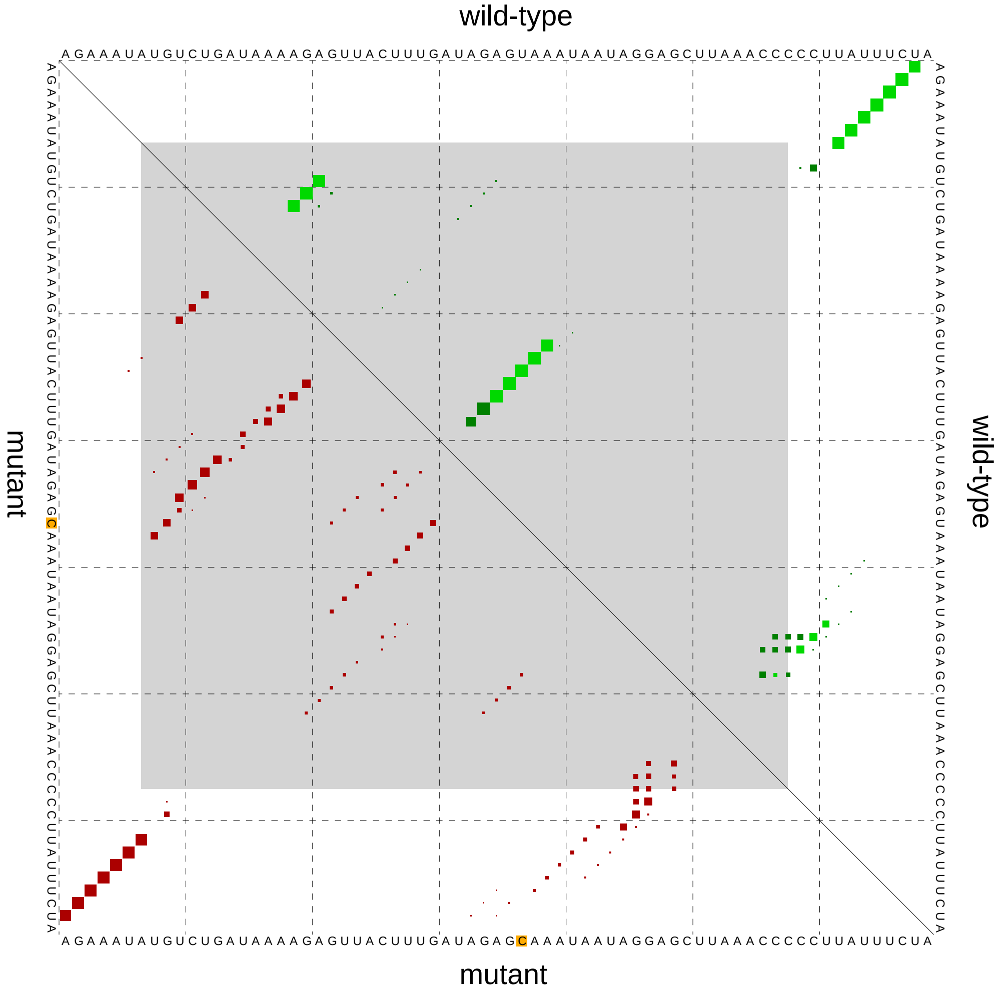

(a) Base-pair probabilities

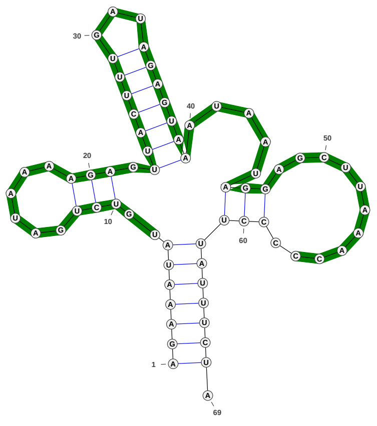

(b) wild-type

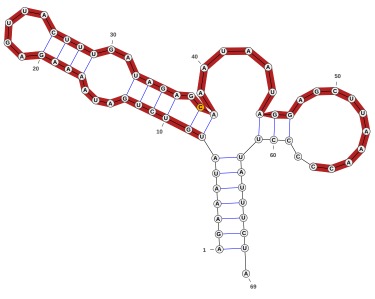

(c) mutant

Ref.	SampleID	chrPos	tRNA	Strand	mut	RNAsnp P-value	nsp
4	TCGA-D1-A17K-01	4412	TM	+	G11A	0.1743	yes

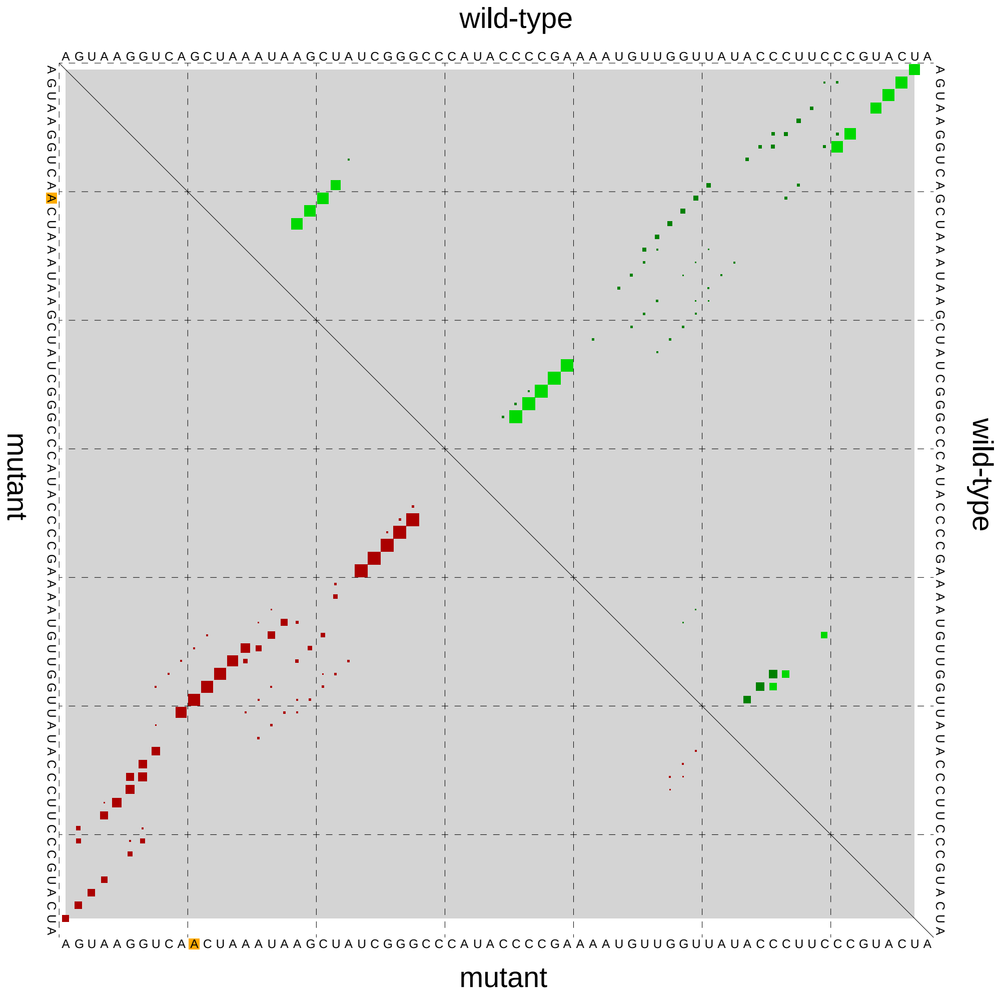

(a) Base-pair probabilities

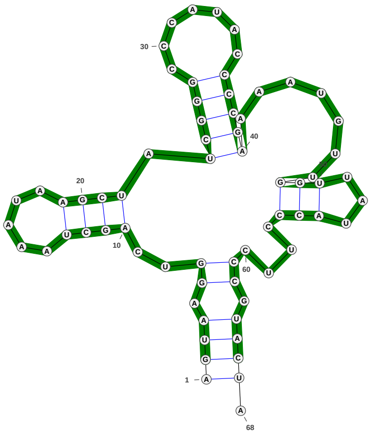

(b) wild-type

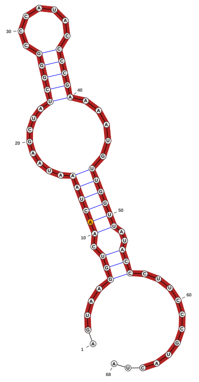

(c) mutant

Ref.	SampleID	chrPos	tRNA	Strand	mut	RNAsnp P-value	nsp
5	TCGA-AX-A0J1-01	4412	TM	+	G11A	0.1743	yes

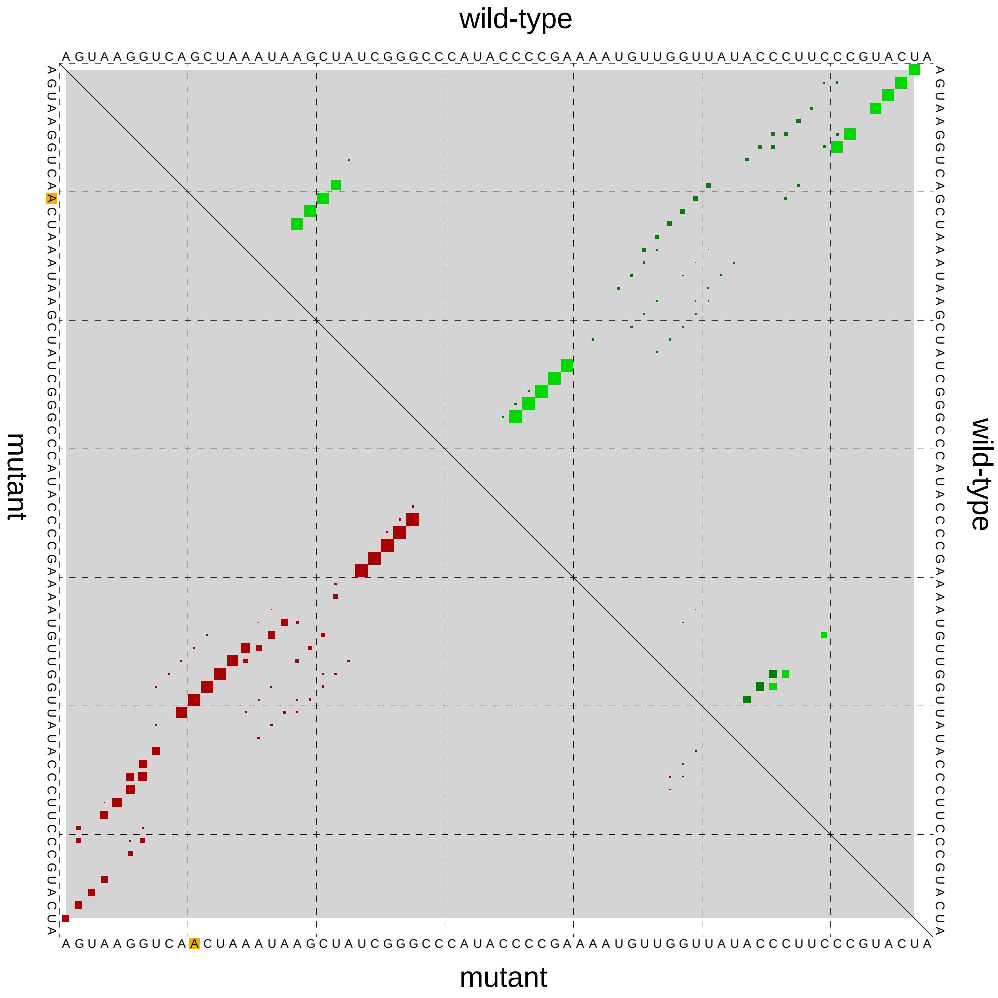

(a) Base-pair probabilities

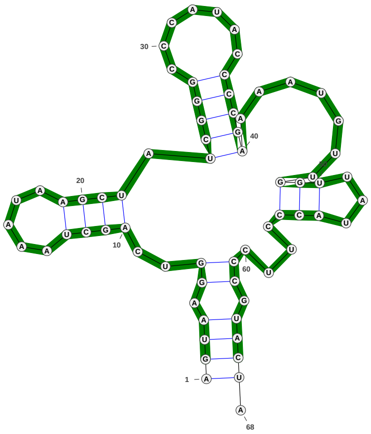

(b) wild-type

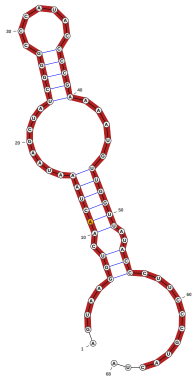

(c) mutant

Ref.	SampleID	chrPos	tRNA	Strand	mut	RNAsnp P-value	nsp
6	TCGA-EY-A1GW-01	4326	TI	+	U64C	0.1800	yes

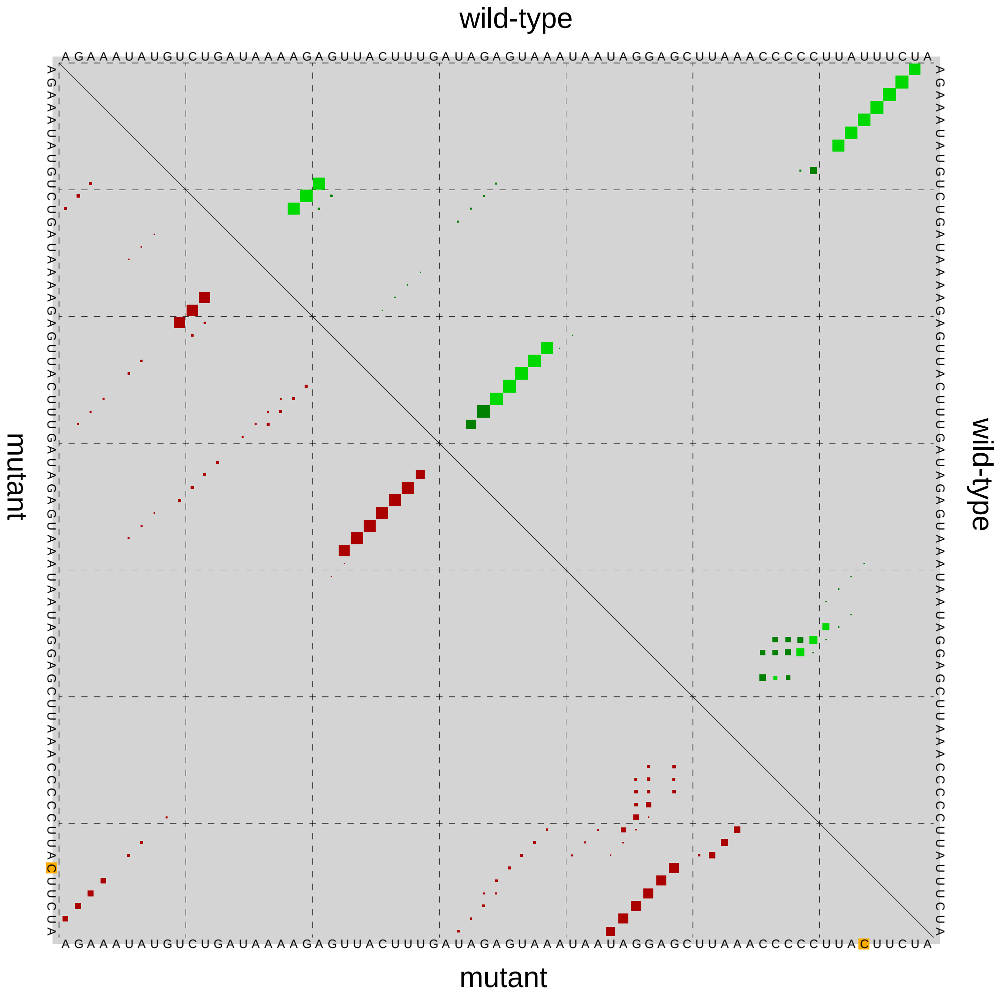

(a) Base-pair probabilities

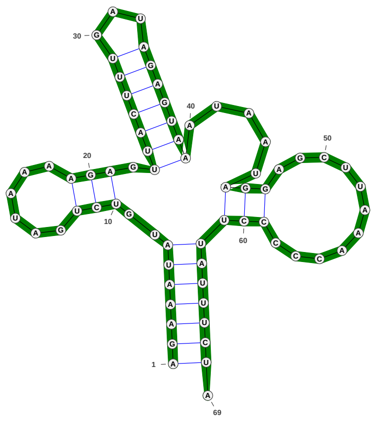

(b) wild-type

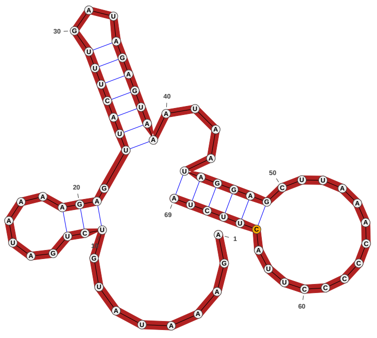

(c) mutant

Ref.	SampleID	chrPos	tRNA	Strand	mut	RNAsnp P-value	nsp
7	TCGA-BP-4977-01	4423	TM	+	U22C	0.2137	yes

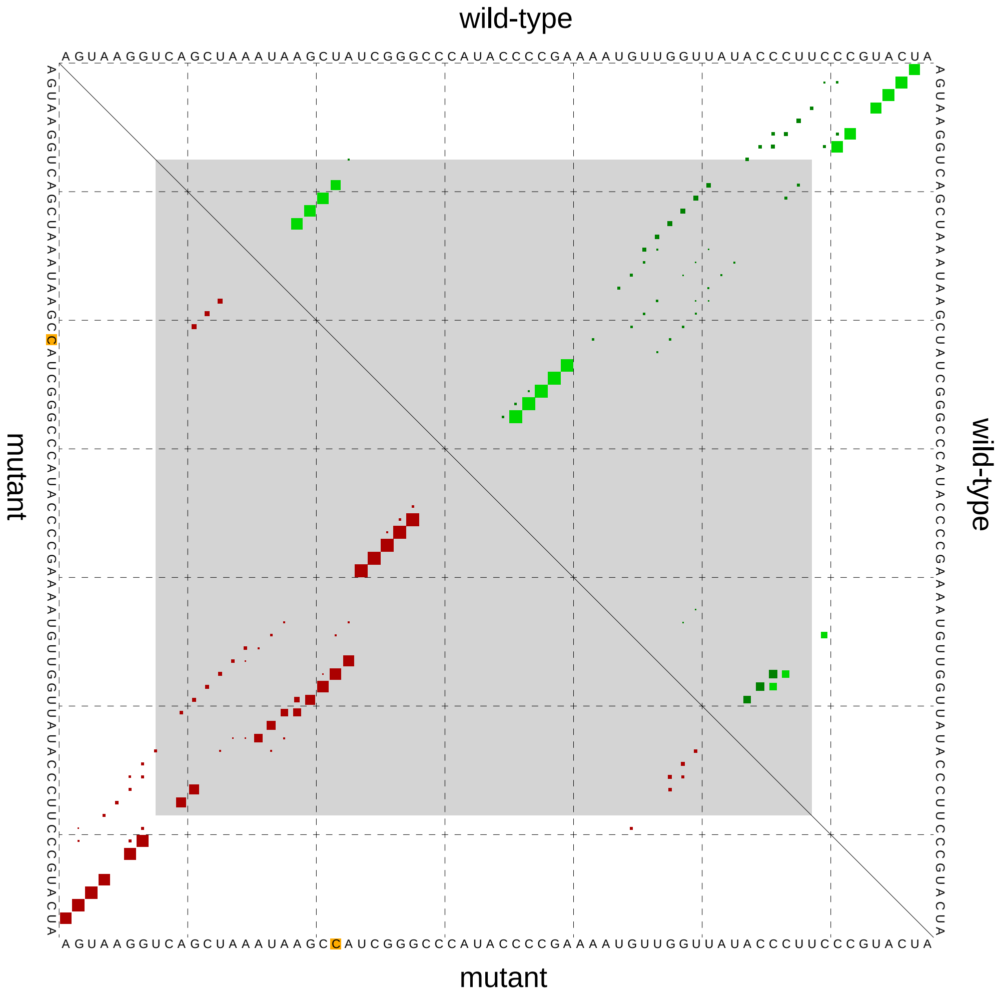

(a) Base-pair probabilities

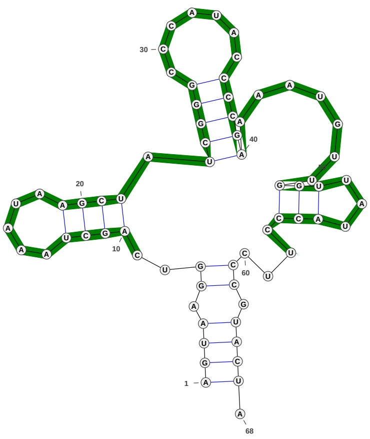

(b) wild-type

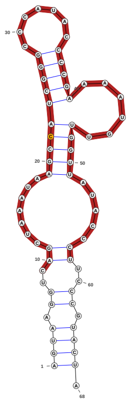

(c) mutant

Ref.	SampleID	chrPos	tRNA	Strand	mut	RNAsnp P-value	nsp
8	TCGA-AP-A05A-01	7453	TS1	-	C62U	0.2803	yes

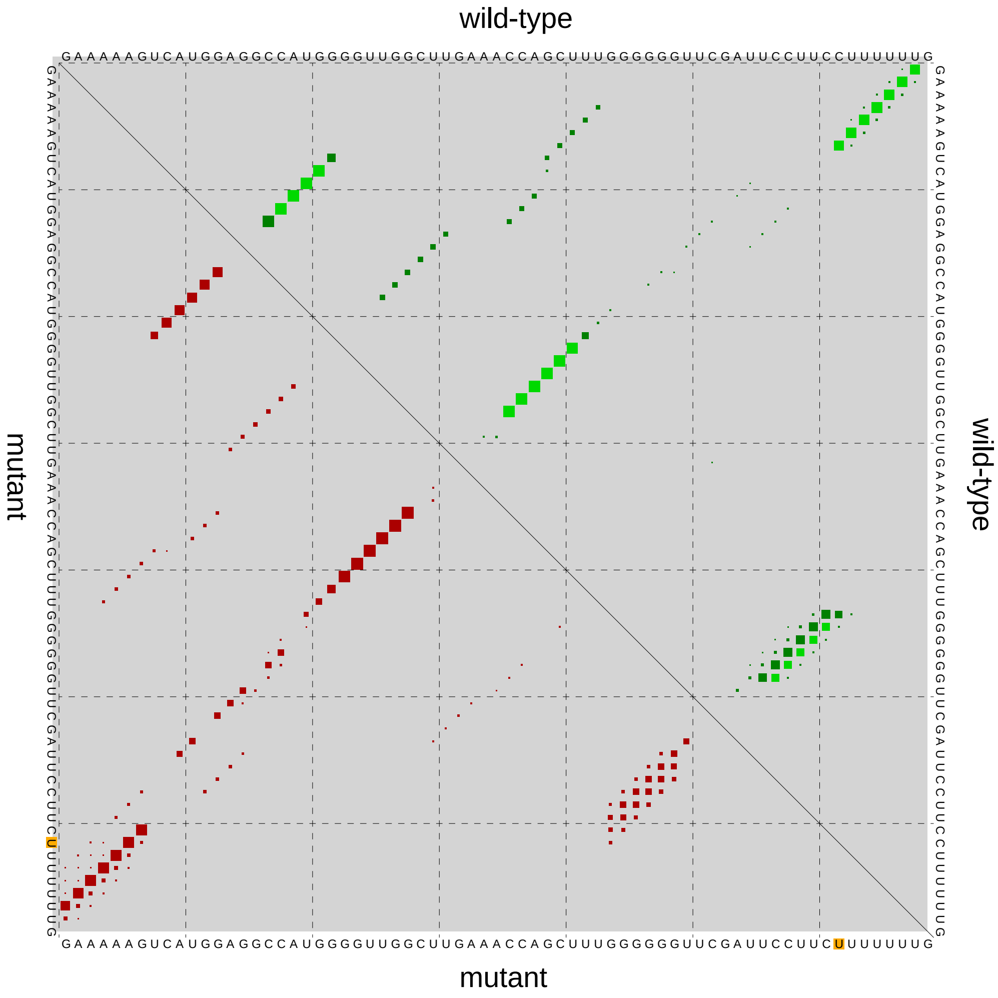

(a) Base-pair probabilities

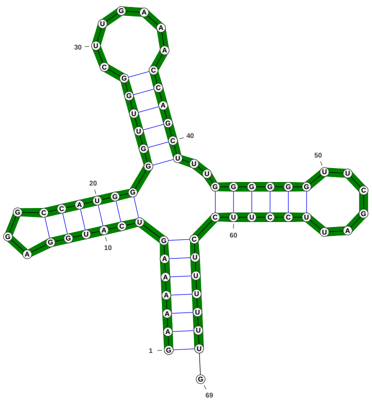

(b) wild-type

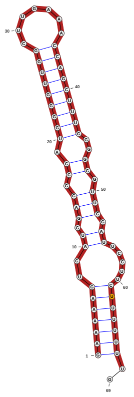

(c) mutant

Ref.	SampleID	chrPos	tRNA	Strand	mut	RNAsnp P-value	nsp
9	TCGA-DJ-A2Q2-01	4316	TI	+	A54G	0.3337	yes

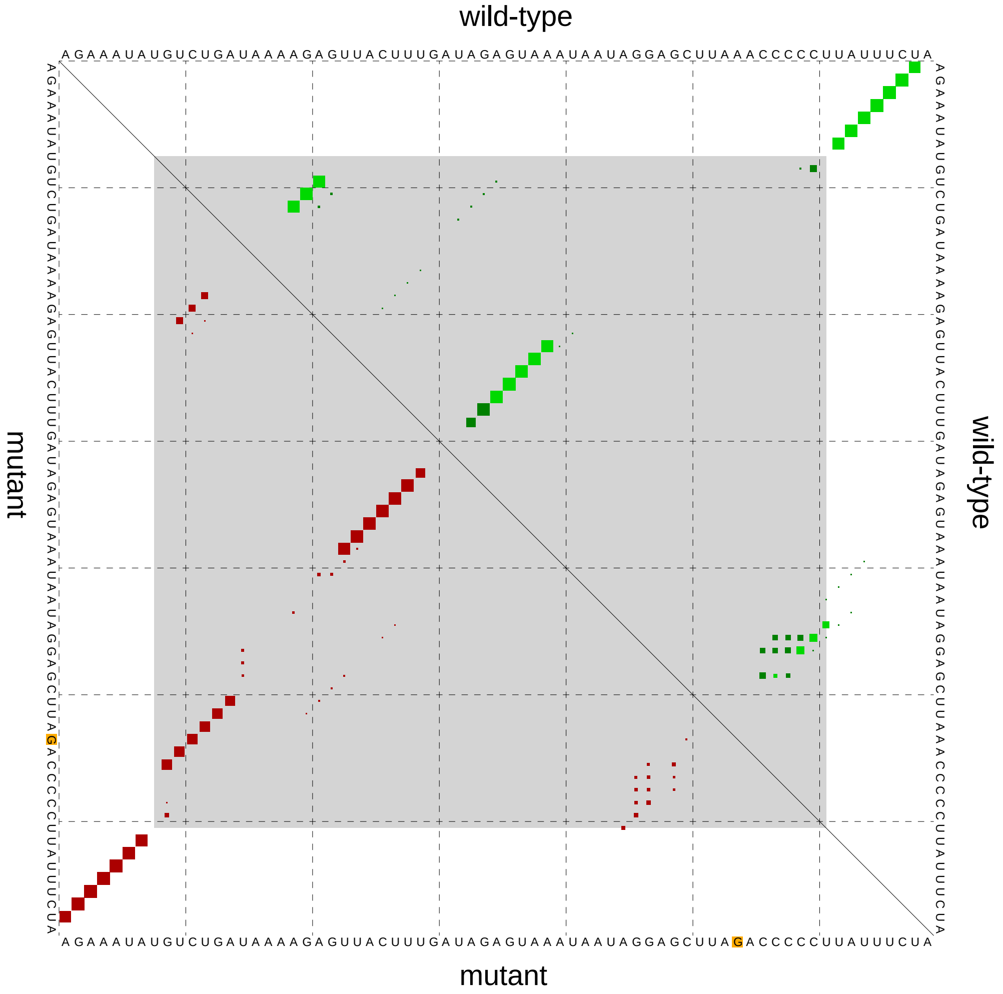

(a) Base-pair probabilities

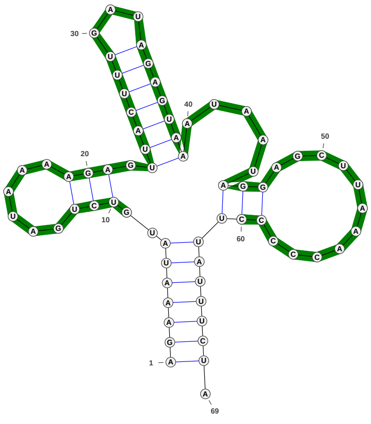

(b) wild-type

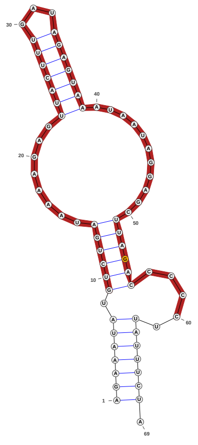

(c) mutant

Ref.	SampleID	chrPos	tRNA	Strand	mut	RNAsnp P-value	nsp
10	TCGA-BH-A0WA-01	4449	TM	+	G48A	0.3460	yes

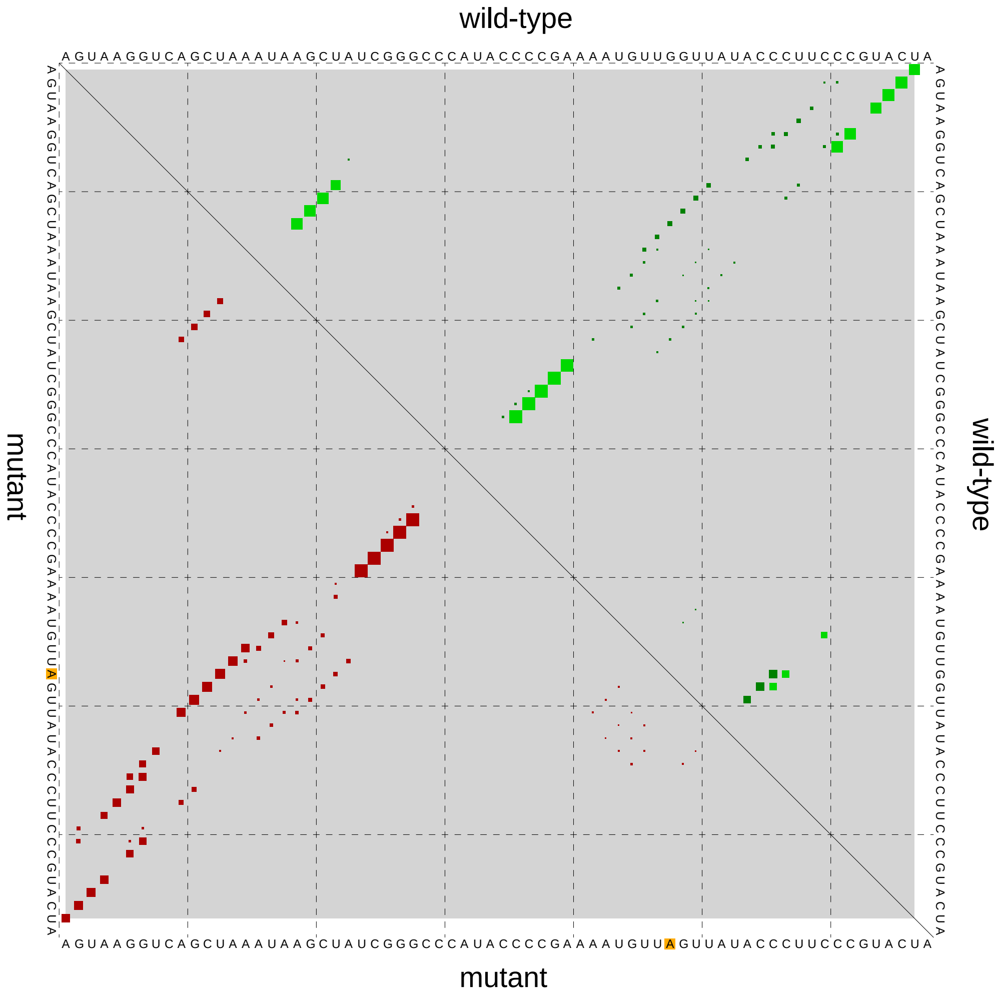

(a) Base-pair probabilities

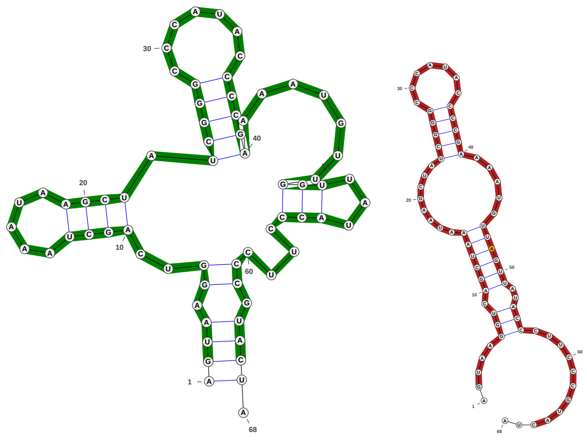

(b) wild-type (c) mutant

Ref.	SampleID	chrPos	tRNA	Strand	mut	RNAsnp P-value	nsp
12	TCGA-67-3772-01	4282	TI	+	G20A	0.4393	yes

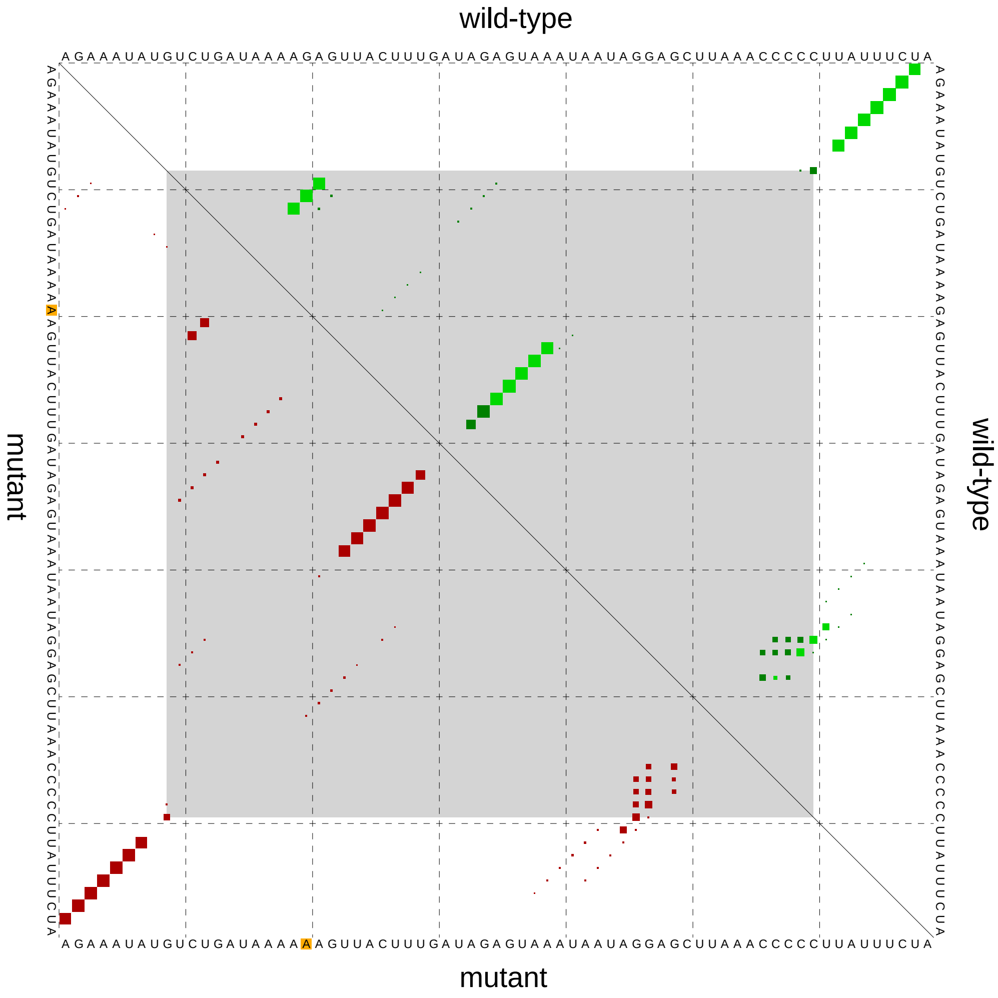

(a) Base-pair probabilities

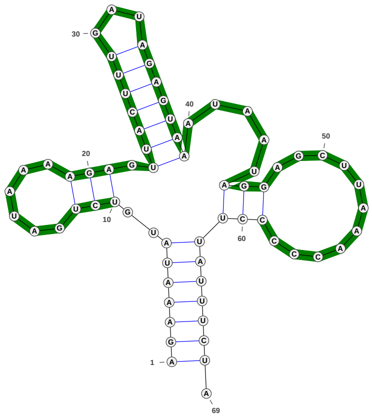

(b) wild-type

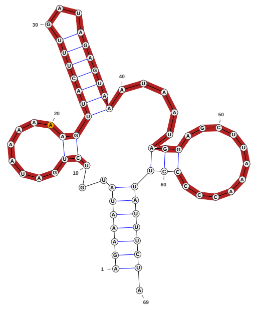

(c) mutant

Ref.	SampleID	chrPos	tRNA	Strand	mut	RNAsnp P-value	nsp
13	TCGA-DA-A1HV-06	4435	TM	+	A34G	0.4967	yes

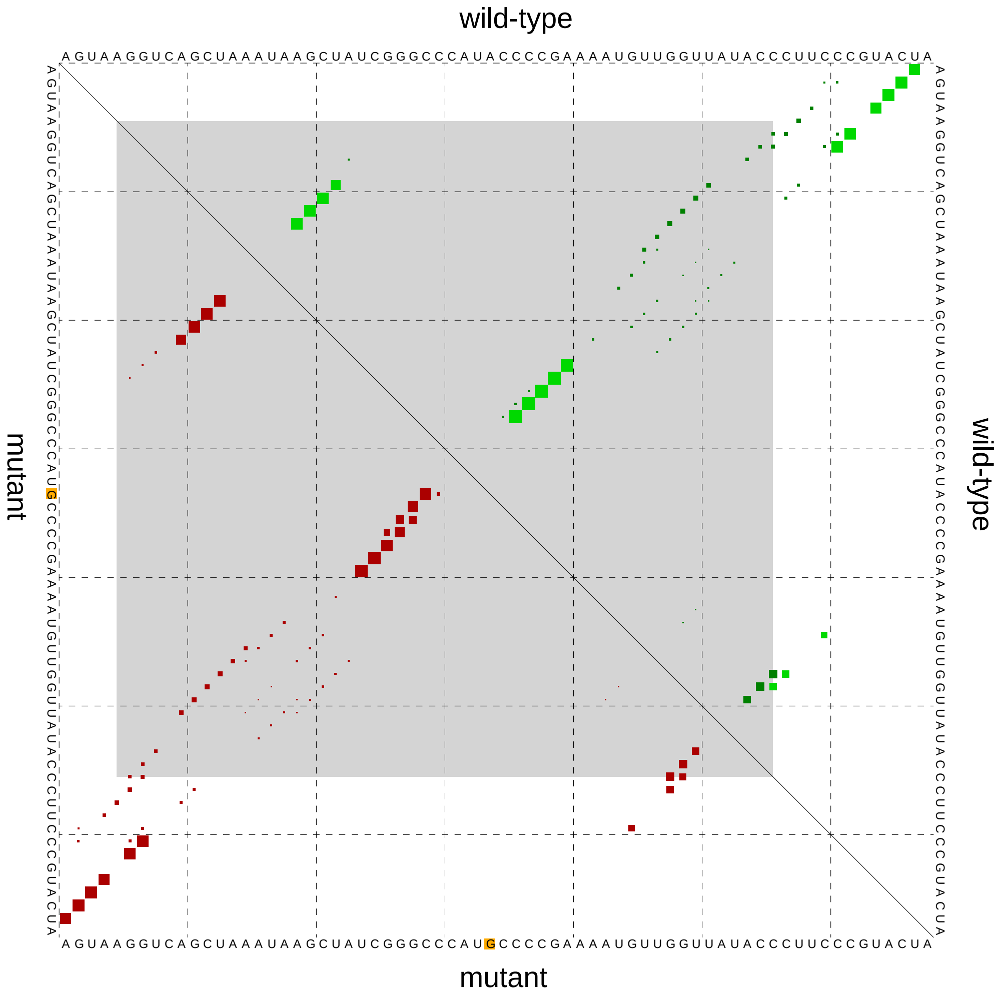

(a) Base-pair probabilities

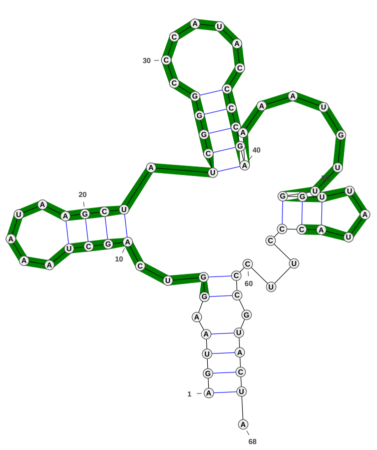

(b) wild-type

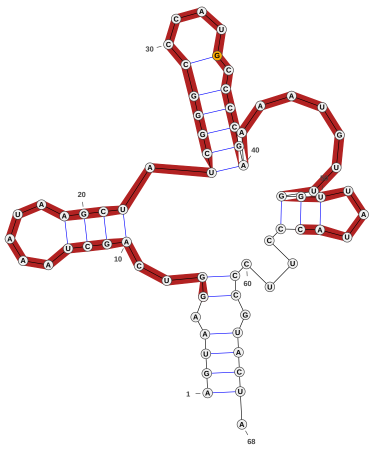

(c) mutant

Ref.	SampleID	chrPos	tRNA	Strand	mut	RNAsnp P-value	nsp
14	TCGA-60-2719-01	5610	TA	-	C46U	0.6013	yes

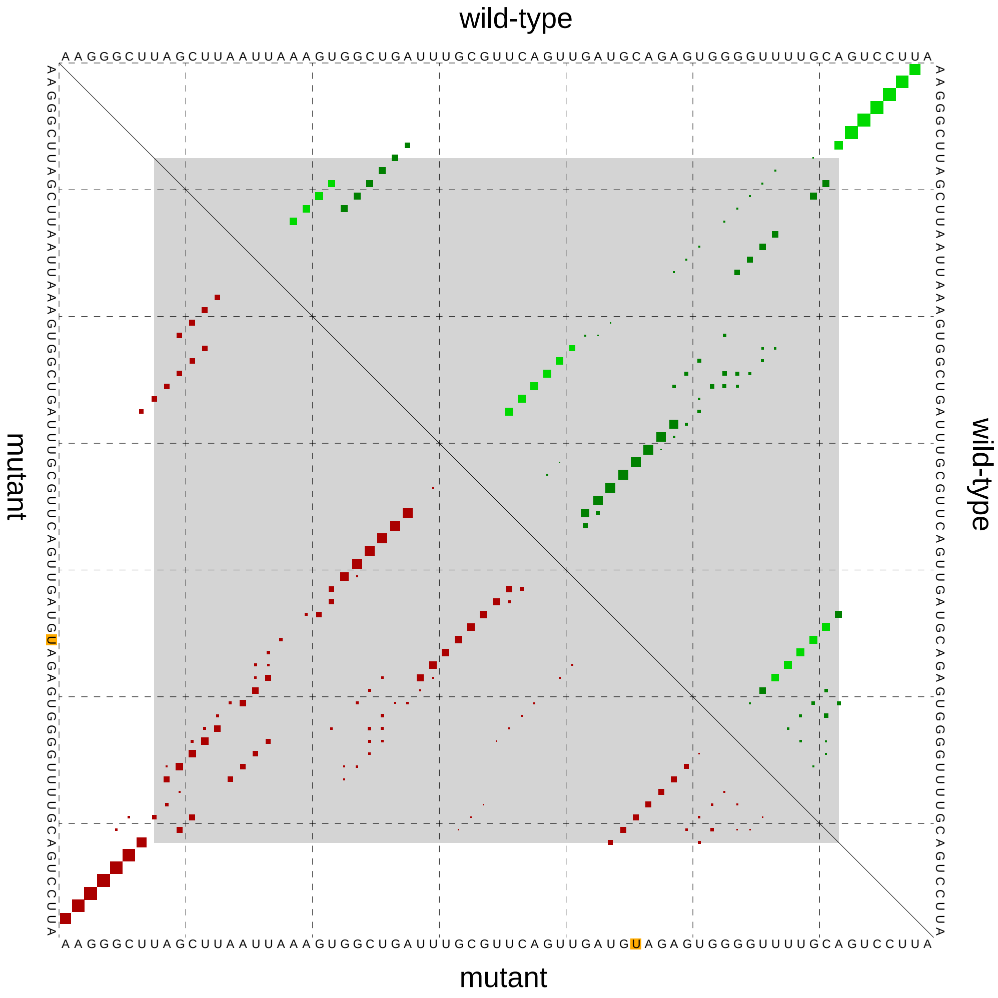

(a) Base-pair probabilities

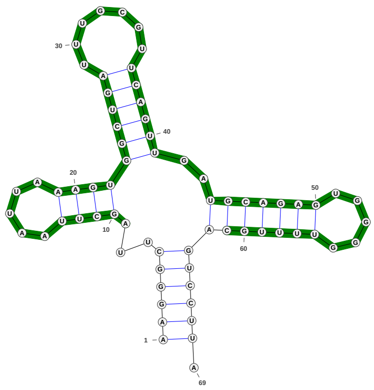

(b) wild-type

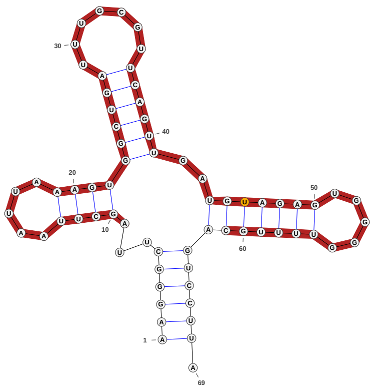

(c) mutant

Ref.	SampleID	chrPos	tRNA	Strand	mut	RNAsnp P-value	nsp
15	TCGA-EJ-5506-01	7566	TD	+	G49A	0.6307	yes

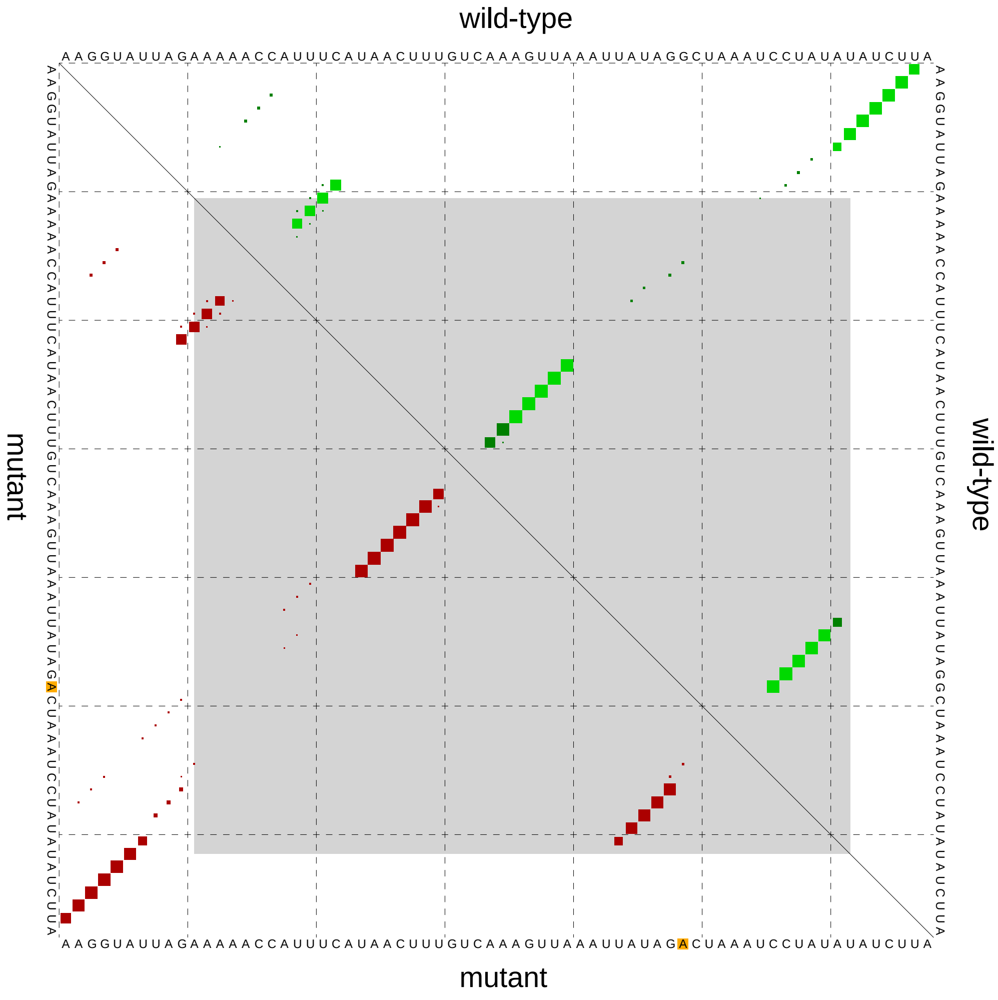

(a) Base-pair probabilities

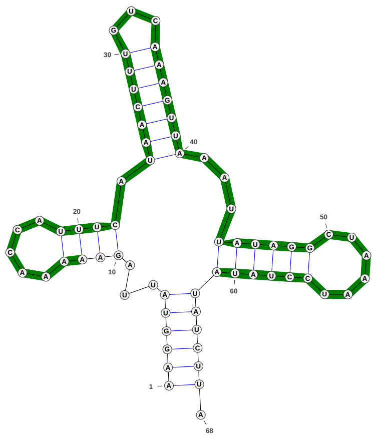

(b) wild-type

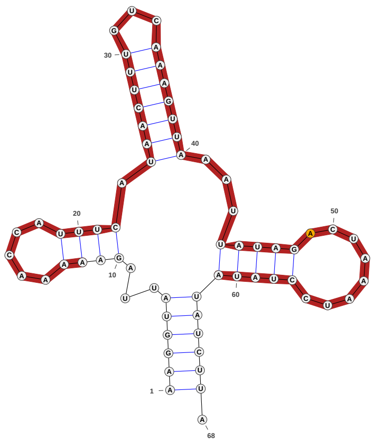

(c) mutant

Ref.	SampleID	chrPos	tRNA	Strand	mut	RNAsnp P-value	nsp
19	TCGA-05-5429-01	5609	TA	-	A47G	0.8757	yes

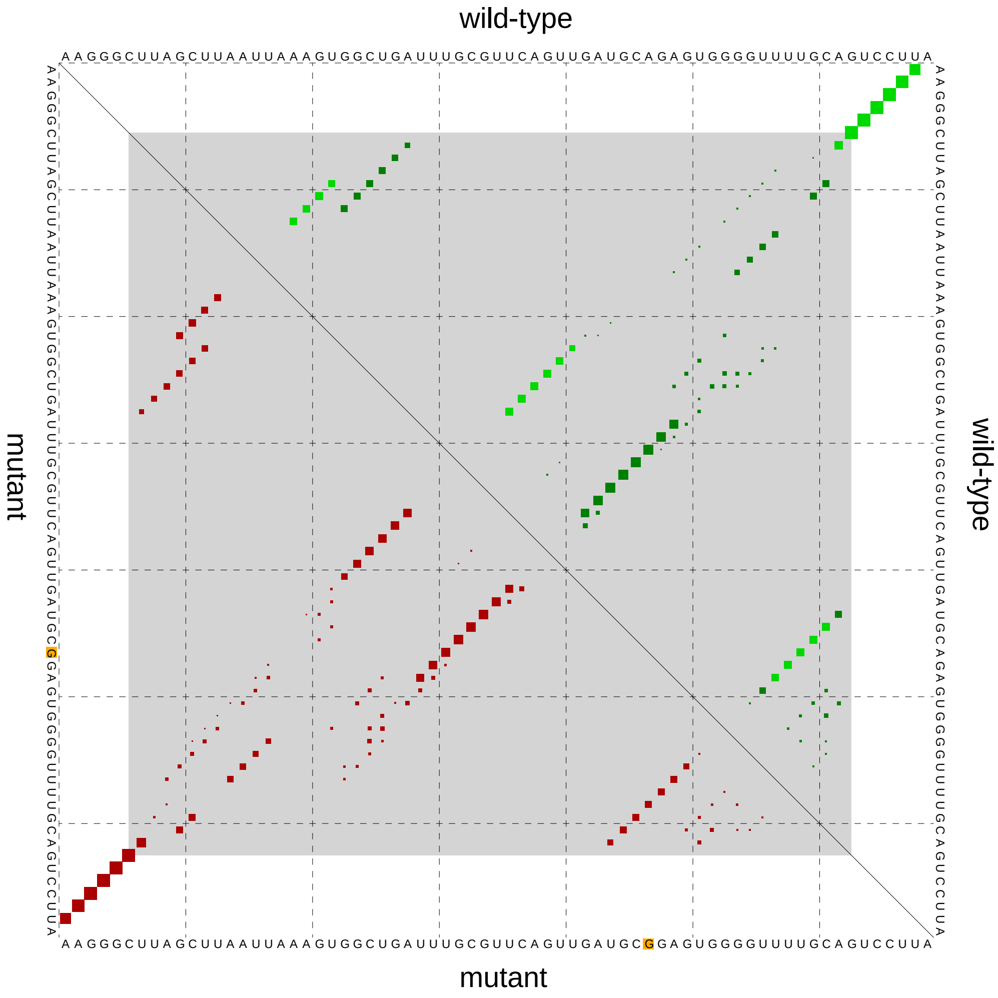

(a) Base-pair probabilities

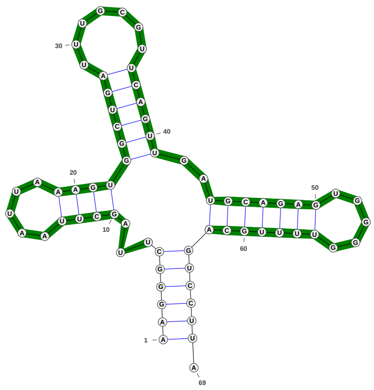

(b) wild-type

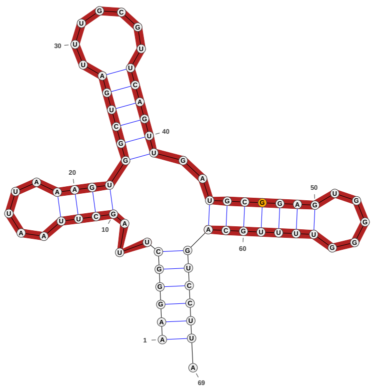

(c) mutant

Ref.	SampleID	chrPos	tRNA	Strand	mut	RNAsnp P-value	nsp
20	TCGA-06-0744-01	5590	TA	-	C66U	0.9360	yes

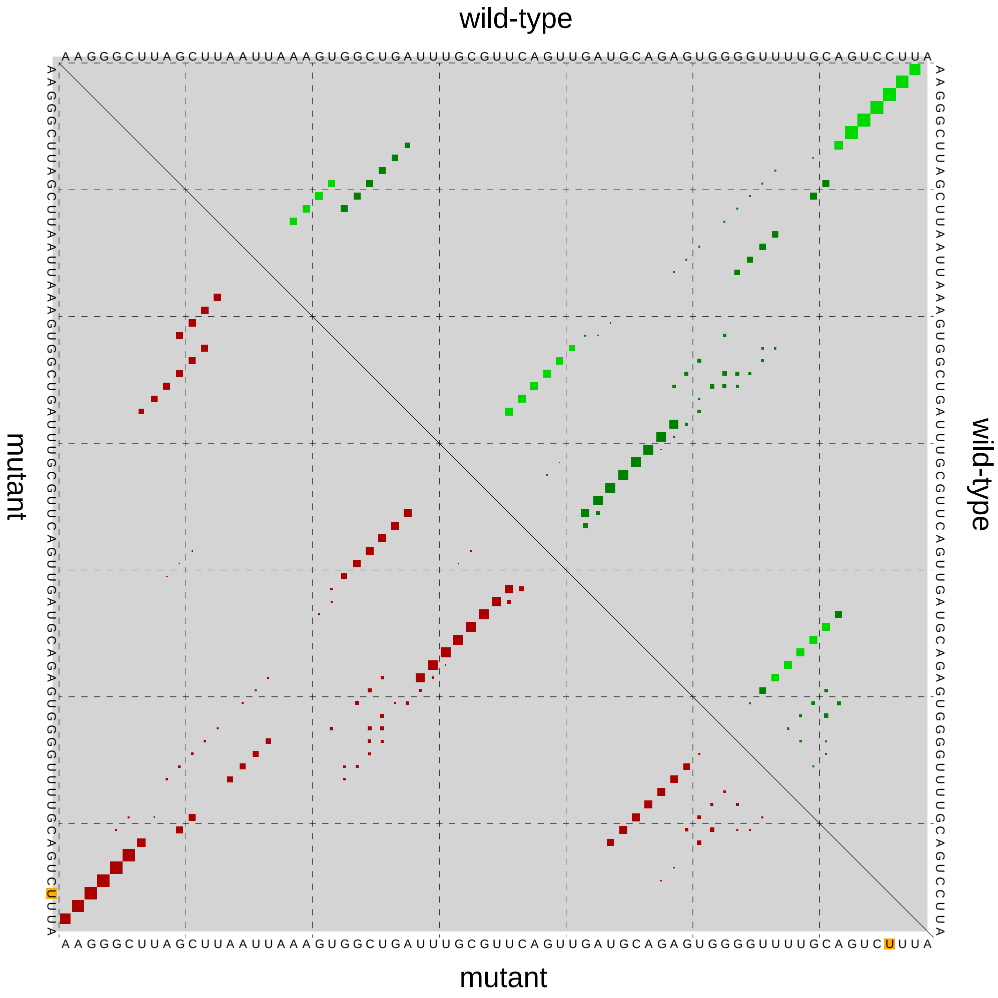

(a) Base-pair probabilities

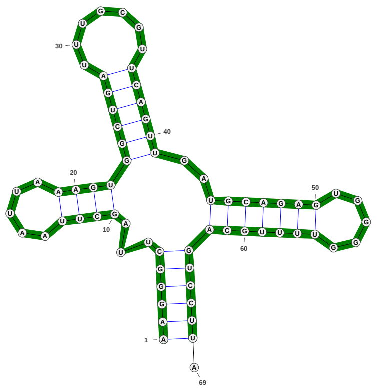

(b) wild-type

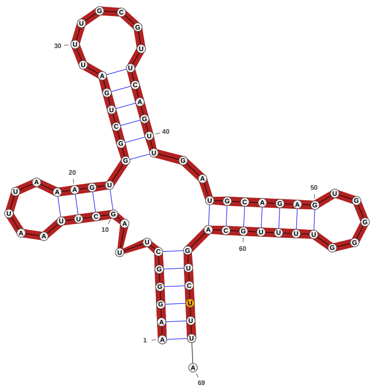

(c) mutant

Ref.	SampleID	chrPos	tRNA	Strand	mut	RNAsnp P-value	nsp
21	TCGA-B2-4099-01	4277	TI	+	U15C	0.9503	yes

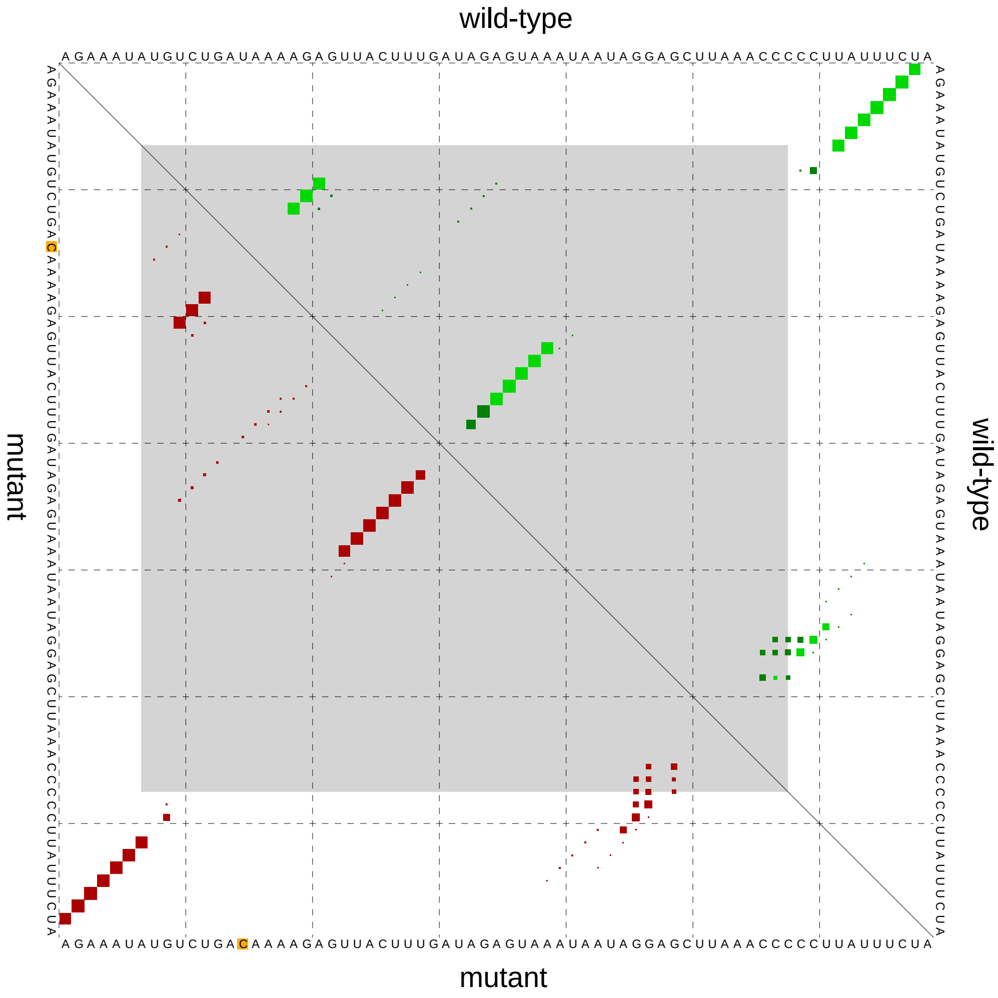

(a) Base-pair probabilities

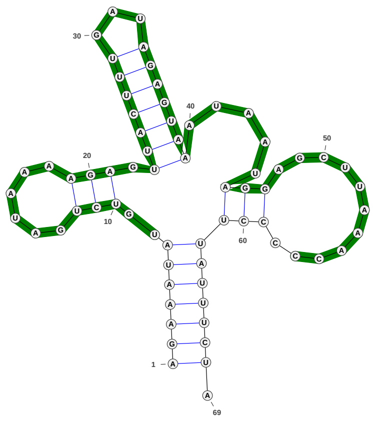

(b) wild-type

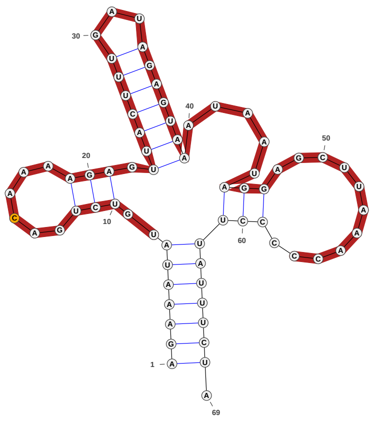

(c) mutant

Ref.	SampleID	chrPos	tRNA	Strand	mut	RNAsnp P-value	nsp
22	TCGA-BP-4781-01	10042	TG	+	A52G	0.9713	yes

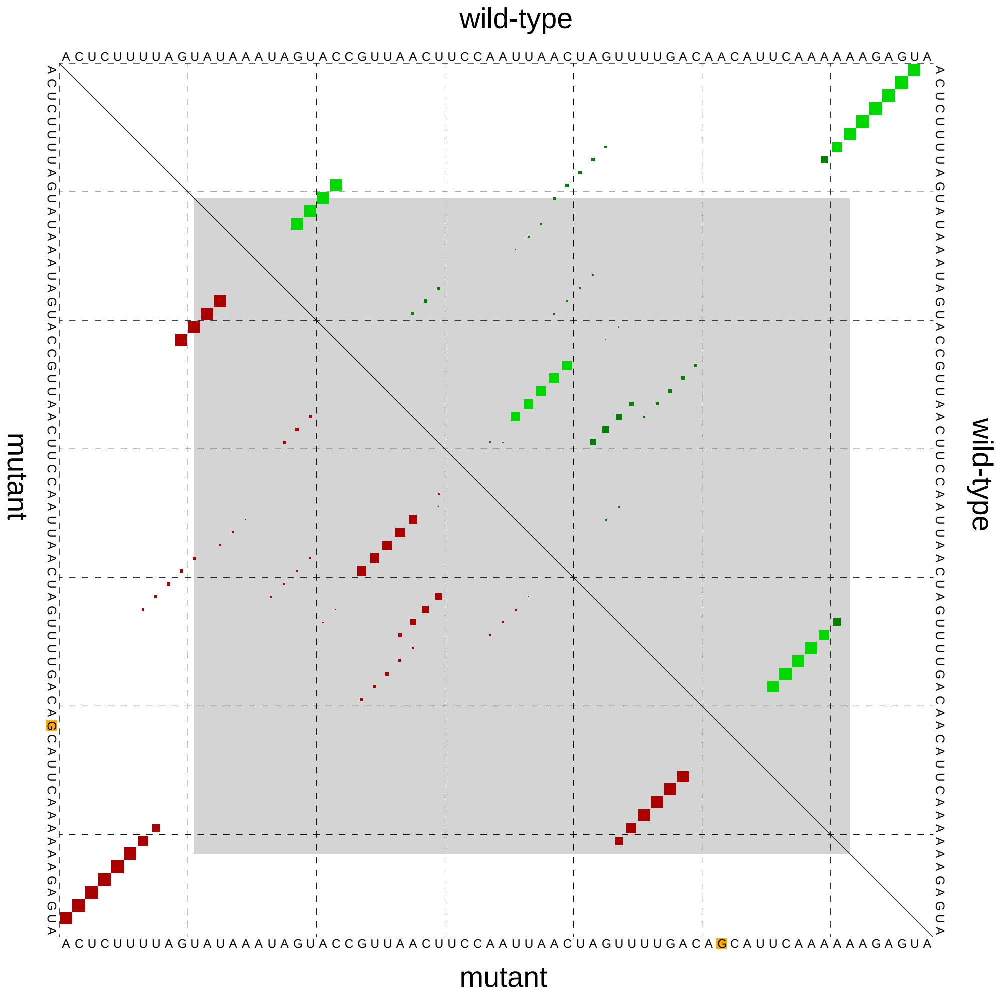

(a) Base-pair probabilities

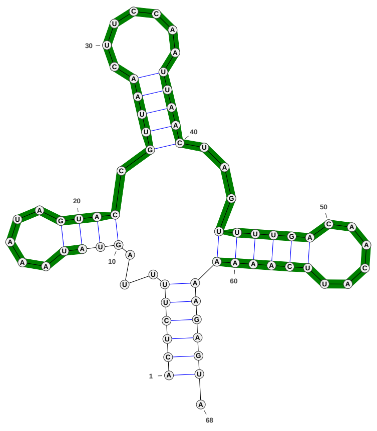

(b) wild-type

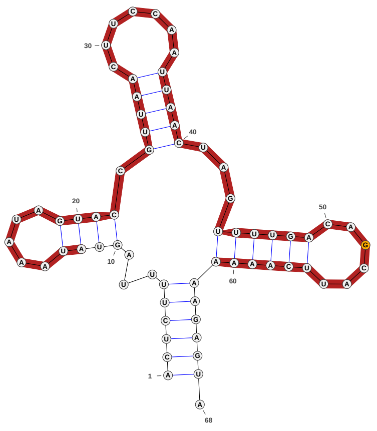

(c) mutant

Ref.	SampleID	chrPos	tRNA	Strand	mut	RNAsnp P-value	nsp
11	TCGA-EM-A3AL-01	3244	TL1	+	G15A	0.4013	no

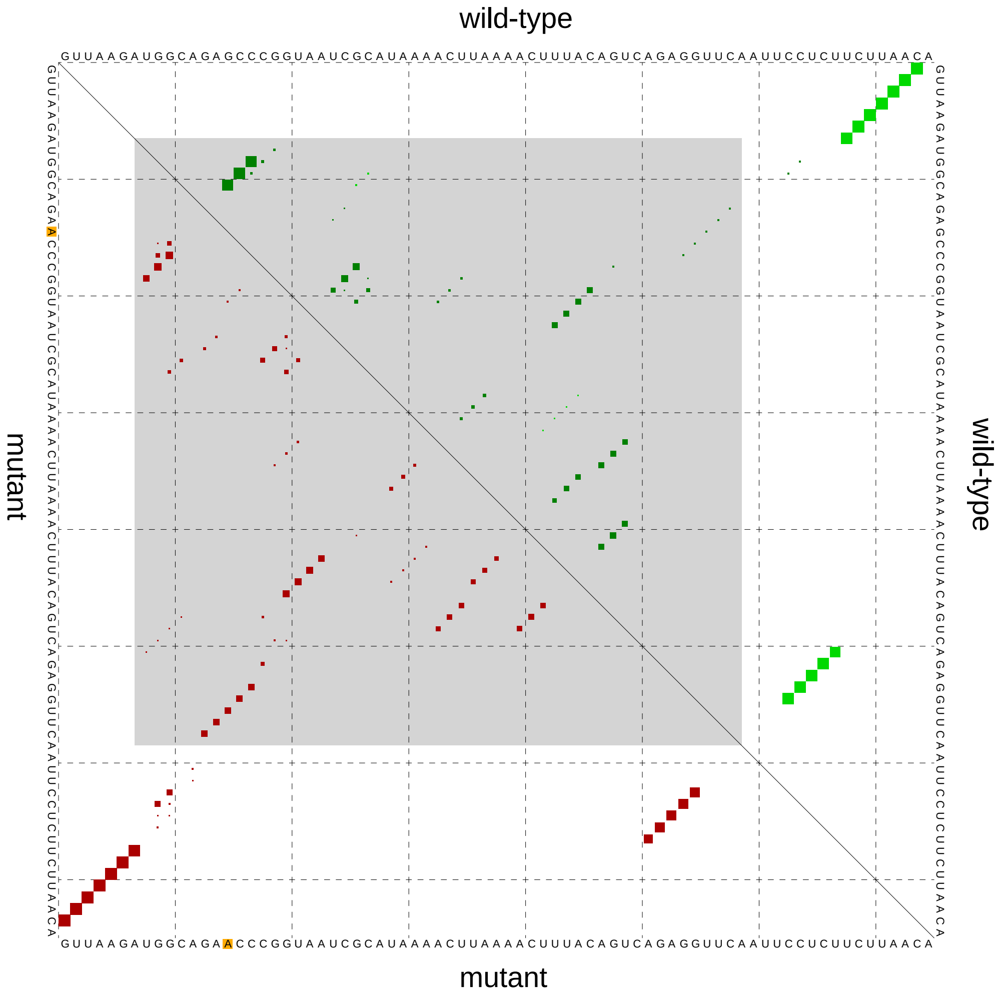

(a) Base-pair probabilities

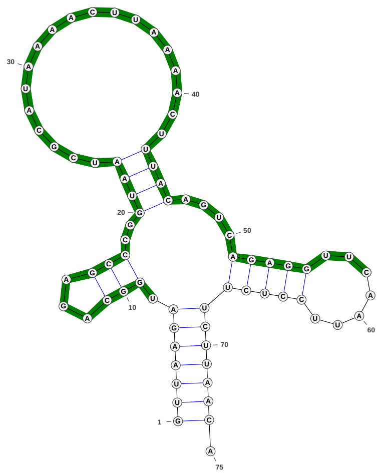

(b) wild-type

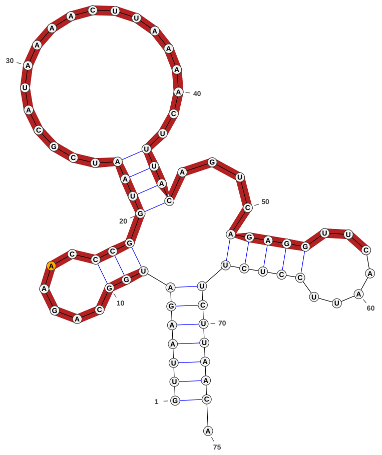

(c) mutant

Ref.	SampleID	chrPos	tRNA	Strand	mut	RNAsnp P-value	nsp
23	TCGA-CR-6470-01	3294	TL1	+	U65C	0.0533	no

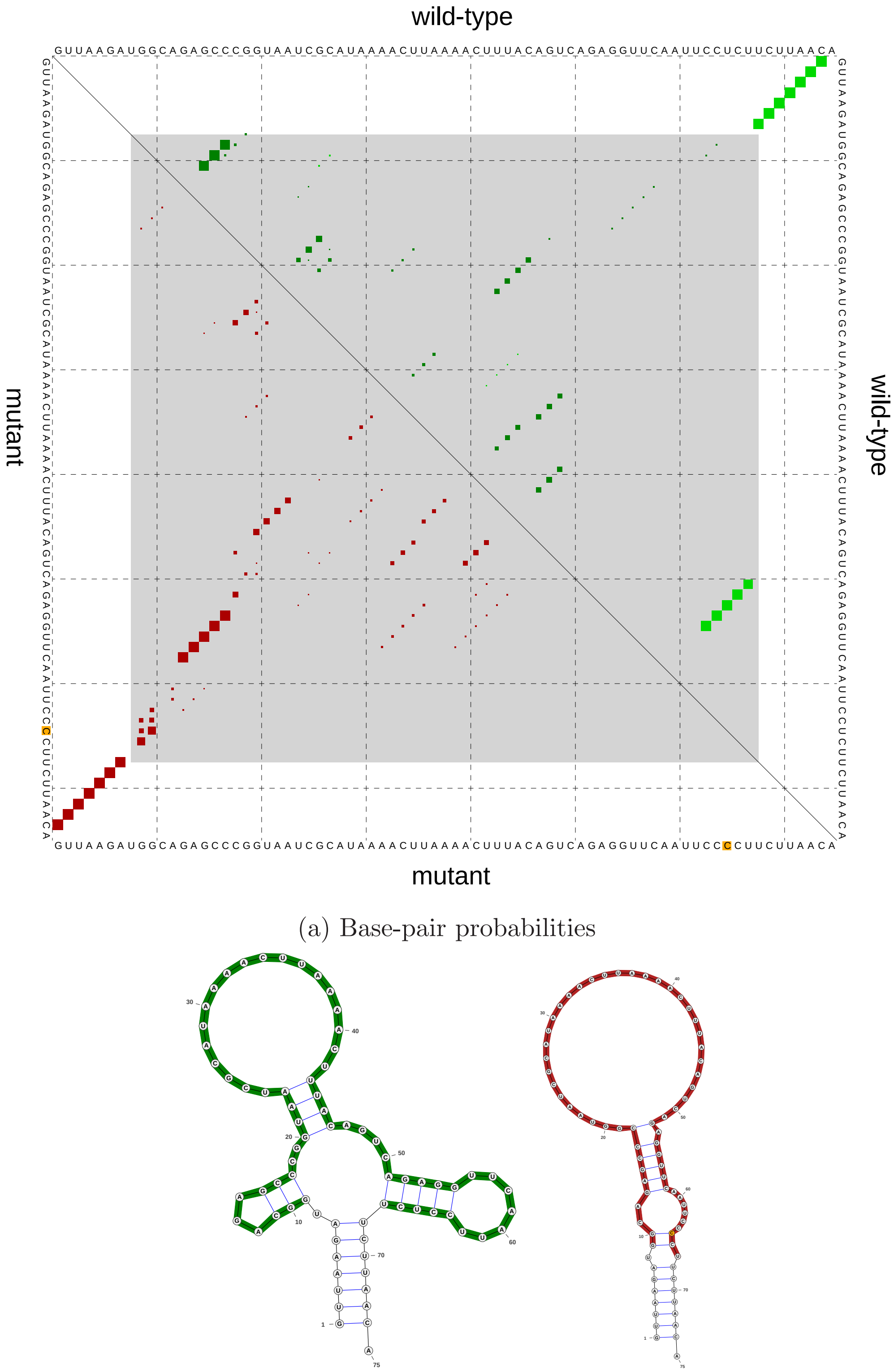

(a) Base-pair probabilities

(b) wild-type (c) mutant

Ref.	SampleID	chrPos	tRNA	Strand	mut	RNAsnp P-value	nsp
24	TCGA-DA-A3F5-06	586	TF	+	G10A	0.0710	no

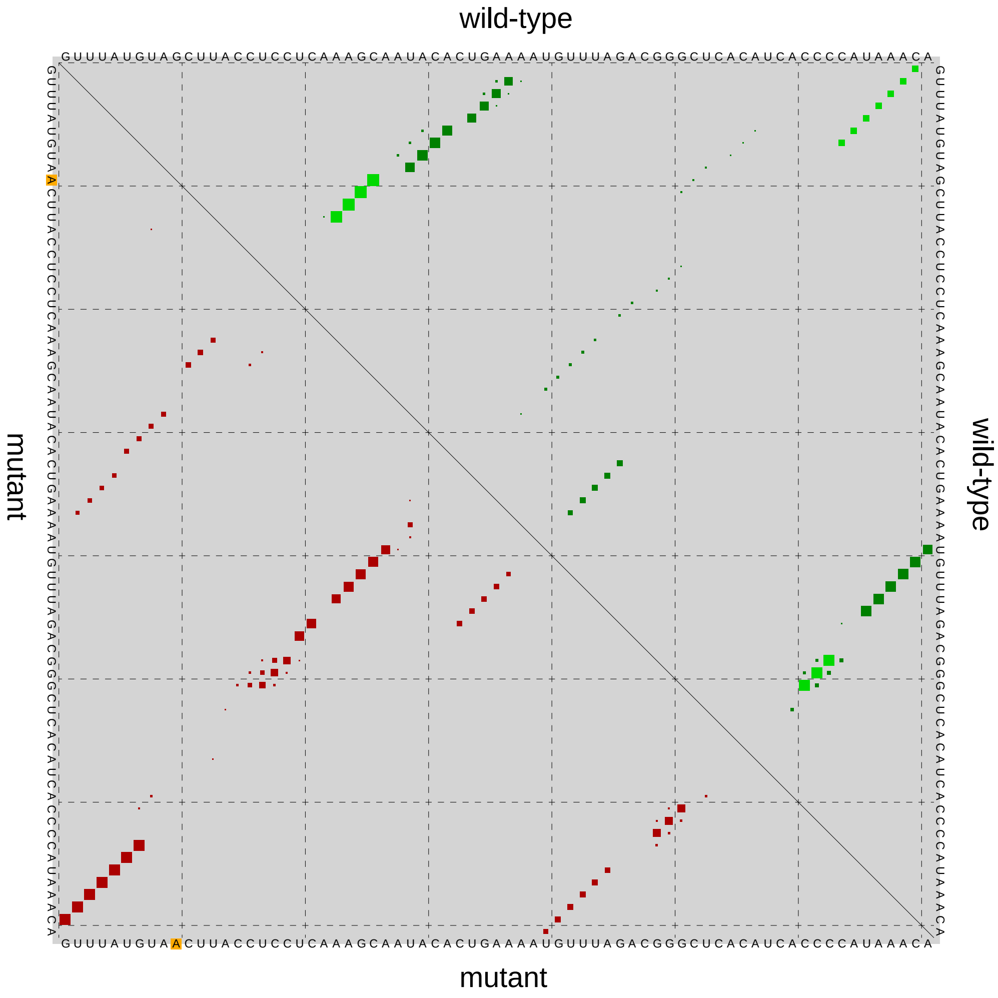

(a) Base-pair probabilities

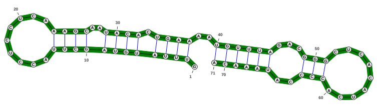

(b) wild-type

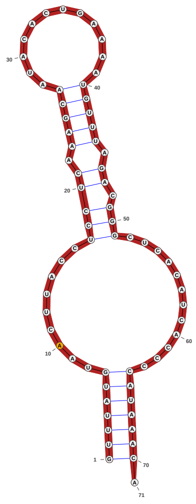

(c) mutant

Ref.	SampleID	chrPos	tRNA	Strand	mut	RNAsnp P-value	nsp
25	TCGA-60-2724-01	1604	TV	+	G3A	0.0917	no

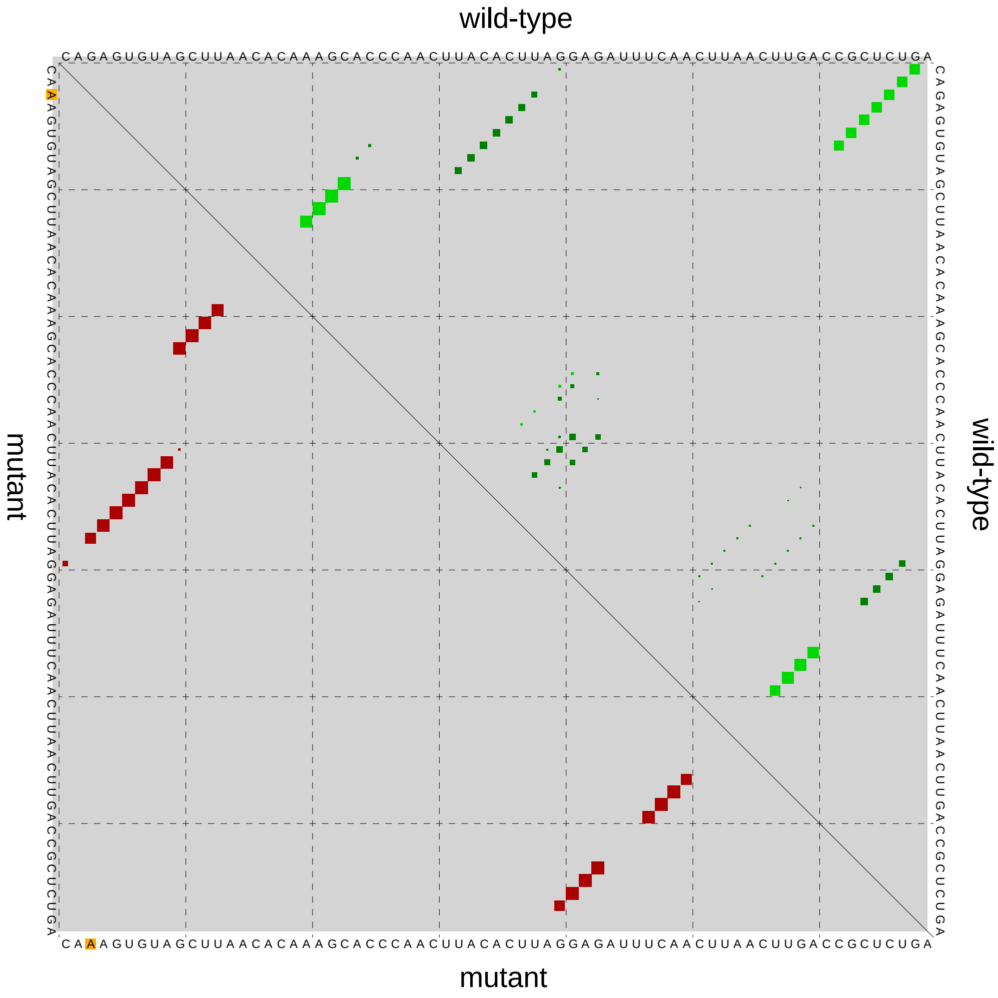

(a) Base-pair probabilities

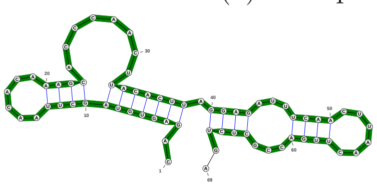

(b) wild-type

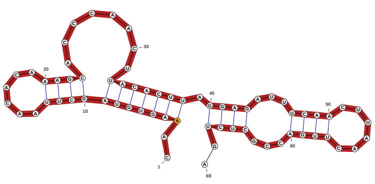

(c) mutant

Ref.	SampleID	chrPos	tRNA	Strand	mut	RNAsnp P-value	nsp
26	TCGA-ET-A4KN-01	12319	TL2	+	C54U	0.1010	no

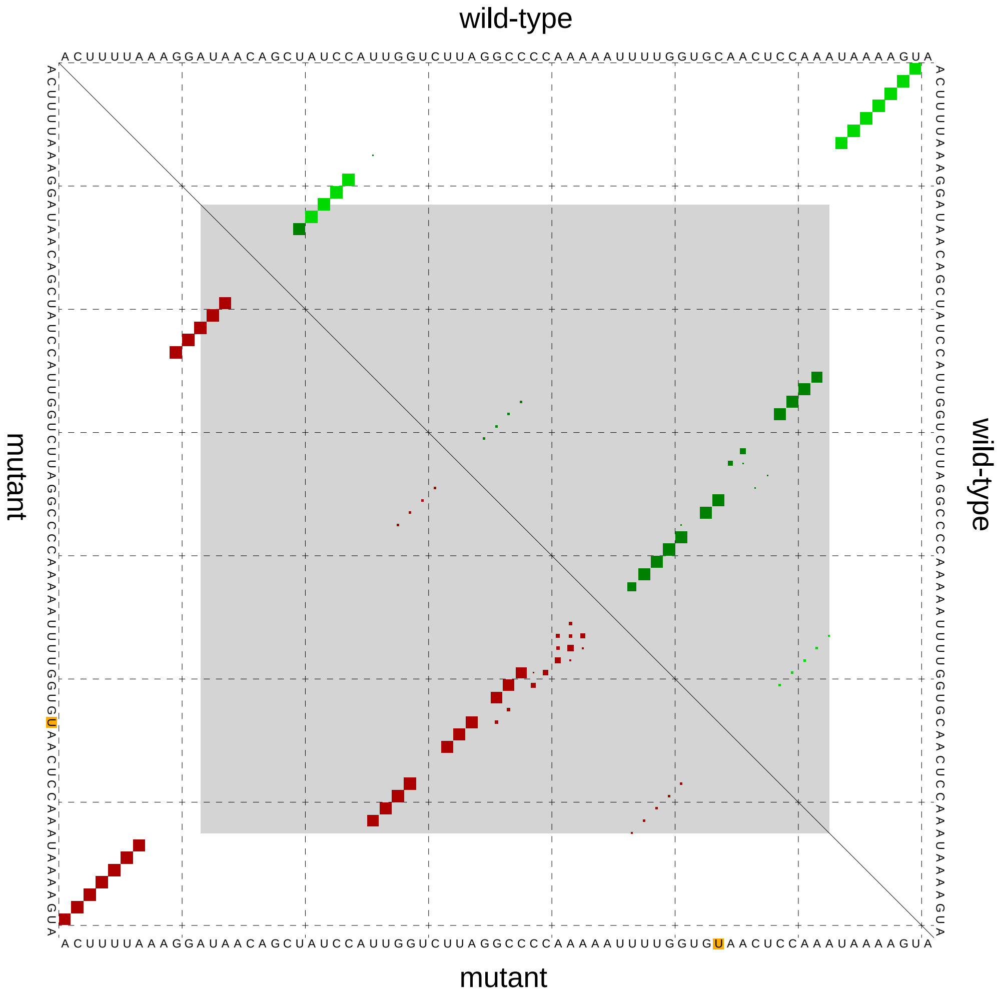

(a) Base-pair probabilities

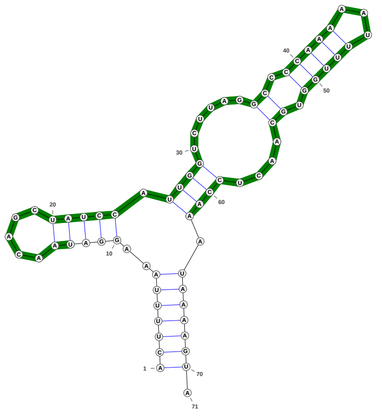

(b) wild-type

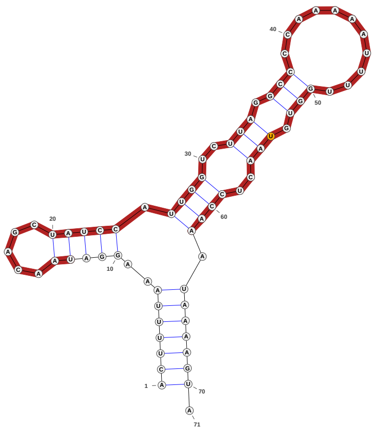

(c) mutant

Ref.	SampleID	chrPos	tRNA	Strand	mut	RNAsnp P-value	nsp
27	TCGA-05-4398-01	15892	TT	+	U5C	0.1050	no

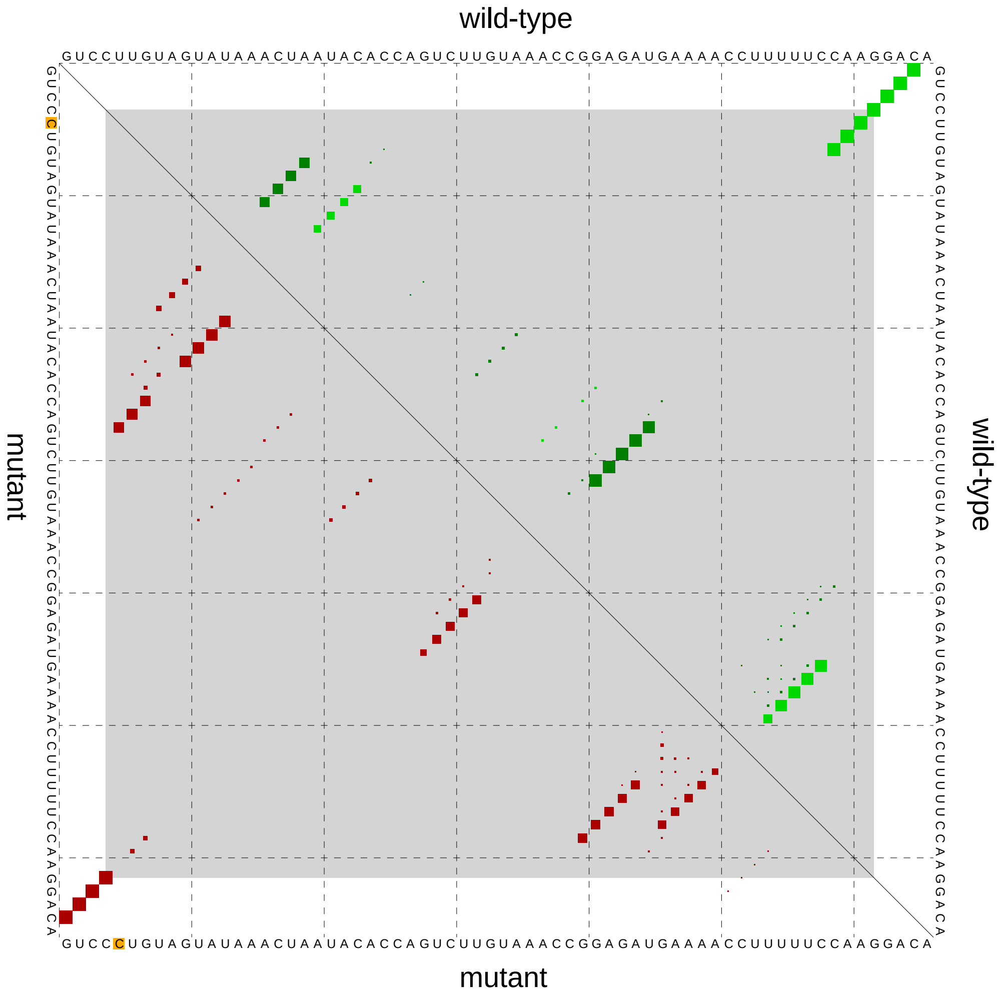

(a) Base-pair probabilities

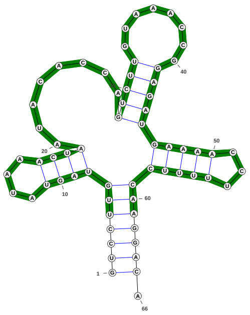

(b) wild-type

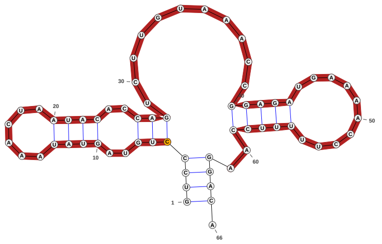

(c) mutant

Ref.	SampleID	chrPos	tRNA	Strand	mut	RNAsnp P-value	nsp
28	TCGA-AP-A05A-01	5791	TC	-	C36U	0.1893	no

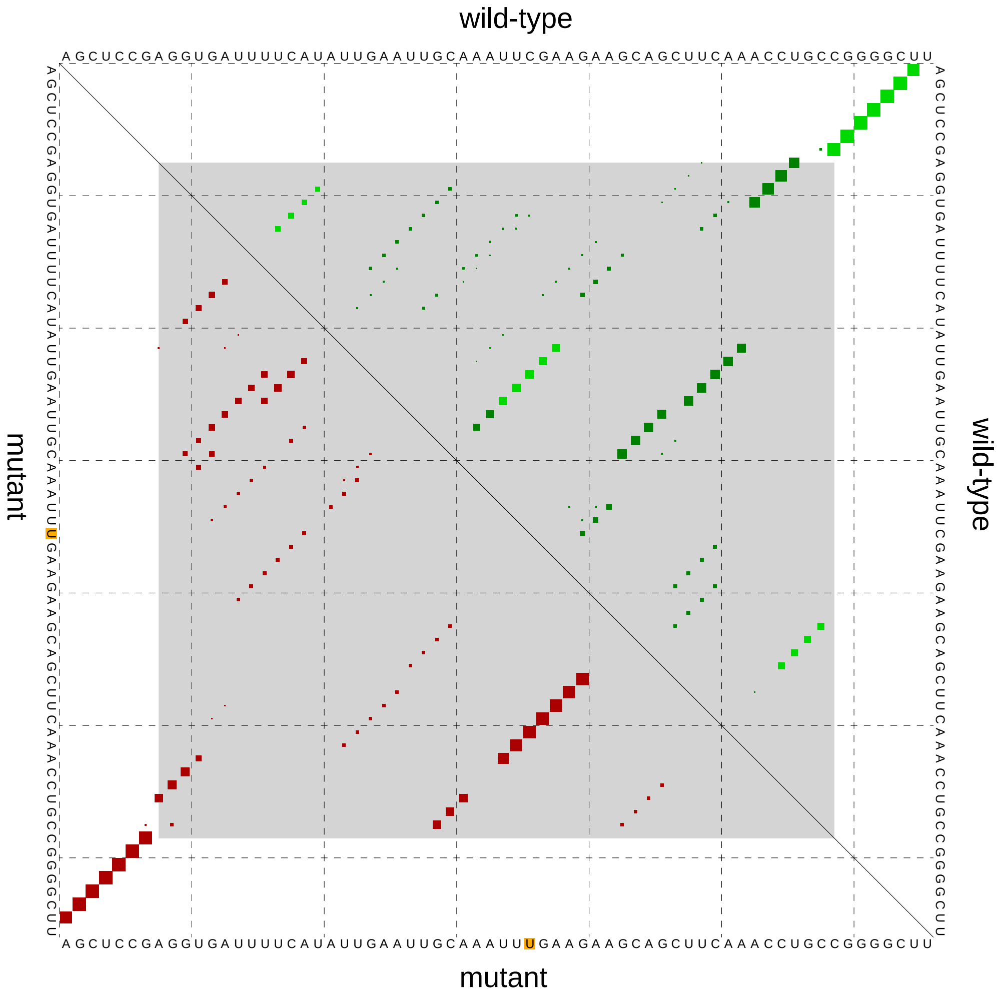

(a) Base-pair probabilities

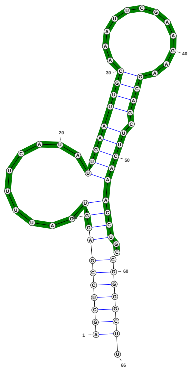

(b) wild-type

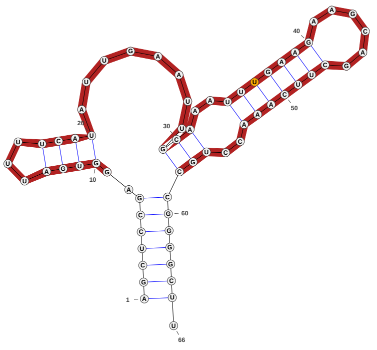

(c) mutant

Ref.	SampleID	chrPos	tRNA	Strand	mut	RNAsnp P-value	nsp
29	TCGA-DK-A1AA-01	5668	TN	-	C62U	0.2697	no

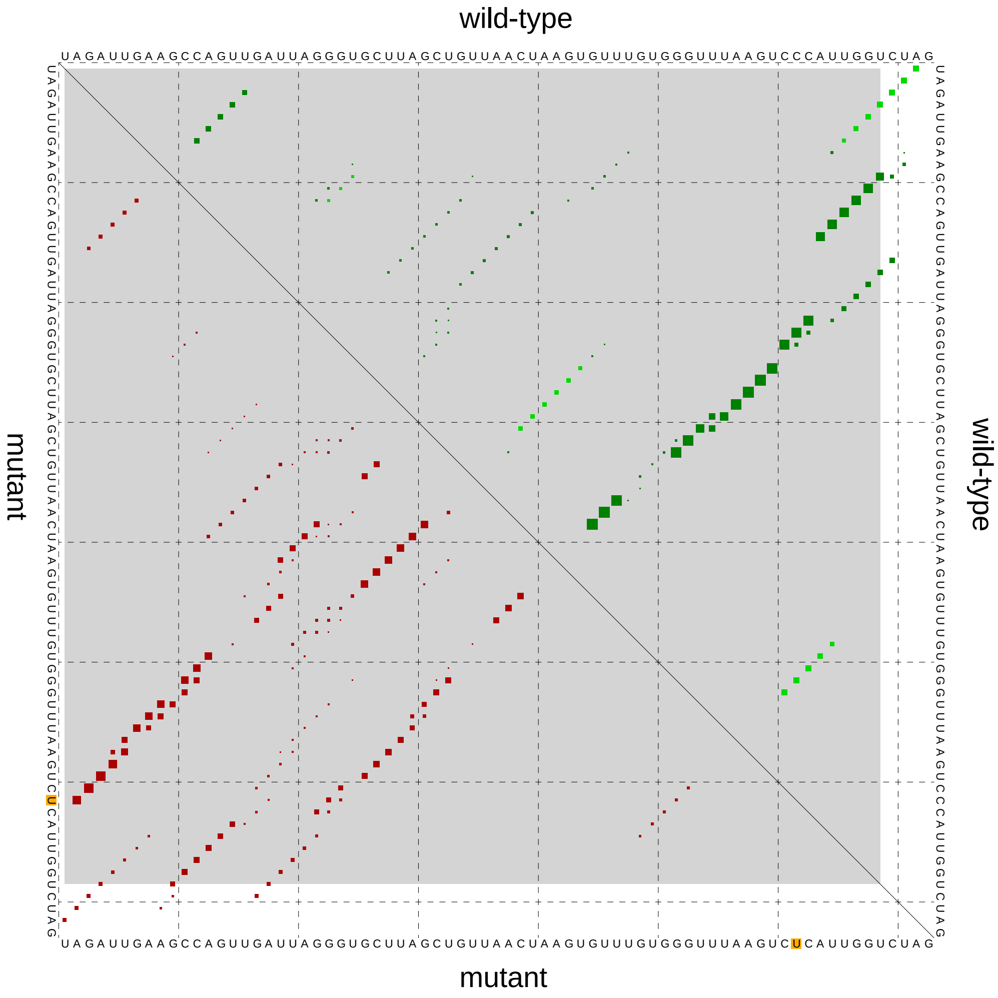

(a) Base-pair probabilities

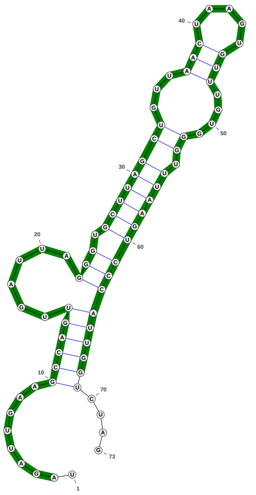

(b) wild-type

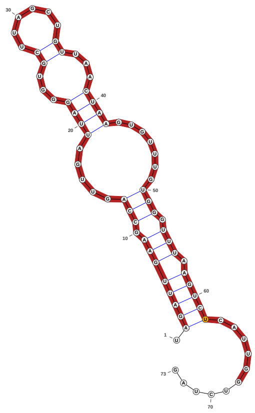

(c) mutant

Ref.	SampleID	chrPos	tRNA	Strand	mut	RNAsnp P-value	nsp
30	TCGA-EL-A3CV-01	3239	TL1	+	G10A	0.3030	no

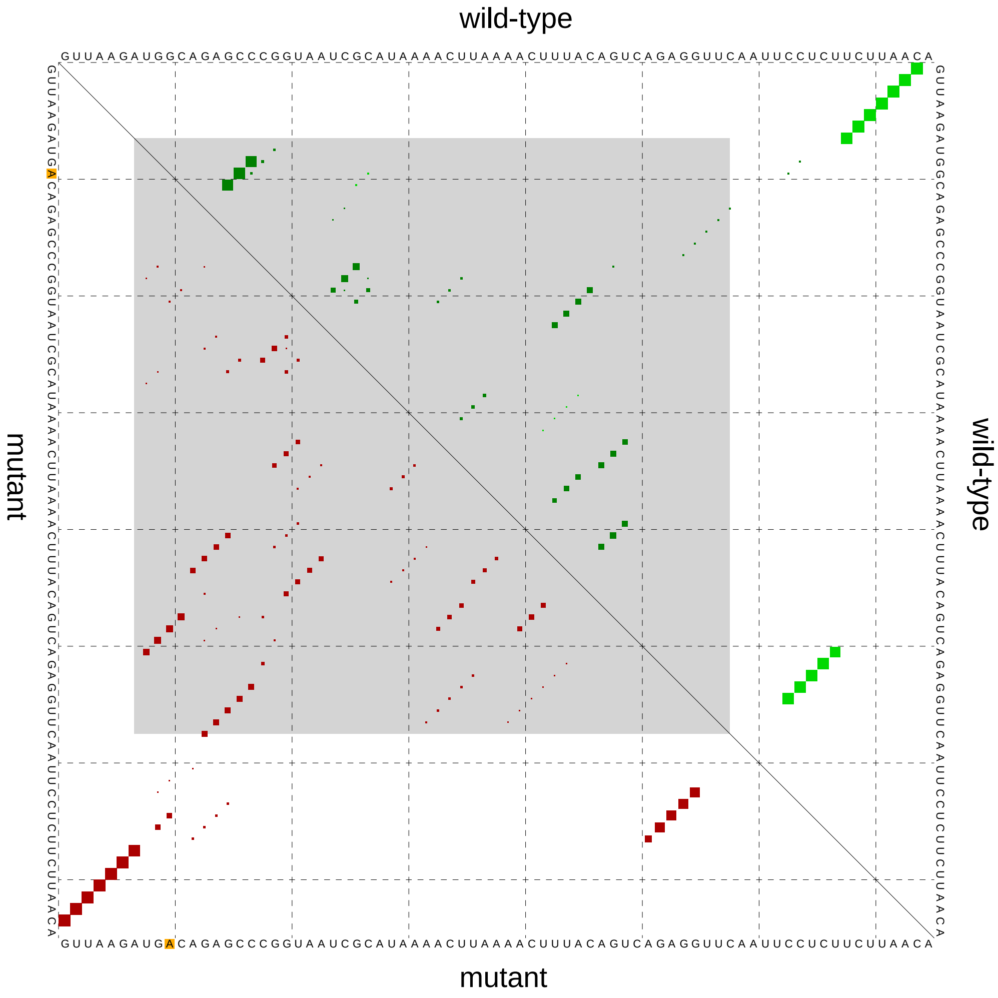

(a) Base-pair probabilities

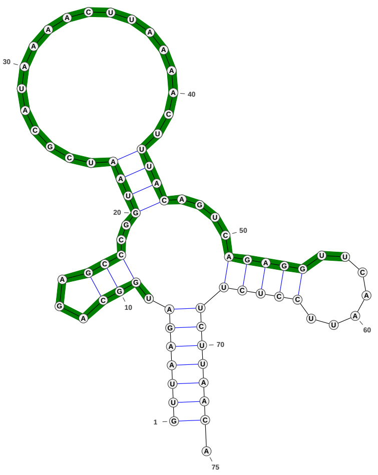

(b) wild-type

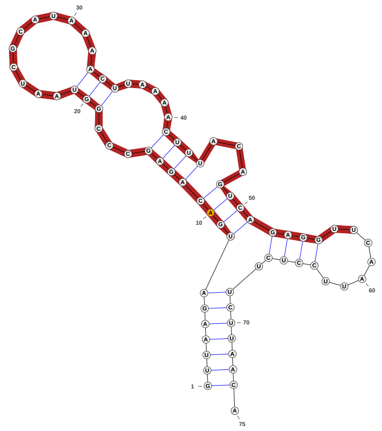

(c) mutant

Ref.	SampleID	chrPos	tRNA	Strand	mut	RNAsnp P-value	nsp
31	TCGA-DK-A1AA-01	10457	TR	+	U53C	0.3203	no

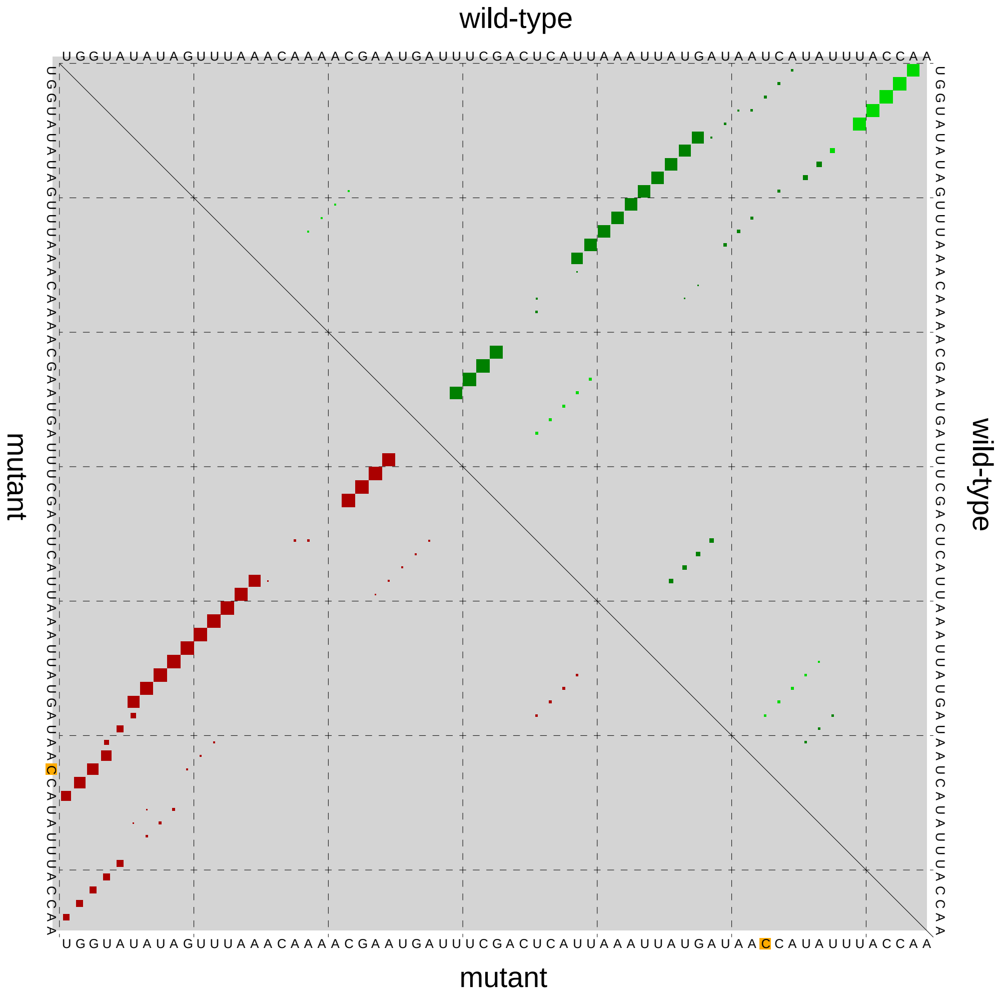

(a) Base-pair probabilities

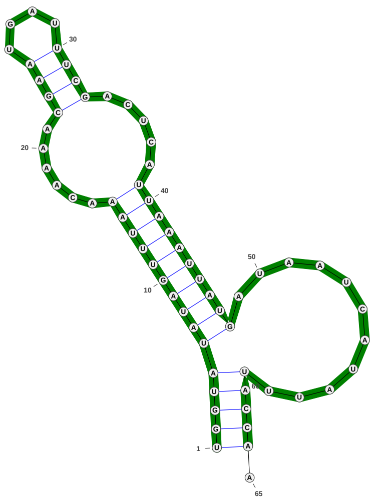

(b) wild-type

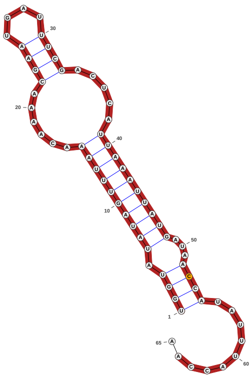

(c) mutant

Ref.	SampleID	chrPos	tRNA	Strand	mut	RNAsnp P-value	nsp
32	TCGA-KN-8424-01	15897	TT	+	G10A	0.3797	no

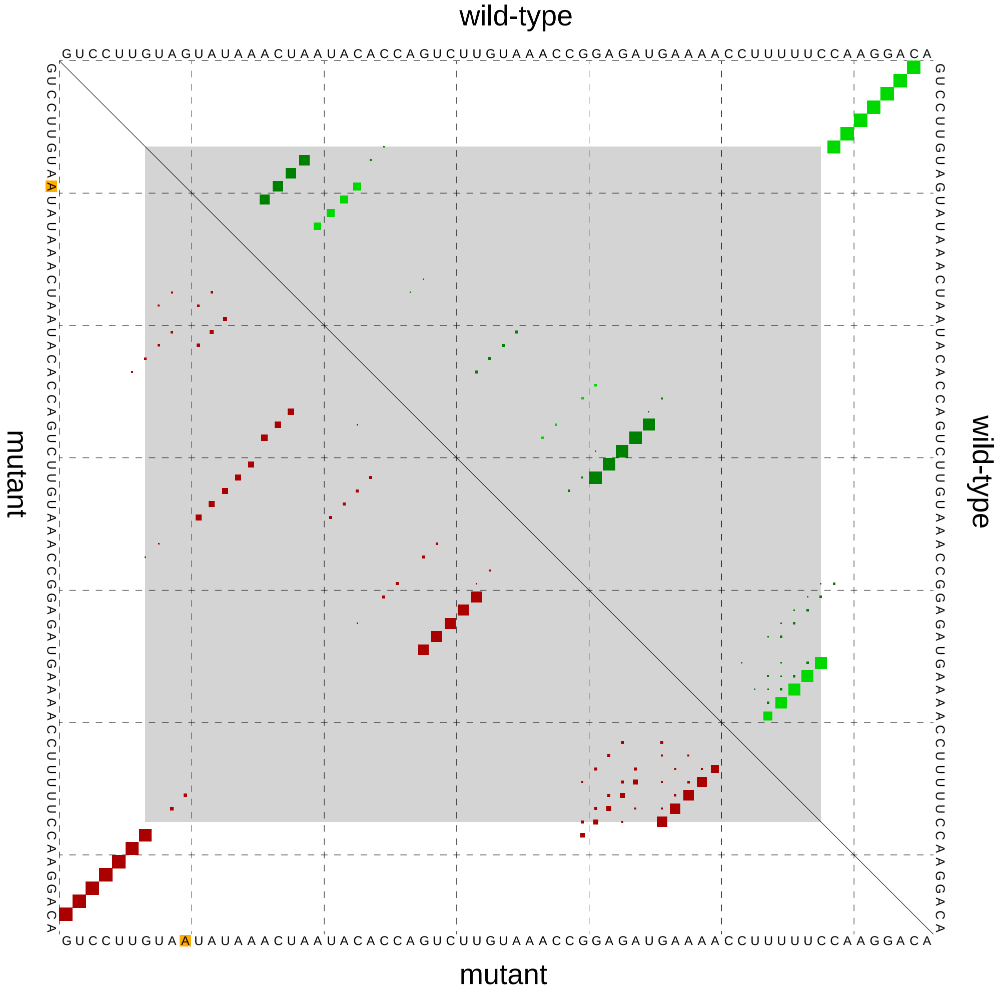

(a) Base-pair probabilities

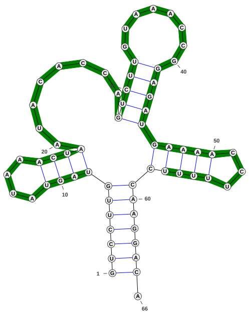

(b) wild-type

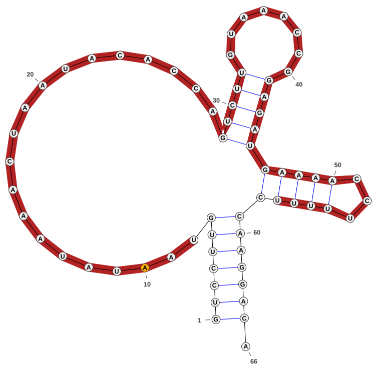

(c) mutant

Ref.	SampleID	chrPos	tRNA	Strand	mut	RNAsnp P-value	nsp
33	TCGA-CV-6961-01	5571	TW	+	U60C	0.4000	no

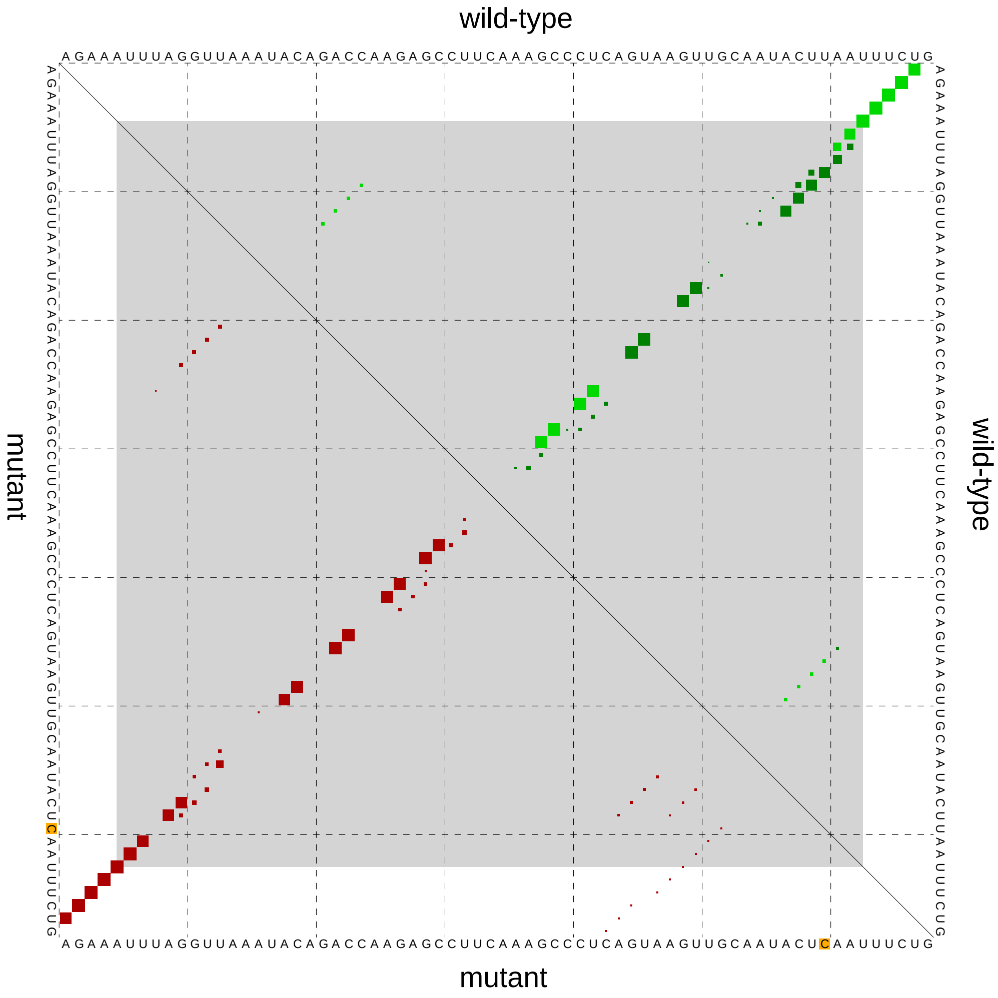

(a) Base-pair probabilities

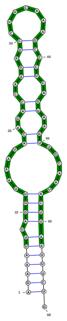

(b) wild-type

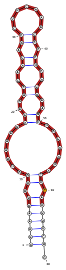

(c) mutant

Ref.	SampleID	chrPos	tRNA	Strand	mut	RNAsnp P-value	nsp
34	TCGA-CW-6087-01	5814	TC	-	A13G	0.4267	no

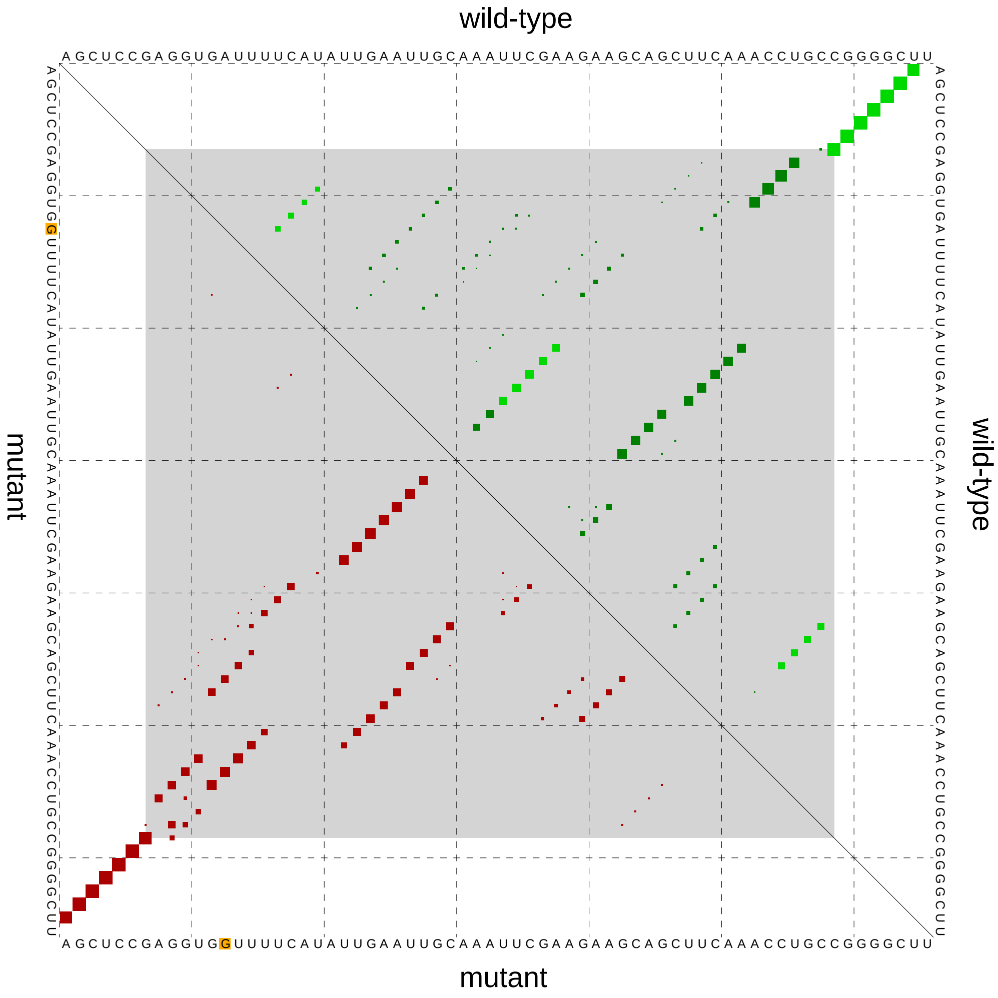

(a) Base-pair probabilities

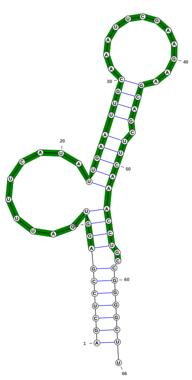

(b) wild-type

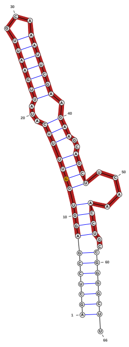

(c) mutant

Ref.	SampleID	chrPos	tRNA	Strand	mut	RNAsnp P-value	nsp
35	TCGA-05-4396-01	12213	TS2	+	G7A	0.4380	no

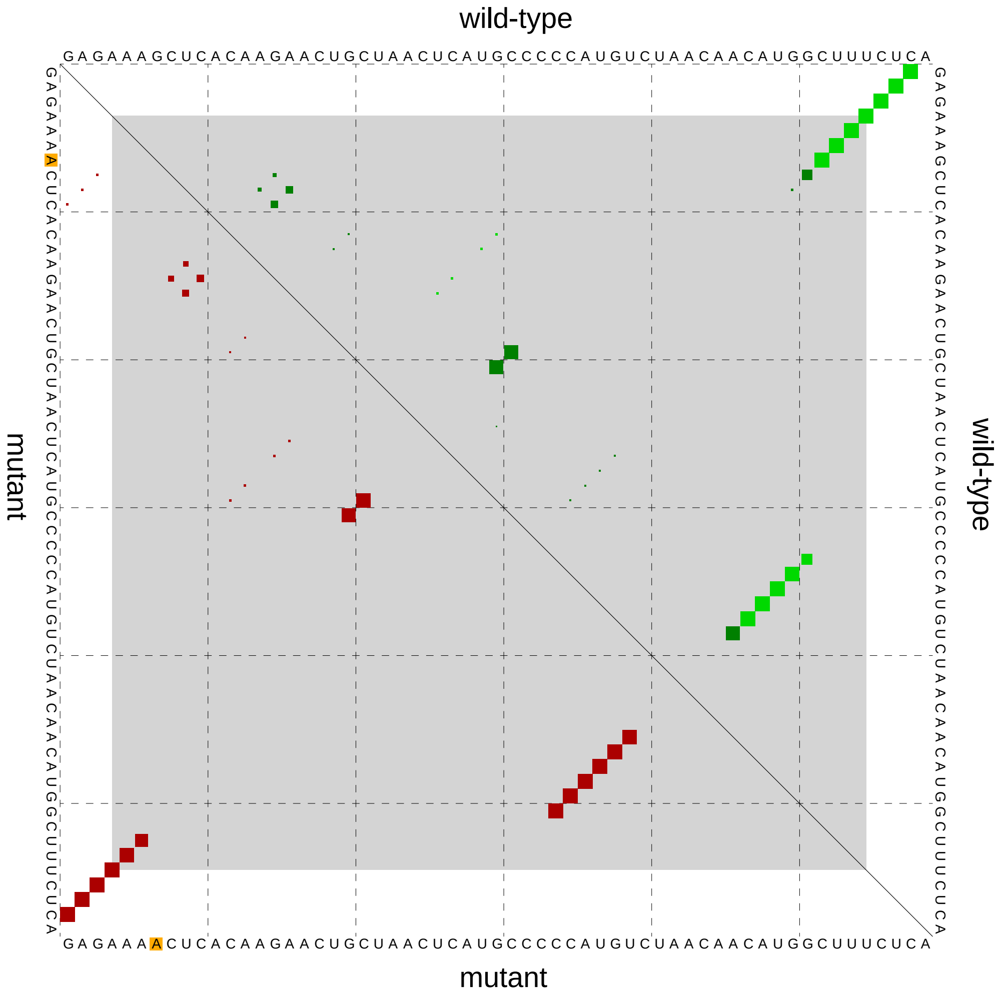

(a) Base-pair probabilities

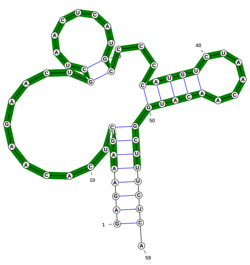

(b) wild-type

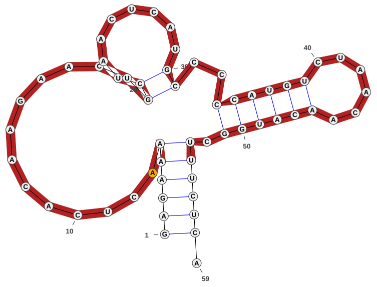

(c) mutant

Ref.	SampleID	chrPos	tRNA	Strand	mut	RNAsnp P-value	nsp
36	TCGA-A2-A3Y0-01	8328	TK	+	G34A	0.4443	no

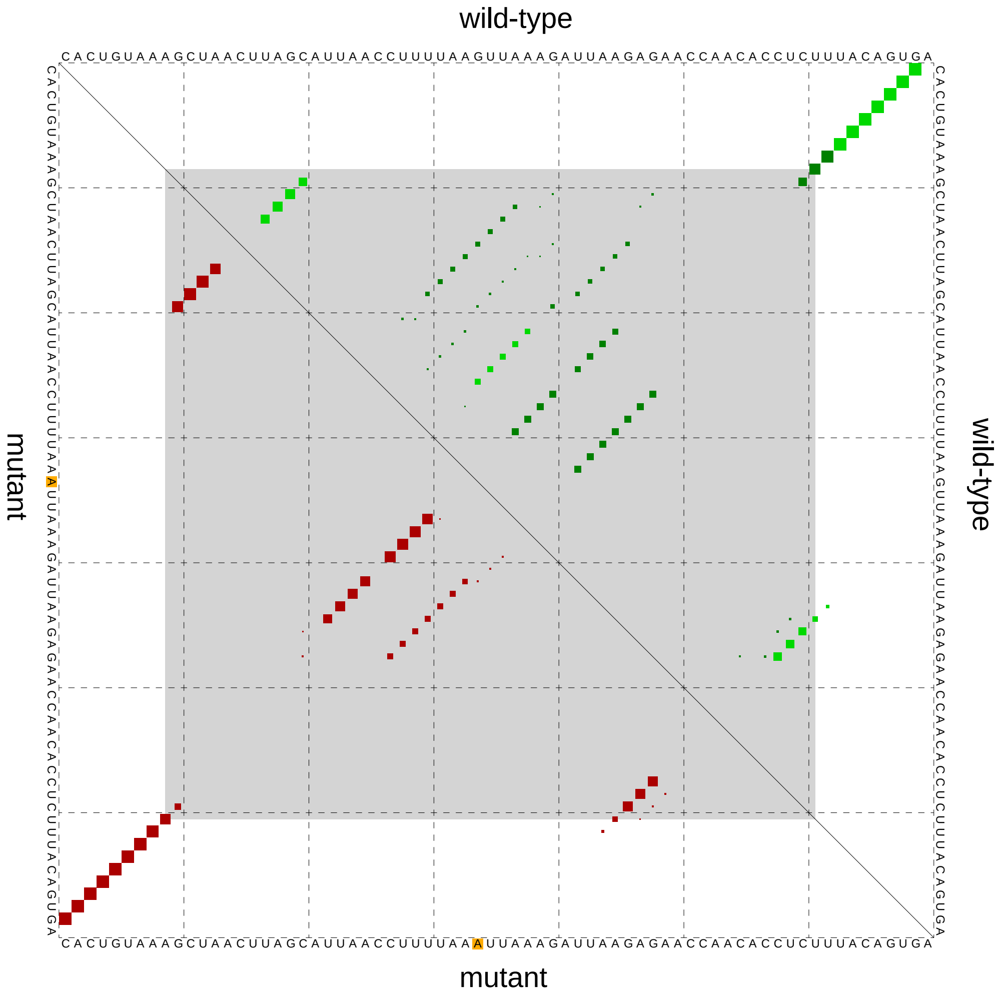

(a) Base-pair probabilities

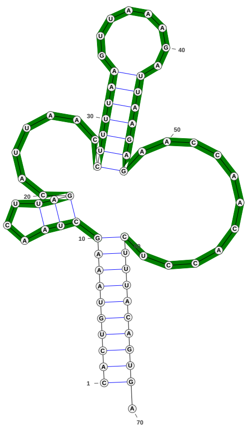

(b) wild-type

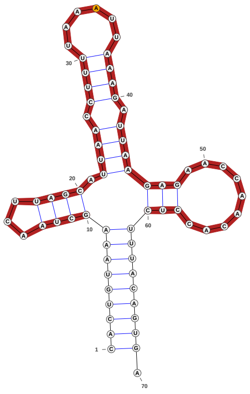

(c) mutant

Ref.	SampleID	chrPos	tRNA	Strand	mut	RNAsnp P-value	nsp
37	TCGA-A8-A092-01	16019	TP	-	G5U	0.5093	no

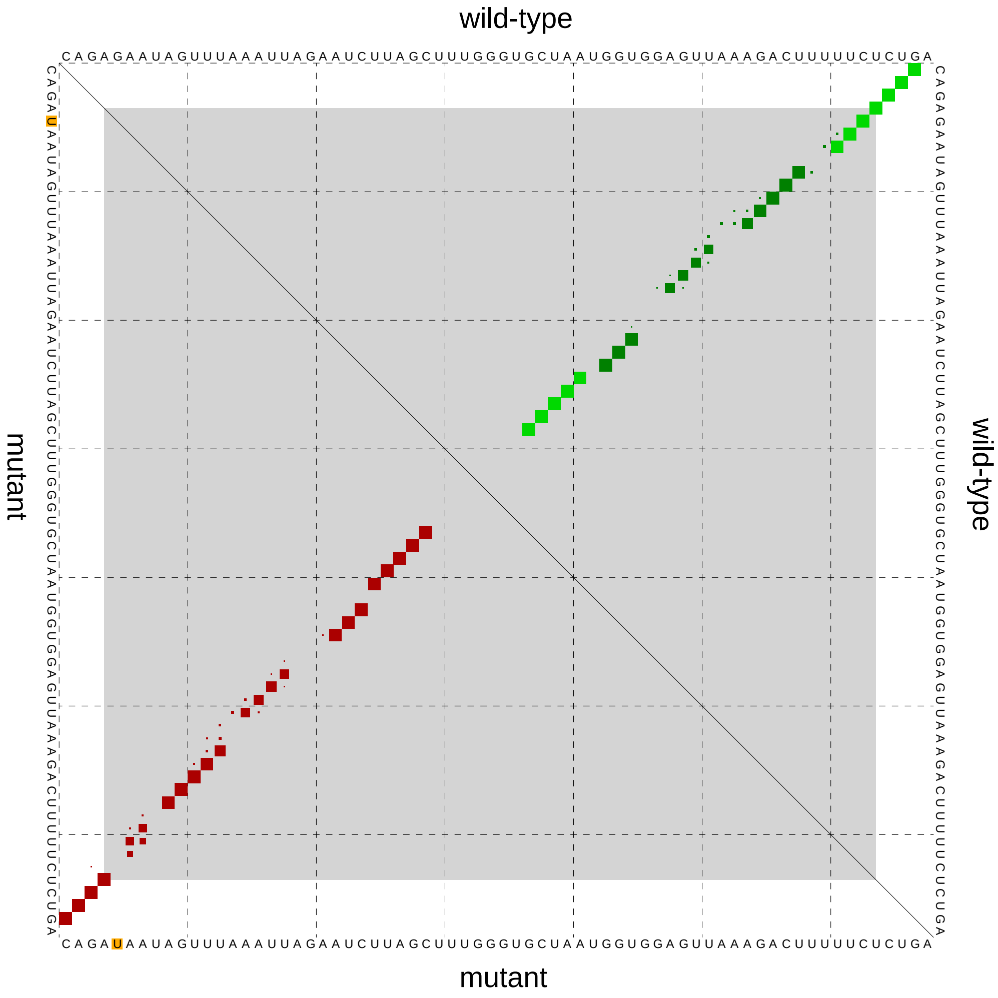

(a) Base-pair probabilities

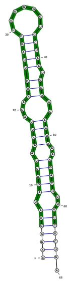

(b) wild-type

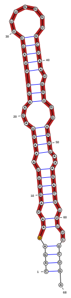

(c) mutant

Ref.	SampleID	chrPos	tRNA	Strand	mut	RNAsnp P-value	nsp
38	TCGA-CV-7180-01	1642	TV	+	G41A	0.5413	no

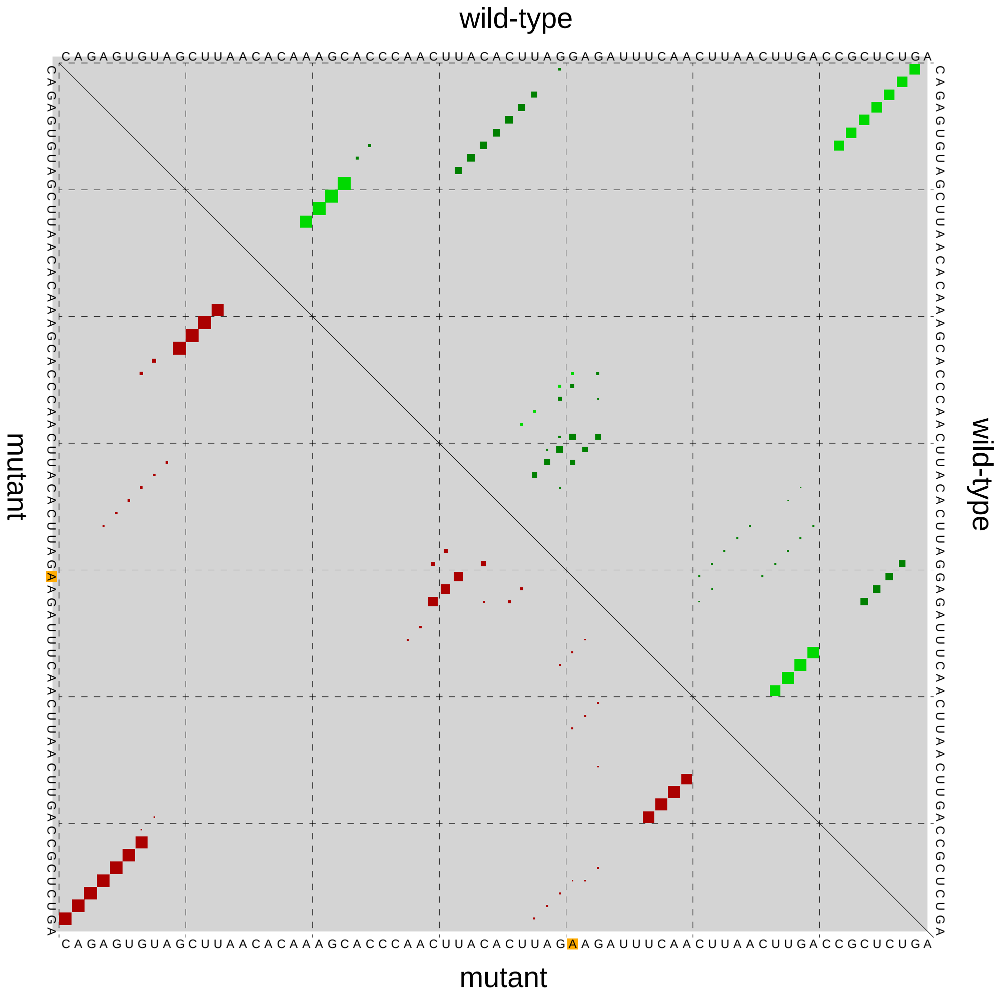

(a) Base-pair probabilities

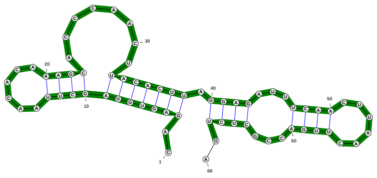

(b) wild-type

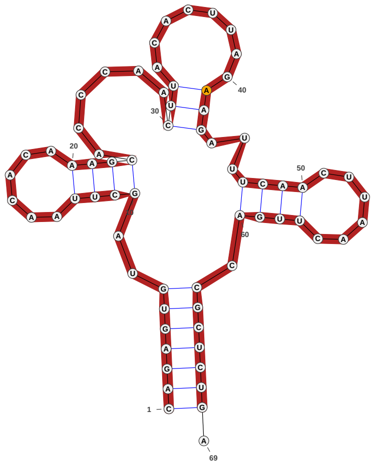

(c) mutant

Ref.	SampleID	chrPos	tRNA	Strand	mut	RNAsnp P-value	nsp
39	TCGA-BA-A4IH-01	622	TF	+	G46A	0.5503	no

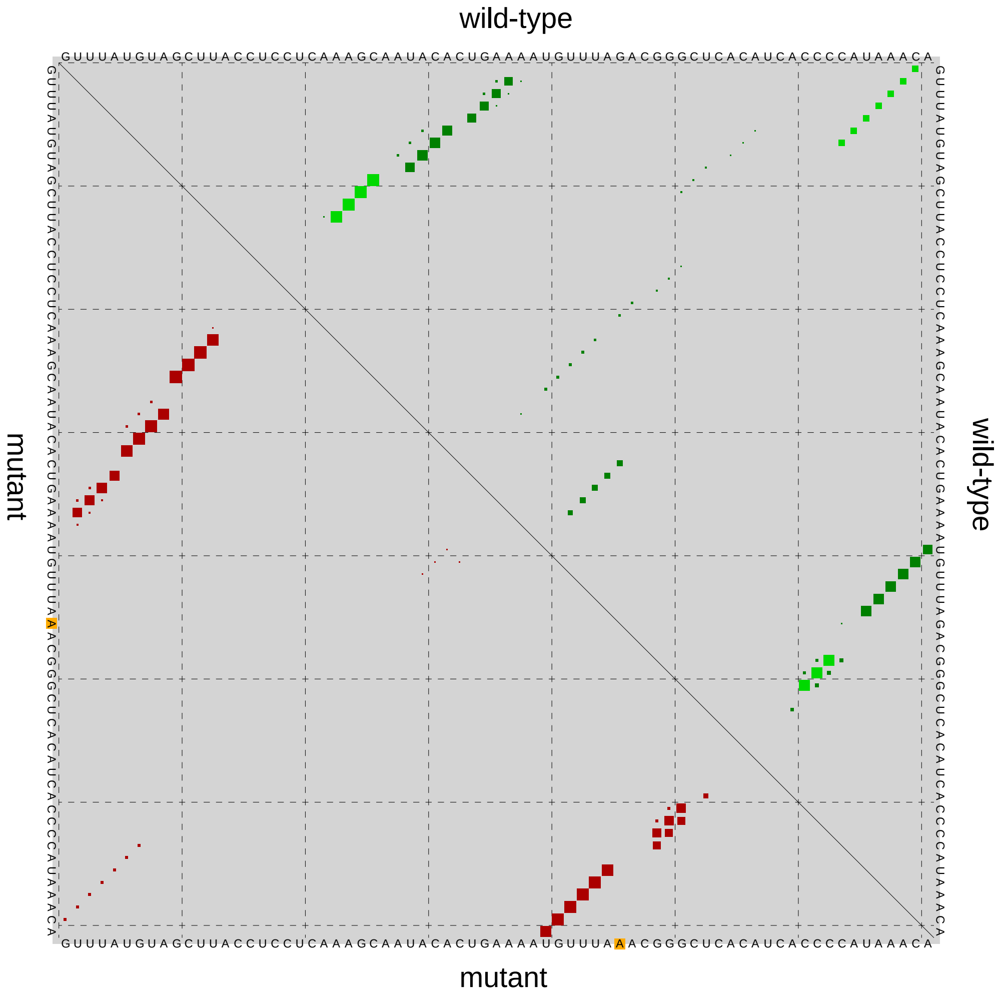

(a) Base-pair probabilities

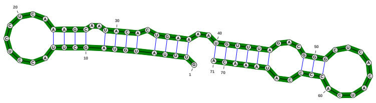

(b) wild-type

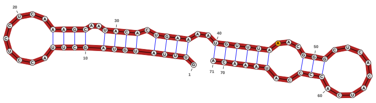

(c) mutant

Ref.	SampleID	chrPos	tRNA	Strand	mut	RNAsnp P-value	nsp
40	TCGA-B6-A0IJ-01	5657	TN	-	G73U	0.6523	no

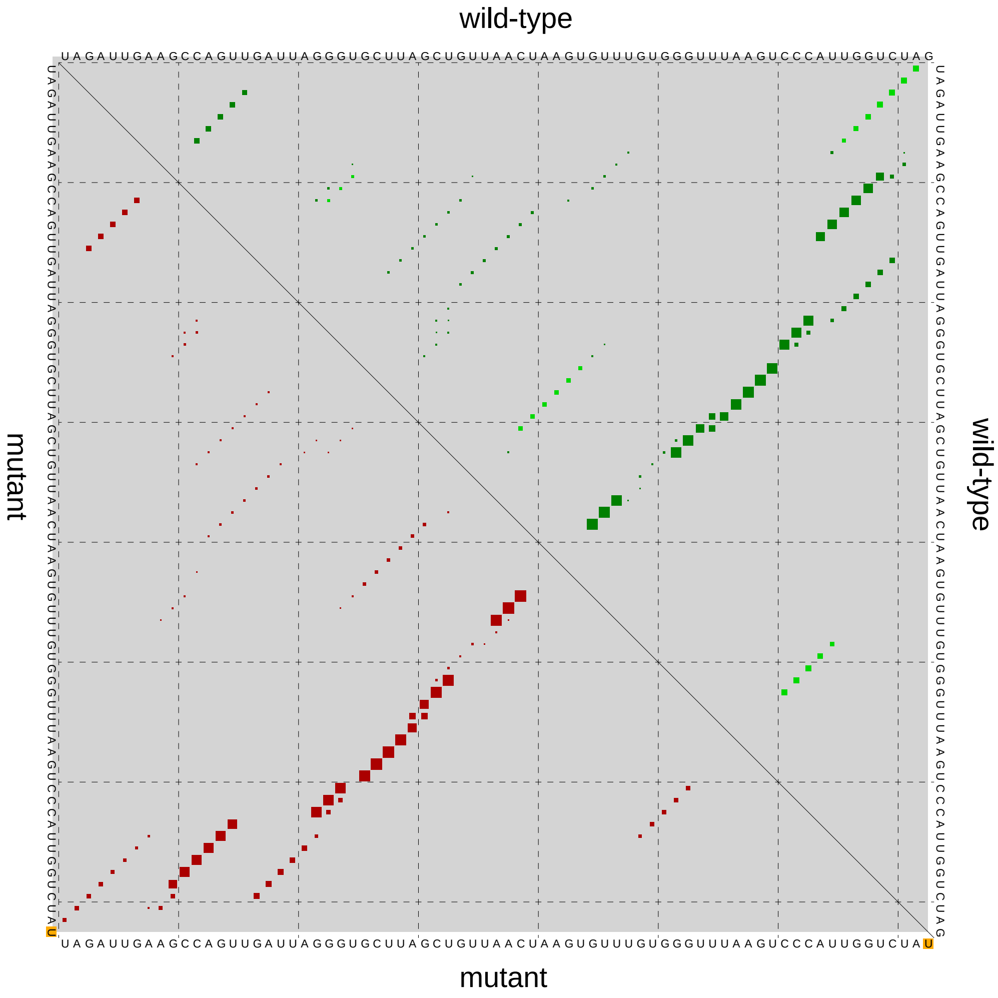

(a) Base-pair probabilities

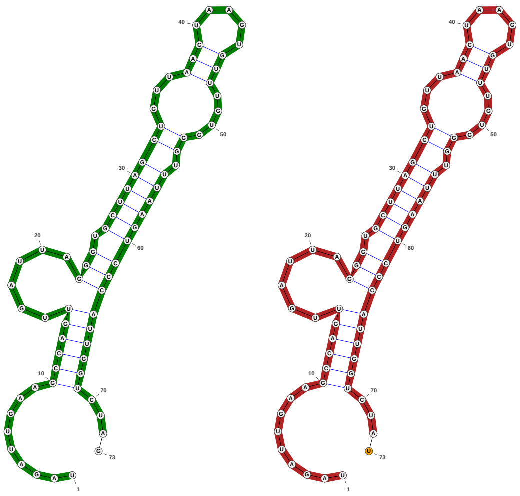

(b) wild-type (c) mutant

Ref.	SampleID	chrPos	tRNA	Strand	mut	RNAsnp P-value	nsp
41	TCGA-AG-4007-01	1641	TV	+	G40A	0.8430	no

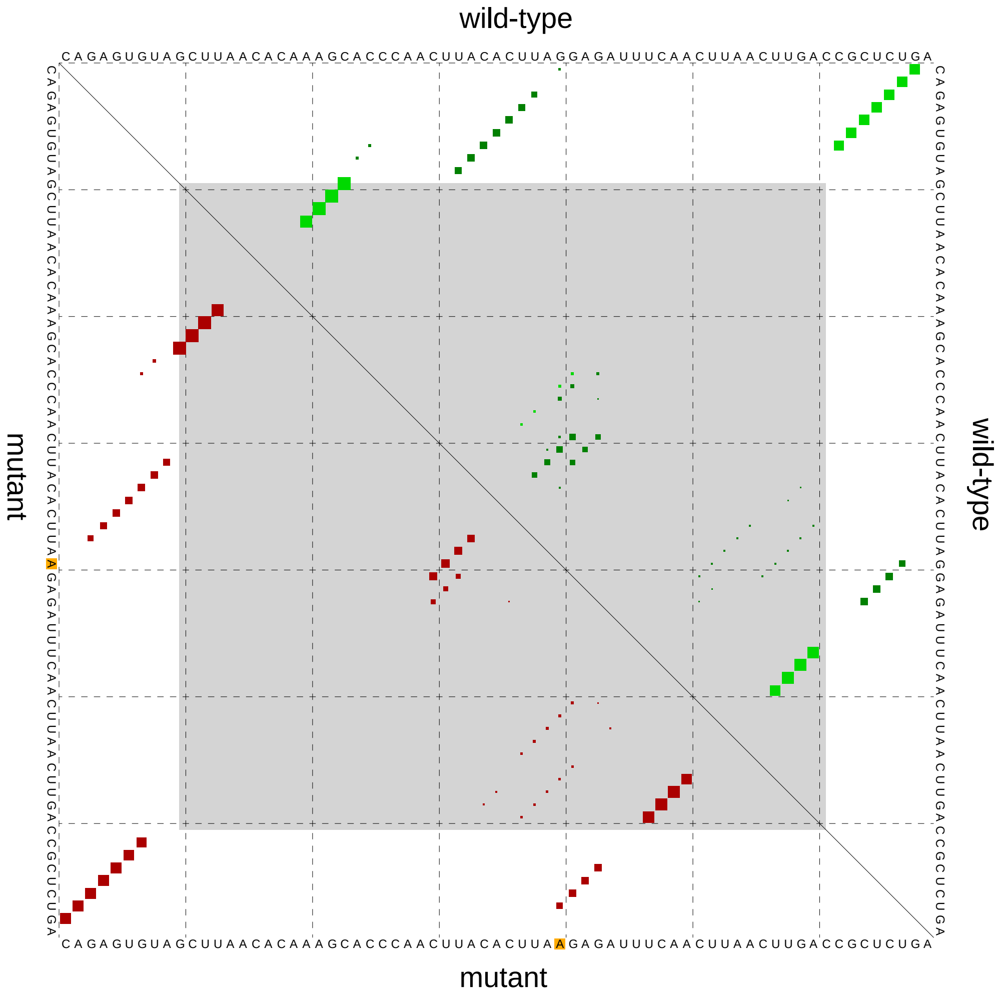

(a) Base-pair probabilities

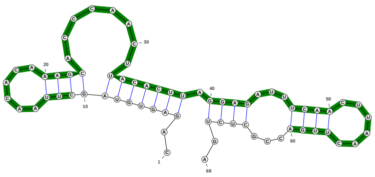

(b) wild-type

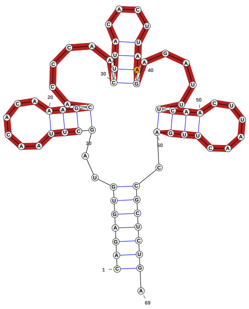

(c) mutant

Ref.	SampleID	chrPos	tRNA	Strand	mut	RNAsnp P-value	nsp
42	TCGA-A2-A0YG-01	15995	TP	-	C29U	0.9327	no

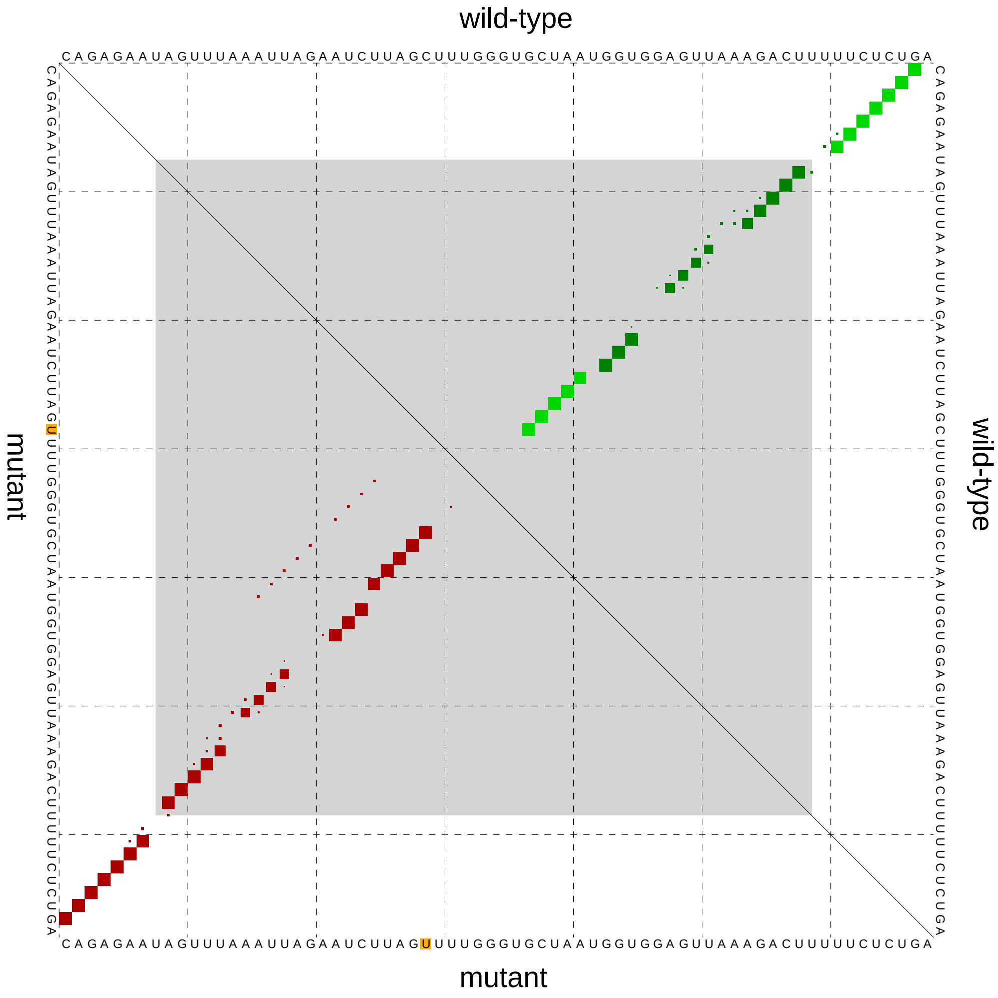

(a) Base-pair probabilities

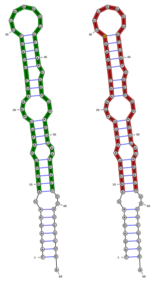

(b) wild-type (c) mutant

Ref.	SampleID	chrPos	tRNA	Strand	mut	RNAsnp P-value	nsp
43	TCGA-A2-A04T-01	3243	TL1	+	A14G	0.9337	no

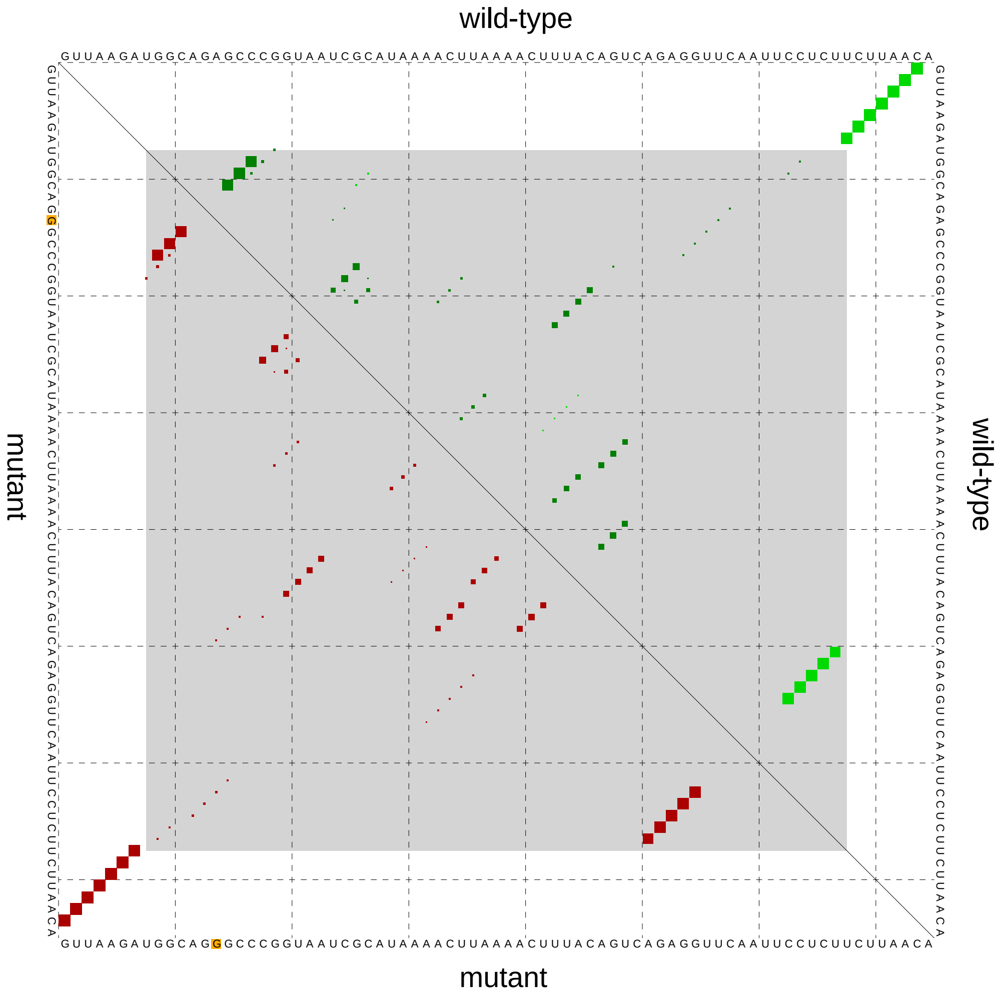

(a) Base-pair probabilities

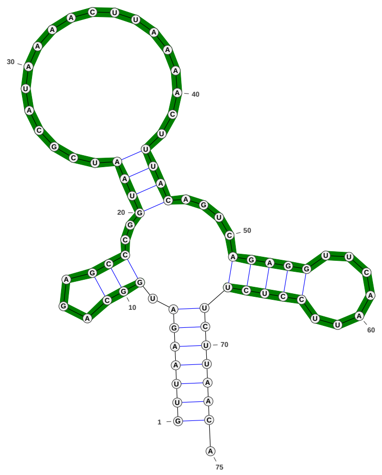

(b) wild-type

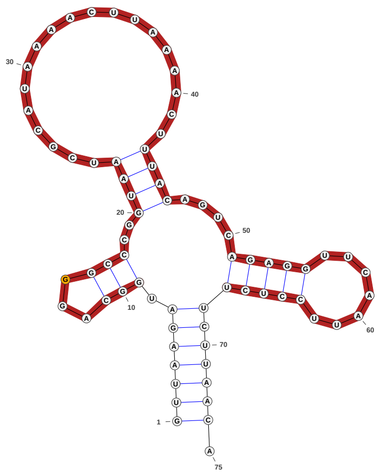

(c) mutant

Ref.	SampleID	chrPos	tRNA	Strand	mut	RNAsnp P-value	nsp
44	TCGA-AC-A2BK-01	15915	TT	+	G28A	0.9477	no

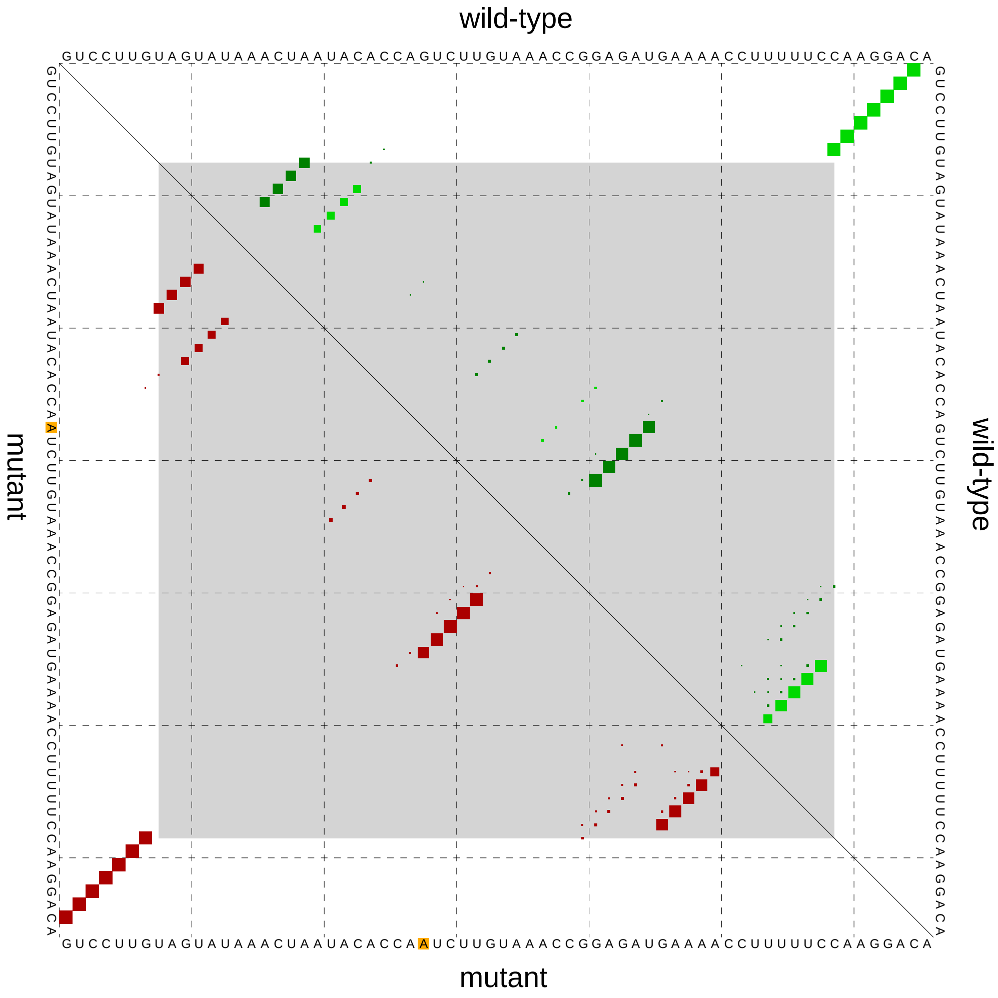

(a) Base-pair probabilities

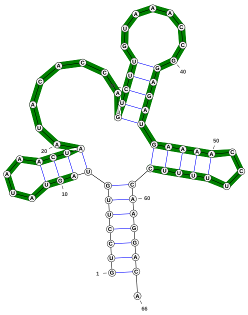

(b) wild-type

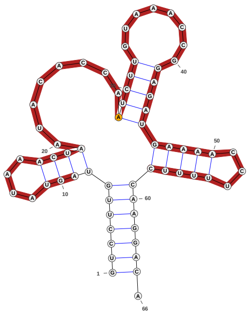

(c) mutant

Ref.	SampleID	chrPos	tRNA	Strand	mut	RNAsnp P-value	nsp
45	TCGA-AO-A0J2-01	12311	TL2	+	U46C	1.0000	no

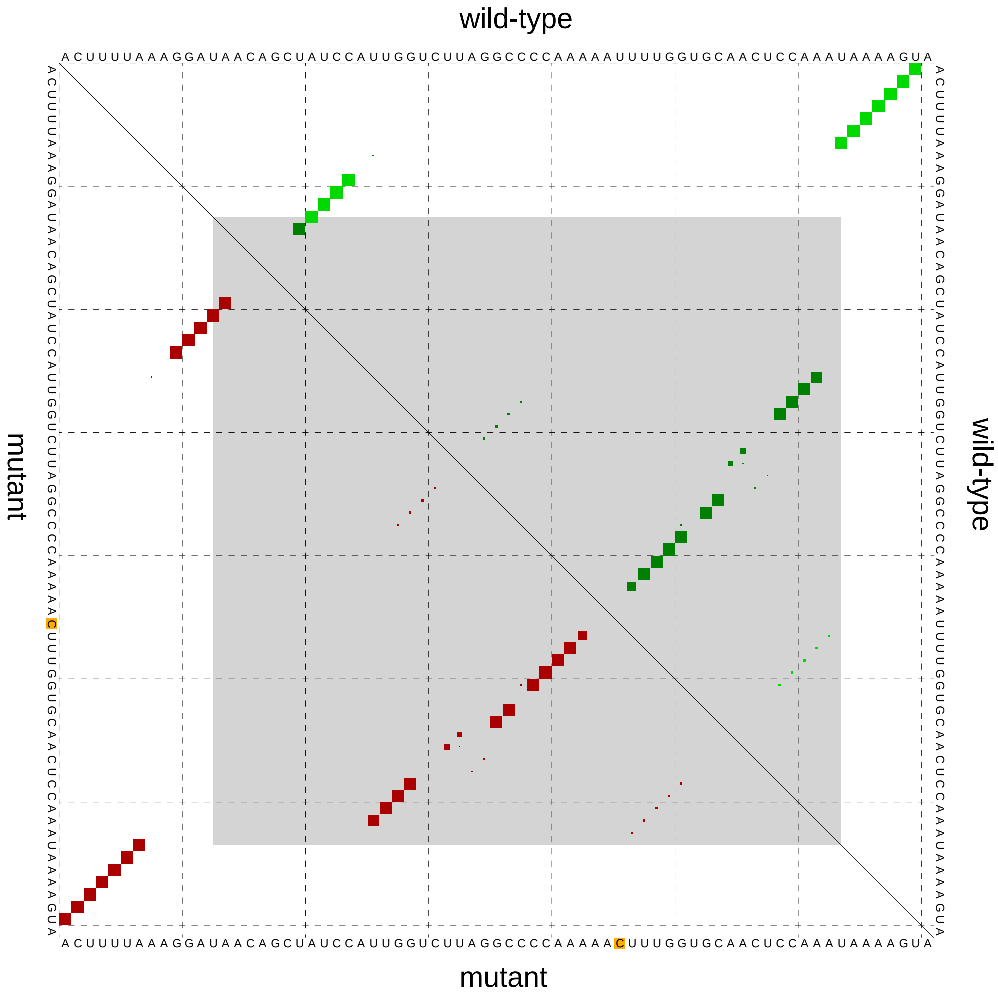

(a) Base-pair probabilities

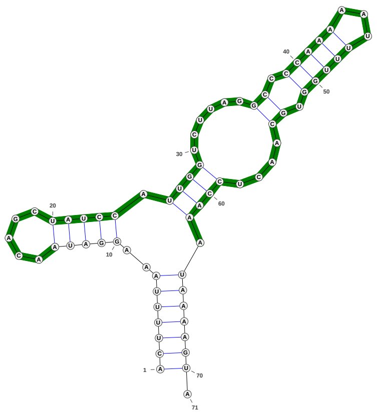

(b) wild-type

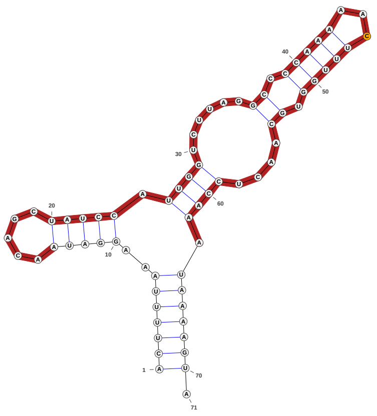

(c) mutant
