# Supplementary material for: Simultaneous DNA and RNA Mapping of Somatic Mitochondrial Mutations across Diverse Human Cancers
Source: PLoS Genet. 2015 Jun 30;11(6):e1005333. doi: 10.1371/journal.pgen.1005333 (PMC4488357; doi:10.1371/journal.pgen.1005333)
Supplement: S4 Fig — The plots show relative (per-tumor normalized) polyA+ expression levels across the mitochondrial genome in mutated regions for additional mutations not included in Fig 4A and 4D. All additional mutations in with allele frequency difference > 0.3 or < -0.3 are shown, as well as all tRNA mutations in the -0.3…0.3 range. All Mutated cases (red) are compared to controls (blue, median of all non-mutated cases). Mutated positions are indicated by triangles. VAFDNA, variant allele frequency in DNA; VAFRNA, variant allele frequency in RNA. (PDF) [file pgen.1005333.s004.pdf]

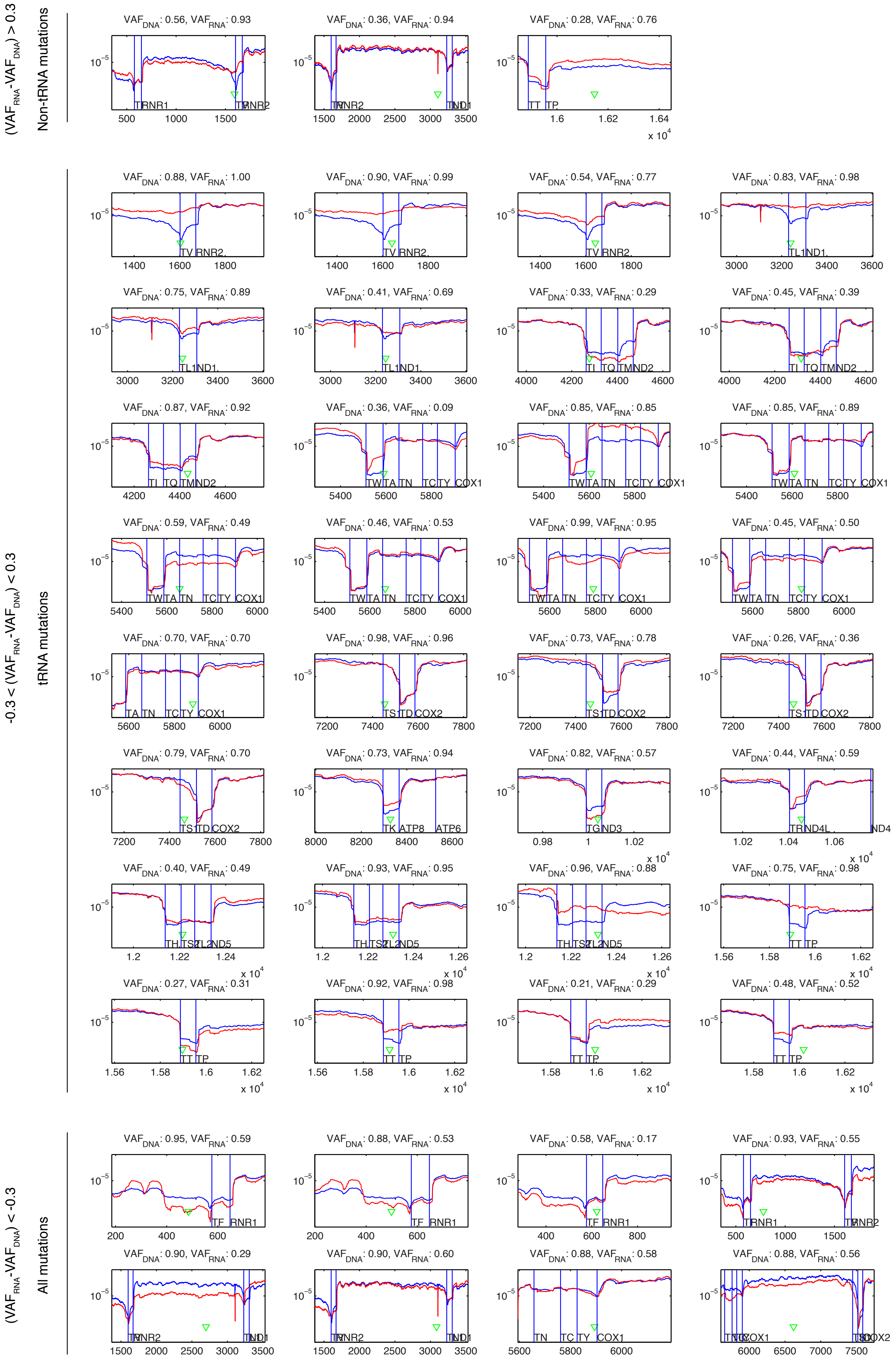

$(VAF_{RNA}-VAF_{DNA}) > 0.3$
Non-tRNA mutations
VAF_{DNA}: 0.56, VAF_{RNA}: 0.93
VAF_{DNA}: 0.36, VAF_{RNA}: 0.94
VAF_{DNA}: 0.28, VAF_{RNA}: 0.76
$-0.3 < (VAF_{RNA}-VAF_{DNA}) < 0.3$
tRNA mutations
VAF_{DNA}: 0.88, VAF_{RNA}: 1.00
VAF_{DNA}: 0.90, VAF_{RNA}: 0.99
VAF_{DNA}: 0.54, VAF_{RNA}: 0.77
VAF_{DNA}: 0.83, VAF_{RNA}: 0.98
VAF_{DNA}: 0.75, VAF_{RNA}: 0.89
VAF_{DNA}: 0.41, VAF_{RNA}: 0.69
VAF_{DNA}: 0.33, VAF_{RNA}: 0.29
VAF_{DNA}: 0.45, VAF_{RNA}: 0.39
VAF_{DNA}: 0.87, VAF_{RNA}: 0.92
VAF_{DNA}: 0.36, VAF_{RNA}: 0.09
VAF_{DNA}: 0.85, VAF_{RNA}: 0.85
VAF_{DNA}: 0.85, VAF_{RNA}: 0.89
VAF_{DNA}: 0.59, VAF_{RNA}: 0.49
VAF_{DNA}: 0.46, VAF_{RNA}: 0.53
VAF_{DNA}: 0.99, VAF_{RNA}: 0.95
VAF_{DNA}: 0.45, VAF_{RNA}: 0.50
VAF_{DNA}: 0.70, VAF_{RNA}: 0.70
VAF_{DNA}: 0.98, VAF_{RNA}: 0.96
VAF_{DNA}: 0.73, VAF_{RNA}: 0.78
VAF_{DNA}: 0.26, VAF_{RNA}: 0.36
VAF_{DNA}: 0.79, VAF_{RNA}: 0.70
VAF_{DNA}: 0.73, VAF_{RNA}: 0.94
VAF_{DNA}: 0.82, VAF_{RNA}: 0.57
VAF_{DNA}: 0.44, VAF_{RNA}: 0.59
VAF_{DNA}: 0.40, VAF_{RNA}: 0.49
VAF_{DNA}: 0.93, VAF_{RNA}: 0.95
VAF_{DNA}: 0.96, VAF_{RNA}: 0.88
VAF_{DNA}: 0.75, VAF_{RNA}: 0.98
VAF_{DNA}: 0.27, VAF_{RNA}: 0.31
VAF_{DNA}: 0.92, VAF_{RNA}: 0.98
VAF_{DNA}: 0.21, VAF_{RNA}: 0.29
VAF_{DNA}: 0.48, VAF_{RNA}: 0.52
$(VAF_{RNA}-VAF_{DNA}) < -0.3$
All mutations
VAF_{DNA}: 0.95, VAF_{RNA}: 0.59
VAF_{DNA}: 0.88, VAF_{RNA}: 0.53
VAF_{DNA}: 0.58, VAF_{RNA}: 0.17
VAF_{DNA}: 0.93, VAF_{RNA}: 0.55
VAF_{DNA}: 0.90, VAF_{RNA}: 0.29
VAF_{DNA}: 0.90, VAF_{RNA}: 0.60
VAF_{DNA}: 0.88, VAF_{RNA}: 0.58
VAF_{DNA}: 0.88, VAF_{RNA}: 0.56
10^{-5}
$x\ 10^4$
