# Supplementary material for: Simultaneous DNA and RNA Mapping of Somatic Mitochondrial Mutations across Diverse Human Cancers
Source: PLoS Genet. 2015 Jun 30;11(6):e1005333. doi: 10.1371/journal.pgen.1005333 (PMC4488357; doi:10.1371/journal.pgen.1005333)
Supplement: S1 Appendix — (PDF) [file pgen.1005333.s006.pdf]

S1 Appendix. Comparison of observed synonymous:nonsynonymous ratios to an expected null model.

The classic test of positive selection involves a comparison of the observed nonsynonymous substitutions per synonymous site to the observed synonymous substitutions per synonymous site (the *dN/dS* test). This test was originally employed to test the fixed substitutions that occurred in in protein-coding sequences from district, divergent lineages[1]. Under these conditions, *dN/dS* < show sequences under purifying selection, *dN/dS* >1 signifies sequences under positive selection, and *dN/dS* = 1 demonstrates neutral evolution of a give sequence. Recent work has expanded the use of *dN/dS* tests to mitochondrial mutations within human tumours [2]. However, the test has been demonstrated to not be robust for use with within-populations comparisons [3,4], and mitochondrial heteroplasmy is in violation of the assumption of fixed polymorphisms.

Instead, we obtained the codon usage table for the reference rCRS human mitochondrial sequence, for the protein genes encoded on each strand separately, using CodonW [5]. With this, we were able to calculate all possible substitution mutations, and determine whether the resulting changes would synonymous or non-synonymous. Indel mutations within the protein genes were not included in our analysis, as we detected a signature of purifying selection against this class of mutations (Figure 2).

The mutational profile we observed was found to have a strong mutational bias, including a strand-specific preference for the nucleotides undergoing the mutation. The bias appears quite similar to that observed in non-tumour samples from human tissues [6,7], and other published studies of somatic mutations in tumors [2,8]. Thus, we extracted the mutational spectrum of all nonsense, missense and synonymous mutations from the 13 protein coding genes from this study.

	A>C	A>G	A>T	C>A	C>G	C>T	G>A	G>C	G>T	T>A	T>C	T>G
**This Study**	0.0089	0.0386	0	0.0119	0.0030	0.1128	0.5045	0.0119	0	0	0.3027	0.0059
**[2]**	0	0.0388	0	0.0155	0.0078	0.0853	0.5969	0.0233	0.0078	0	0.2248	0
**[8]**	0.0156	0.0720	0.0130	0.0312	0.0035	0.0694	0.4666	0.0095	0.0069	0.0026	0.3062	0.0035

This mutational bias was then used to weight the probability of a given base mutating in the codon table generated from each strand. We then assessed the probability of these weighted mutations causing synonymous or non-synonymous changes to the resulting protein sequences. These were used to generate out expect values for the null hypothesis. In a sample under this mutation bias, the proportion of non-synonymous mutations is expected to be 0.7357 versus 0.2643 for synonymous mutations. For the 337 missense, nonsense and synonymous mutations observed, we would expect 248 nonsynonymous and 89 synonymous mutations. Our data set of 69 synonymous and 268 missense + nonsense mutations fails to reject the null ($P = 0.0841$, Chi-squared with Yates' correction).

We also used this method to calculate the expected values based on previous studies, using mutation biases described for each study [2,8].

Paper	Observed Nonsynonymous	Observed Synonymous	Expected Nonsynonymous	Expected Synonymous	***P***
**This Study**	268	69	248	89	0.0841
**Larman et al. [8]**	103	24	96	31	0.3607
**Ju et al. [2]**	878	208	810	276	0.0006

Using this method, only the data from [2] show evidence of excess nonsynonymous mutations. However, in their paper, they utilized a likelihood analysis of a Poisson-based sampling process, and failed to show variation from random segregation, using this more complex model.

Additional References

1. Yang Z, Bielawski JP (2000) Statistical methods for detecting molecular adaptation. Trends Ecol Evol 15: 496-503.
2. Ju YS, Alexandrov LB, Gerstung M, Martincorena I, Nik-Zainal S, et al. (2014) Origins and functional consequences of somatic mitochondrial DNA mutations in human cancer. Elife 3: 10.7554/eLife.02935.
3. Kryazhimskiy S, Plotkin JB (2008) The population genetics of dN/dS. PLoS Genet 4: e1000304.
4. Mugal CF, Wolf JB, Kaj I (2014) Why time matters: codon evolution and the temporal dynamics of dN/dS. Mol Biol Evol 31: 212-231.
5. Peden JF (2005) CodonW codon usage analysis package.
6. Kennedy SR, Salk JJ, Schmitt MW, Loeb LA (2013) Ultra-sensitive sequencing reveals an age-related increase in somatic mitochondrial mutations that are inconsistent with oxidative damage. PLoS Genet 9: e1003794.
7. Williams SL, Mash DC, Züchner S, Moraes CT (2013) Somatic mtDNA Mutation Spectra in the Aging Human Putamen. PLoS Genet 9: e1003990.
8. Larman TC, DePalma SR, Hadjipanayis AG, Protopopov A, Zhang J, et al. (2012) Spectrum of somatic mitochondrial mutations in five cancers. Proc Natl Acad Sci U S A 109: 14087-14091.
