# Supplementary material for: Simultaneous DNA and RNA Mapping of Somatic Mitochondrial Mutations across Diverse Human Cancers
Source: PLoS Genet. 2015 Jun 30;11(6):e1005333. doi: 10.1371/journal.pgen.1005333 (PMC4488357; doi:10.1371/journal.pgen.1005333)
Supplement: S1 Table — DNA and RNA allele frequencies are indicated, as well as predicted structural impact determined using RNAsnp. The column “Native structure predicted” represents whether the wild-type secondary structure predicted by RNAsnp match the reference tRNA secondary structure (clover-leaf structure) [37,46] (see S3 Fig for more details). (PDF) [file pgen.1005333.s009.pdf]

Supplementary Table 1. Somatic mutations in mt-tRNAs.

Tumor	ChrM Pos	tRNA	Strand	tRNA position	Mutation	DNA allele frequency	RNA allele frequency	RNAsnp P-value$	Native structure predicted
TCGA-A2-A04T-01	5881	TY	-	56	G->A	0.7	0.7	0.0447	yes
TCGA-DK-A1AG-01	4328	TI	+	66	T->C	0.5	0.99	0.074	yes
TCGA-DK-A3IL-01	4299	TI	+	37	T->C	0.24	0.98	0.1213	yes
TCGA-D1-A17K-01	4412	TM	+	11	G->A	0.67	1	0.1743	yes
TCGA-AX-A0J1-01	4412	TM	+	11	G->A	0.28	0.98	0.1743	yes
TCGA-EY-A1GW-01	4326	TI	+	64	T->C	0.51	0.99	0.18	yes
TCGA-BP-4977-01	4423	TM	+	22	T->C	0.26	0.71	0.2137	yes
TCGA-AP-A05A-01	7453	TS1	-	8	G->A	0.98	0.96	0.2803	yes
TCGA-DJ-A2Q2-01	4316	TI	+	54	A->G	0.45	0.39	0.3337	yes
TCGA-BH-A0WA-01	4449	TM	+	48	G->A	0.47	0.97	0.346	yes
TCGA-67-3772-01	4282	TI	+	20	G->A	0.55	1	0.4393	yes
TCGA-DA-A1HV-06	4435	TM	+	34	A->G	0.87	0.92	0.4967	yes
TCGA-60-2719-01	5610	TA	-	24	G->A	0.85	0.89	0.6013	yes
TCGA-EJ-5506-01	7566	TD	+	49	G->A	0.21	0.58	0.6307	yes
TCGA-AA-3555-01	7465	TS1	-	20	A->+C	0.73	0.78	n/a*	n/a*
TCGA-A2-A04Q-01	7465	TS1	-	20	A->+C	0.26	0.36	n/a*	n/a*
TCGA-CN-4741-01	7465	TS1	-	20	A->+C	0.79	0.7	n/a*	n/a*
TCGA-05-5429-01	5609	TA	-	23	T->C	0.85	0.85	0.8757	yes
TCGA-06-0744-01	5590	TA	-	4	G->A	0.36	0.09	0.936	yes
TCGA-B2-4099-01	4277	TI	+	15	T->C	0.33	0.29	0.9503	yes
TCGA-BP-4781-01	10042	TG	+	52	A->G	0.82	0.57	0.9713	yes
TCGA-EM-A3AL-01	3244	TL1	+	15	G->A	0.41	0.69	0.4013	no
TCGA-CR-6470-01	3294	TL1	+	65	T->C	0.42	0.92	0.0533	no
TCGA-DA-A3F5-06	586	TF	+	10	G->A	0.52	0.95	0.071	no
TCGA-60-2724-01	1604	TV	+	3	G->A	0.88	1	0.0917	no
TCGA-ET-A4KN-01	12319	TL2	+	54	C->T	0.96	0.88	0.101	no
TCGA-05-4398-01	15892	TT	+	5	T->C	0.75	0.98	0.105	no
TCGA-AP-A05A-01	5791	TC	-	31	G->A	0.99	0.95	0.1893	no
TCGA-DK-A1AA-01	5668	TN	-	12	G->A	0.46	0.53	0.2697	no
TCGA-EL-A3CV-01	3239	TL1	+	10	G->A	0.83	0.98	0.303	no
TCGA-DK-A1AA-01	10457	TR	+	53	T->C	0.44	0.59	0.3203	no
TCGA-KN-8424-01	15897	TT	+	10	G->A	0.27	0.31	0.3797	no
TCGA-CV-6961-01	5571	TW	+	60	T->C	0.38	0.74	0.4	no
TCGA-CW-6087-01	5814	TC	-	54	T->C	0.45	0.5	0.4267	no
TCGA-05-4396-01	12213	TS2	+	7	G->A	0.4	0.49	0.438	no
TCGA-A2-A3Y0-01	8328	TK	+	34	G->A	0.73	0.94	0.4443	no
TCGA-A8-A092-01	16019	TP	-	64	C->A	0.48	0.52	0.5093	no
TCGA-CV-7180-01	1642	TV	+	41	G->A	0.54	0.77	0.5413	no
TCGA-BA-A4IH-01	622	TF	+	46	G->A	0.58	0.17	0.5503	no
TCGA-B6-A0IJ-01	5657	TN	-	1	C->A	0.59	0.49	0.6523	no

TCGA-AG-4007-01	1641	TV	+	40	G->A	0.9	0.99	0.843	no
TCGA-A2-A0YG-01	15995	TP	-	40	G->A	0.21	0.29	0.9327	no
TCGA-A2-A04T-01	3243	TL1	+	14	A->G	0.75	0.89	0.9337	no
TCGA-AC-A2BK-01	15915	TT	+	28	G->A	0.92	0.98	0.9477	no
TCGA-AO-A0J2-01	12311	TL2	+	46	T->C	0.93	0.95	1	no

[$] The *P*-values were obtained using background scores adapted to tRNAs (see Methods).

[*] Indels cannot be handled by RNAsnp.
