# Supplementary material for: Simultaneous DNA and RNA Mapping of Somatic Mitochondrial Mutations across Diverse Human Cancers
Source: PLoS Genet. 2015 Jun 30;11(6):e1005333. doi: 10.1371/journal.pgen.1005333 (PMC4488357; doi:10.1371/journal.pgen.1005333)
Supplement: S2 Table — (PDF) [file pgen.1005333.s010.pdf]

Supplementary Table 2. Enrichment analysis of structure-disruptive mutations in the processing defect group compared to no processing defect group, under different RNAsnp *P*-value cut-offs.

RNAsnp *P*-value cut-offs	Processing defect- "Yes" (total mutations = 9)	Procesing defect- "No" (total mutations = 9)	One-sided Fisher's exact test (*P*-value)
	No. of mutations under the chosen *P*-value cut-off		
< 0.10	1	1	0.7647
<0.15	2	1	0.5000
<0.20	5	1	0.0656
<0.25	6	1	0.0249
<0.30	6	2	0.0767
<0.35	7	3	0.0767
<0.40	7	3	0.0767
<0.45	8	3	0.0249
<0.50	8	4	0.0656
